# How does equity restriction affect innovation quality? Evidence from listed manufacturing companies in China

**DOI:** 10.1371/journal.pone.0295553

**Published:** 2023-12-07

**Authors:** Sang Chang, Jie Wu, Muhammad Riaz, Zhizhong Hu

**Affiliations:** 1 School of Economics and Management, Jiangsu University of Science and Technology, Zhenjiang, Jiangsu, China; 2 Technology Development Department, Zhong Zhi (Beijing) Certification Co., LTD, Beijing, China; The University of Hong Kong, HONG KONG

## Abstract

Improving the innovation quality of manufacturing companies has currently received increasing attention in the transformation from "Made in China" to "Created in China". Equity restriction is now one of the most talked about issues in China’s corporate governance structures, but we have a limited understanding of the impact of equity restriction on innovation quality. This paper empirically analyzes the relationship and intrinsic mechanism between equity restriction and innovation quality using all A-share listed manufacturing companies in China from 2007 to 2021 as the research sample. First, the Tobit regression model is used to analyze the impact of equity restriction on innovation quality, and the Heckman two-stage model is used for the endogenous test. Then, the three-step regression model with mediating effects is used to validate the intrinsic mechanism of equity restriction to promote innovation quality from two paths, namely equity incentives and R&D investment. The research results show that equity restriction has a significant positive impact on innovation quality; equity incentives and R&D investment play a mediating role between equity restriction and innovation quality. This paper enriches the research on the influencing factors of innovation quality and provides a theoretical basis based on equity restriction for the transformation of manufacturing towards high-quality innovation, and explores the intrinsic mechanisms by which equity restriction affects innovation quality.

## 1. Introduction

**Fig 1:**
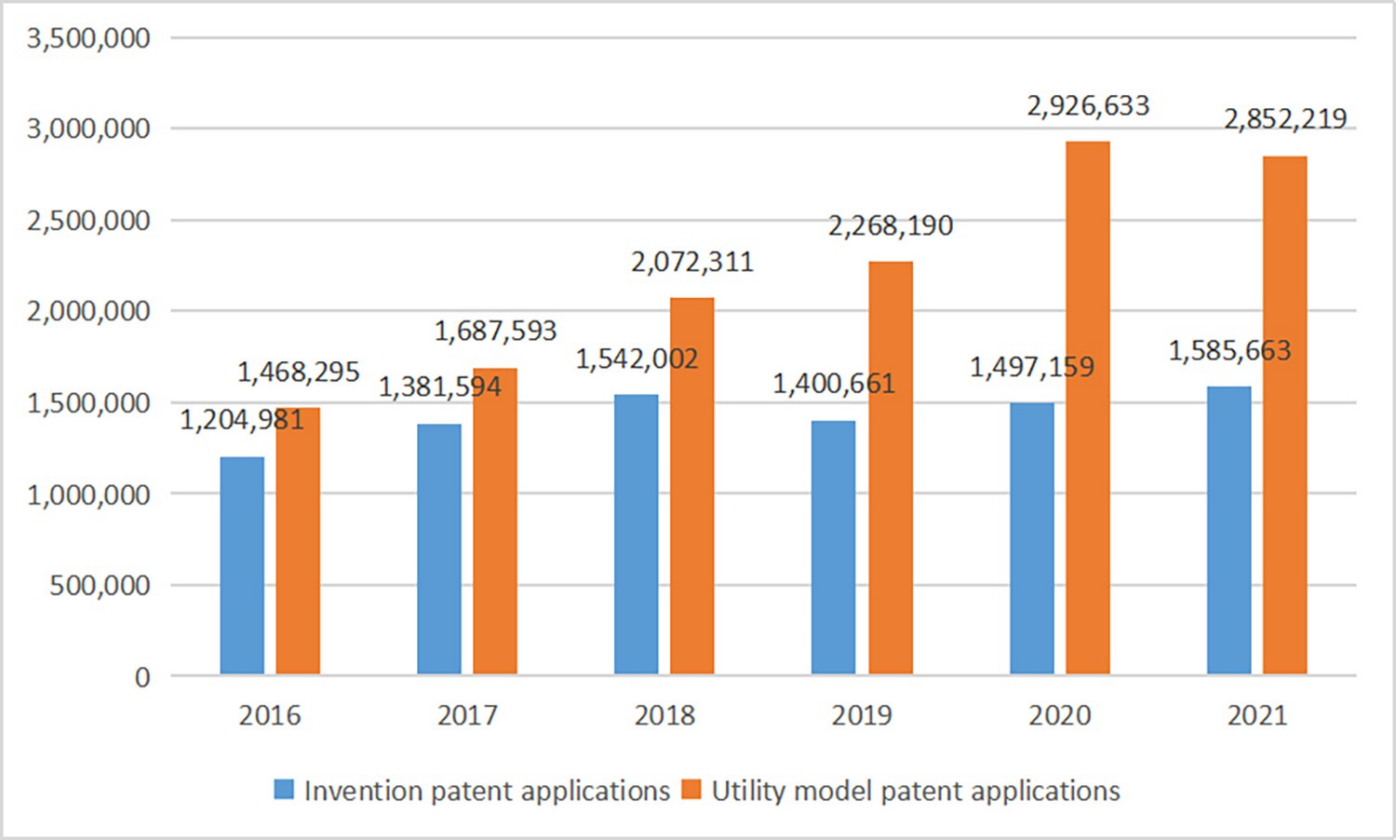
Comparison between the number of invention patents and utility model patent applications in China from 2016 to 2021.

The Chinese government issued the "National Innovation-Driven Development Strategy Outline" in 2016, pointing out that "innovation is crucial to the country’s destiny" and "innovation should be made the primary driving force for economic development." and "China is expected to develop into the world’s leading powerhouse in scientific and technological innovation by 2050." Patents are one of the most direct results of innovation activities and are often used as indicators of corporate innovation [1]. Judging from the number of patents, China is truly an innovation powerhouse. According to the "World Intellectual Property Indicators 2022" report released by WIPO, China accounted for 46.6% of all patent applications worldwide in 2021, ranking first in the world. However, the number of patents is not equivalent to the quality of patents, and there is still much room for improvement in innovation quality in China. In reality, instead of applying for higher-quality invention patents based on actual research results, some Chinese companies tend to apply for low-quality patents [2], such as a large number of utility model patents or invalid patents, to seek stronger strategic competitiveness and more government patent subsidies [3]. Fig 1 compares the number of utility model and innovation patent applications in China between 2016 and 2021. It can be seen from the figure that the number of utility model patent applications in China far exceeded that of invention patent applications, which had resulted in a large gap between innovation quality and innovation quantity [4]. However, most of the current research has focused on the number of innovations, with relatively little research on the factors influencing the innovation quality.

**Fig 2:**
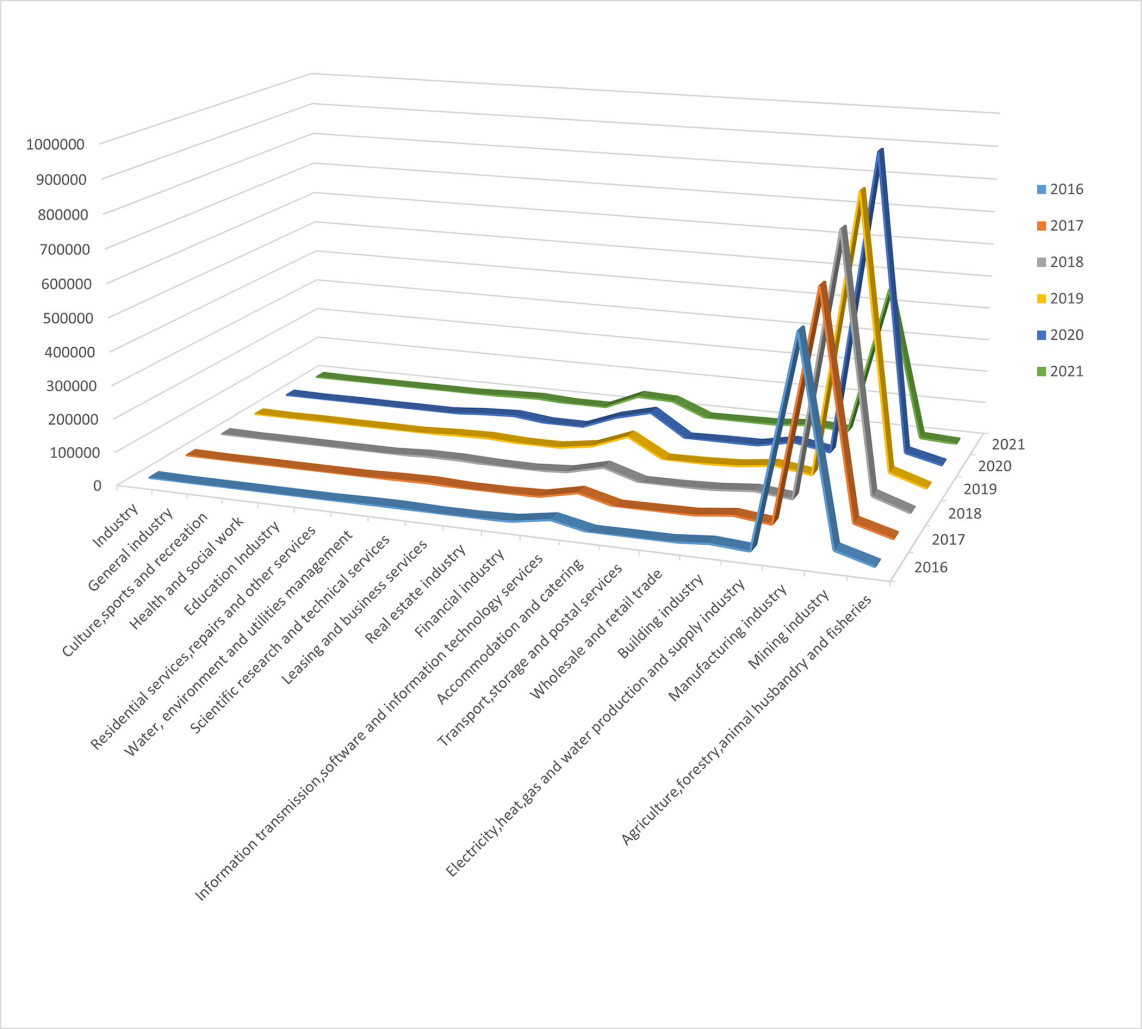
The distribution of the number of innovations among Chinese companies in different industries from 2016 to 2021.

Manufacturing is an important pillar of the national economy, especially in the Chinese economy. Since 2011, China has accounted for 19.8 percent of global manufacturing value added [5]. China now continues to maintain its position as the world’s largest manufacturing country. As China is a major manufacturing country, the quality of innovation in manufacturing companies is crucial to improving the overall level of innovation in China [6] [7]. Fig 2 shows the distribution of the number of innovations among Chinese companies in different industries from 2016 to 2021, where the number of patent applications is used as a measure of the number of innovations [1]. As can be seen from the figure, the number of innovations in the manufacturing industry is significantly higher than that in other industries. Therefore, based on the huge number of innovations, studying how to improve the quality of innovation in Chinese manufacturing companies is of great practical significance for accelerating the development of the manufacturing industry in the direction of high quality, and it can also play an important role in promoting China’s overall innovation quality.

The impact of equity restriction on corporate innovation is one of the most important issues currently being focused on in China’s corporate governance structure [8]. However, there is little research on how equity restriction affects innovation quality. Equity restriction, which refers to a kind of equity structure where multiple major shareholders restrict each other, has always been regarded as an important factor influencing corporate innovation decision-making and is a concentrated embodiment of stakeholders’ rights and obligations, directly determining a company’s resource allocation and decision-making process [9–12]. How to promote corporate innovation by improving the level of equity restriction has become the focus of scholars’ attention [13]. In the socialist market economy system with Chinese characteristics, equity concentration is the main feature of listed manufacturing companies in China. However, the corporate governance brought about by excessively concentrated equity results in the conflict of interests between major and minor shareholders. A key strategy for resolving such conflict is equity restriction, which also fosters company innovation [14]. However, the link between equity restriction and innovation is mostly studied from the perspective of innovation investment [15]. For example, Belloc [16] believes that companies with low equity restriction may require higher R&D costs, which potentially reduces innovation investment and hinders R&D activities. Haner [17] is the first to propose the concept of innovation quality and construct the basic framework of innovation quality, which has entered theoretical and academic perspectives since its proposal. However, there is less research on the relationship between equity restriction and innovation quality [18]. Therefore, this paper focuses on the relationship between equity restriction and innovation quality, aiming to provide experience and evidence for improving the governance structure of listed companies and promoting the improvement of corporate innovation quality.

The main contributions of this paper are listed as follows: First, there is relatively little research on the impact of equity restriction on innovation quality in existing literature but more on the relationship between equity restriction and innovation investment. Therefore, this paper focuses on innovation quality using the number of invention patents, a good measure of high-quality innovation output by Chinese companies, intending to provide new evidence for the link between equity restriction and innovation quality and enrich the research on the factors affecting innovation quality. This paper provides a theoretical rationale based on equity restriction for the shift to high-quality innovation in manufacturing firms. Second, there is relatively little research on the role mechanism of equity restriction on innovation quality in the existing literature. Therefore, to explore the pathways through which equity restriction affects innovation quality, this paper uses equity incentives and R&D investment as mediating variables to validate the intrinsic mechanisms by which equity restriction promotes innovation quality. Third, the Heckman two-stage model is adopted to control the potential endogeneity between variables in this paper to provide a more robust conclusion for the relationship between equity restriction and innovation quality.

This paper is structured as follows: The literature review and research hypothesis are presented in Part 2. Research design is presented in Part 3. Results are given in Part 4 and discussed in Part 5. In Part 6, conclusions and suggestions are offered.

## 2. Literature review and research hypothesis

### 2.1 The influence of equity restriction on innovation quality

Equity restriction refers to a kind of equity structure where multiple major shareholders restrict each other [19]. In the study of corporate governance, equity restriction, and their internal agency problems have always been the focus of academic attention [20]. The principal-agent theory holds that equity restriction can help solve two types of principal-agent problems in corporate governance and influence corporate innovation behavior by affecting the allocation of corporate resources [21]. Equity restriction, as an equilibrium state of equity structure, emerges when equity concentration exceeds corporate innovation activities, which helps to alleviate the adverse effect of equity concentration on corporate innovation [22]. A high shareholding of other major shareholders can prevent emptying by the first major shareholder and mitigate the risks brought by the diversification of the company’s investment in innovative projects [23–25]. A sound structure of restriction and supervision among shareholders is conducive to corporate innovation investment and plays a crucial role in enhancing the long-term value of a company [9].

According to Kahn and Winton [26], equity restriction plays a role in limiting the first largest shareholder’s abuse of their control rights for private benefits. Furthermore, with the increase of supervisory shareholders’ voice in the company, their interests tend to be consistent with those of the company, thus reducing agency costs. Monitoring shareholders are more motivated to optimize the allocation of corporate resources and increase innovation investment. Balance among shareholders avoids a large dominant shareholder and leads to more scientific and professional decision-making in the firm [27]. As the level of equity balance increases, the incentives and ability of external block holders to monitor the behavior of the largest shareholders and executives will be improved [28]. When external blockholders’ rights to speak at the firm increase, their interests will become more aligned with those of the firm, which can motivate and monitor managers’ selection of innovative projects [29].

Equity restriction can help solve the principal-agent problem in corporate governance, reduce the agency cost, promote the improvement of corporate governance structure, make corporate operation more scientific and efficient, and improve the allocation efficiency of corporate resources, thus improving innovation quality. In addition, equity restriction enhances the supervision between major shareholders, making corporate governance more scientific and efficient and creating favorable conditions for improving innovation quality. This study proposes the following hypothesis in light of the above analysis:

Hypothesis 1 (H1): Equity restriction has a significant positive impact on innovation quality in manufacturing companies.

### 2.2 Mediating effect of equity incentives

Equity incentive is a long-term incentive contract based on the pricing by management based on the external capital market [30]. According to the principal-agent theory, when the level of equity restriction increases, the board of directors (or the compensation committee) of the company tends to implement management holding shares and equity incentives for management, so that the goals of management and shareholders are consistent and agency cost is effectively reduced [31]. The agency problem between managers and shareholders can be improved by giving managers residual earnings right through equity incentives [32]. Jun Zhou [33] points out that as the level of equity restriction of listed companies increases, shareholders’ goals tend to be consistent with the company’s development goals, and shareholders tend to give certain equity incentives to management to alleviate insufficient liquidity and reduce agency costs.

According to the optimal contract theory, equity incentives are a way to effectively mitigate agency conflicts between shareholders and management. Therefore, it is necessary to implement equity incentives for executives to reduce the rent-seeking behavior of these executives. Some scholars believe that executives’ ownership of equity can force the managers and owners of a company to share the same goals [34], thus enhancing the managers’ sense of innovation and reducing the agency costs of the company [35, 36]. The intrinsic motivation of the management is crucial for enhancing corporate innovation, and the purpose of implementing equity incentives for executives is to align the interests of executives with those of shareholders, generate a convergence of interests, and stimulate managers’ innovation initiatives [37]. According to the theory of residual claims, equity incentives guarantee managers the right to share in the firm’s residual claims, encourage managers to focus on the firm’s long-term development, and favor the firm’s technological innovation activities. Managers establish the firm’s strategic decisions and thus determine and influence the allocation of resources for technological innovation. Equity incentives will encourage managers to allocate more resources to innovation projects and maintain the firm’s ability to carry out continuous innovation activities [38].

On the one hand, equity restriction will affect equity incentives, and on the other hand, equity incentives will affect innovation quality. Equity restriction promotes managers’ equity incentives, reduces agency costs, mitigates agency conflicts between shareholders and management, stimulates managers’ innovation initiatives, and encourages managers to increase innovation projects to achieve the company’s long-term development. As a result, innovation quality is improved. This study proposes the following hypothesis in light of the above analysis:

Hypothesis 2 (H2): Equity incentives in manufacturing companies play a mediating role between equity restriction and innovation quality.

### 2.3 Mediating effect of R&D investment

Equity restriction can promote increased R&D investment and reduce the agency costs associated with R&D, while companies with low equity restriction may require higher R&D costs, which may reduce R&D investment [16]. Companies with high equity restriction can avoid the arbitrariness of the first largest shareholder in innovative decision-making, mitigate conflicts of interest among shareholders, and unite shareholders to consider the long-term interests of the company, leading to a more positive attitude towards R&D investment and promoting increased R&D investment [39]. For example, Di Vito et al. [40] studied the Canadian manufacturing industry and confirmed that increased shareholding proportion for the first largest shareholder had a negative impact on R&D investment while equity restriction had a positive effect on promoting R&D investment.

R&D investment plays a decisive role among the various factors that affect innovation quality [41]. Innovation investment has a positive effect on innovation quality, especially on the number of invention patents that largely represent innovation quality. For instance, Hausman and Hall B [42] collected data from listed companies in the United States and proved that innovation investment and invention patent output are positively correlated. As technology has spillover effects, independent R&D of companies can effectively enhance technology absorption capacity at the industry level [43]. By increasing R&D investment, companies can absorb more specialized R&D personnel and maintain their industrial competitive advantages. In addition, companies can have more funds to tackle cutting-edge technologies, making it easier for them to produce high-quality innovation outputs.

On the one hand, equity restriction will have an impact on R&D investment, and on the other hand, R&D investment will have an impact on innovation quality. Equity restriction can effectively restrain the speculative behavior of major shareholders, reduce agency costs related to R&D, and enable major shareholders to invest more in R&D to tackle cutting-edge technologies in the long-term interests of the company. The increase in R&D investment will promote the output of high-quality innovative results, thereby promoting the improvement of innovation quality [44]. This study proposes the following hypothesis in light of the above analysis:

Hypothesis 3 (H3): R&D investment in manufacturing companies plays a mediating role between equity restriction and innovation quality.

**Fig 3:**
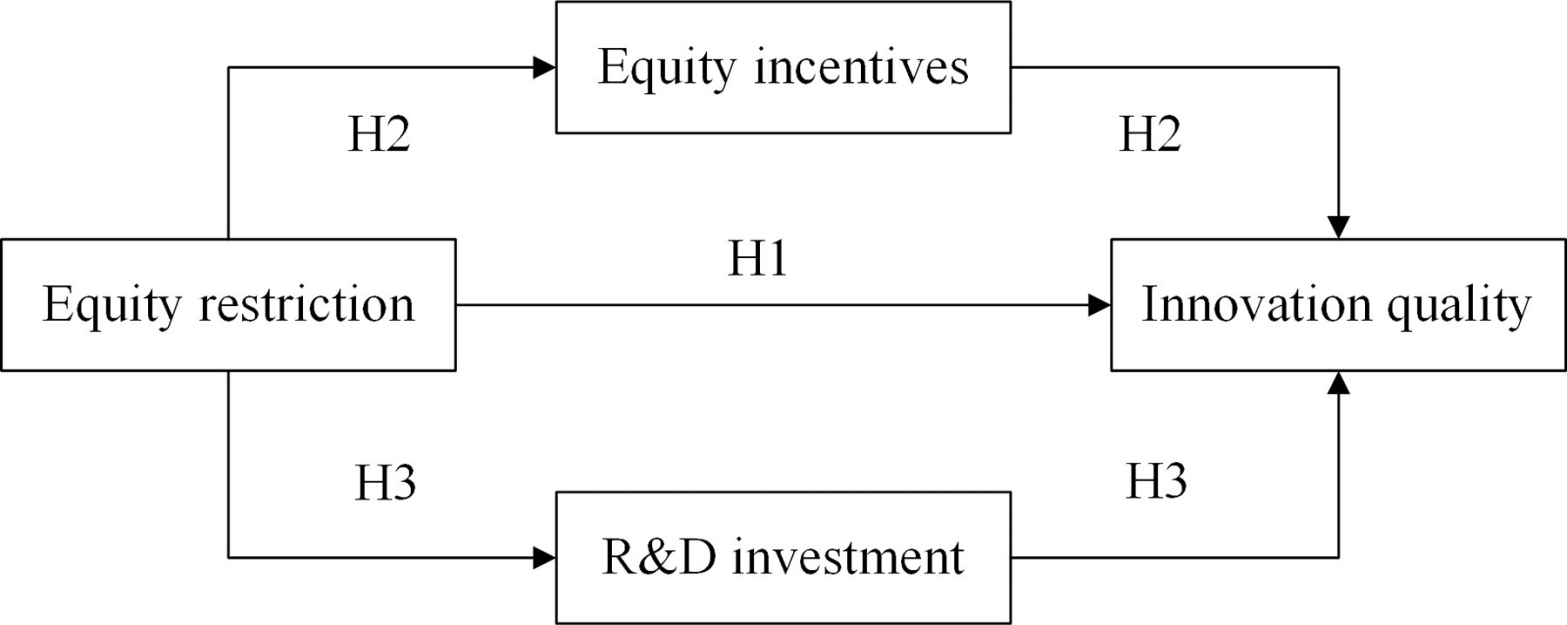
Hypothesized model.

The overall research model is shown in Fig 3.

## 3. Research design

### 3.1 Sample selection and data sources

This paper explores the impact of equity restriction on innovation quality, using the research sample of all A-share listed manufacturing companies in China from 2007 to 2021 [45]. The data from 2007 were collected considering the new accounting standards implemented in China since 2007, which made accounting information more comparable. Similar to previous studies, this paper screened the original data in the following order: firstly, samples with unidentified industry types were excluded; secondly, samples of companies that had been delisted due to special treatment (ST) were excluded; thirdly, samples of companies with serious missing values in the main variables were excluded. After data processing, 24,749 valid observations were obtained as the basic data for this study. The basic information and financial data of the above companies were sourced from the CSMAR database (Chinese Research Data Services). The patent application and authorization data were sourced from the CNRDS database (Chinese Research Data Services). Stata 17 software was used for data processing and analysis.

### 3.2 Model setting

The Tobit regression model, which is proposed by [46], describes the association between non-negative explained variable and explanatory variable when the data is truncated. The explained variable, namely innovation quality, is non-negative "truncated data" with a lower limit of 0 [47]. As the explained variable is limited, ordinary least square (OLS) would give inconsistent and biased parameter estimates. The Tobit regression model, one of the non-negative explained variable models, is able to handle the truncated explained variable data effectively. Therefore, to study how equity restriction affects the innovation quality, it is advisable to adopt the Tobit regression model for further investigations. The application of Tobit regression method can be found in the literature [48–51]. It is necessary to note that to keep the accuracy of results as much as possible, we employed a two-way fixed effect model because it is capable of controlling both unobserved industry-specific and year-specific confounders. The model is expressed as:

$${\mathrm{Patent}}_{i,t+2}{=\beta }_{0}{+\beta }_{1}{\mathrm{Balance}1}_{\mathrm{i,t}}{+\beta }_{2}{\mathrm{Controls}}_{\mathrm{i,t}}+\sum \mathrm{Year}+\sum \mathrm{Ind}{+\varepsilon }_{\mathrm{i,t}}$$ (1)

where Patent is the explained variable, representing innovation quality; β_0_ is the constant term; Balance1 is the explanatory variable representing equity restriction; β_1_ represents the regression coefficient of equity restriction; controls are control variables; i is the individual; t is the year; ∑Year and ∑Ind represent industry fixed effects and time fixed effects;ε_i,t_ is the random disturbance term. If β_1_ is significantly greater than 0, then equity restriction has a significant positive impact on innovation quality, and if β_1_ is significantly less than 0, then equity restriction has a significant negative impact on innovation quality.

### 3.3 Variable definition

#### 3.3.1 Explained variable: Innovation quality

Innovation quality is a comprehensive evaluation of the contribution and practical value of innovative technology [17]. Currently, the academic community generally measures an enterprise’s innovation quality by patent quality [1]. Some scholars measure patent quality by the number of patent citations [52], but Bessen [53] points out that this approach can only explain some of the differences in patent value and thus has certain limitations as a measure of patent quality. In addition, some scholars have used the number of invention patents to measure innovation quality [54]. According to the "Patent Law of the People’s Republic of China" [55], three types of Chinese patents may be distinguished, namely invention patents, utility model patents, and design patents. Compared to utility model and design patents, invention patents have a higher technological content and innovation value [56]. Therefore, this paper uses the number of granted invention patents as a proxy indicator of innovation quality. Considering the lagged effects of other variables on the number of granted invention patents, it usually takes about two years for a Chinese invention patent to be granted from the application date. Therefore, the explained variable is set two periods ahead, and the natural logarithm of the number of granted invention patents plus one (Patent) in the t+2 year is used to measure innovation quality. The natural logarithm of the number of invention patent applications plus one (Apply) in the t+2 year and the natural logarithm of the number of invention patents cited plus one (Cite) in the t+2 year are used as alternative indicators for innovation quality in the robustness test.

#### 3.3.2 Explanatory variable: Equity restriction

Equity restriction is an important component of corporate governance structure, which reflects the degree of restriction that the other major shareholders have on the first major shareholder. It is mainly represented by the sum of the shareholding percentages of the second to fifth largest shareholders divided by the shareholding percentage of the first largest shareholder in most literature [8, 15], and it is also used in this paper to measure equity restriction, referred to as "Balance1". In the robustness test, the sum of the shareholding percentages of the second to tenth largest shareholders divided by the shareholding percentage of the first largest shareholder (Balance2) and the sum of shareholding percentages of the second and third largest shareholders divided by the shareholding percentage of the first largest shareholder (Balance3) are used as alternative indicators for equity restriction.

#### 3.3.3 Control variable

To ensure the reliability of the relationship between the explanatory and explained variables, we introduced several other factors that may affect innovation quality as control variables in the model [57–60]. The control variables used in this paper include company age (Age), company size (Size), leverage ratio (Lev), fixed asset ratio (Fix), cash flows ratio (Cash), return on assets (Rov), and company growth (Growth). The definitions of the main variables are listed in Table 1.

**Table 1:** Variable definition.

Variable Type	Variable Symbol	Variable Name	Variable Definition
Explained variable	Patent	Innovation quality	Ln(The number of granted invention patents+1)
Explanatory variable	Balance1	Equity restriction	The sum of the shareholding percentages of the second to fifth largest shareholders/ the shareholding percentage of the first largest shareholder
Control variables	Age	Company age	Year of handling–year of corporate establishment
	Size	Company size	Ln(Total assets)
	Lev	Leverage ratio	Total liabilities/total assets
	Fix Cash Rov Growth	Fixed asset ratio Cash flows ratio Return on assets Company growth	Fixed asset/total assets Net cash flows/total assets Total profits/total assets The total assets growth rate

## 4. Results

### 4.1 Descriptive statistics

Table 2 presents the results of the descriptive statistics for the main variables, with a total of 24,749 company samples. Among them, 24,464 companies were granted invention patents, accounting for 98.85% of the total sample, indicating that the vast majority of A-share listed manufacturing companies in China had been authorized invention patents. The maximum and minimum values of innovation quality were 8.034 and 0 respectively, with a standard deviation of 1.085, indicating a large variation in innovation quality among companies. The median of innovation quality was 0.693, lower than the mean (0.822), indicating that the innovation quality of most companies was lower than the mean of the sample companies. The mean of equity restriction was 0.751, less than 1, indicating that the sum of the shareholding percentages of the second to the fifth largest shareholders was generally lower than that of the first largest shareholder. The situation of "one share dominates" is common in the listed manufacturing companies in China, but other shareholders still have a strong balancing effect on the controlling shareholder.

**Table 2:** Descriptive statistics.

Variable	N	Mean	p50	SD	Min	Max
Patent	24464	0.822	0.693	1.085	0	8.034
Balance1	24749	0.751	0.593	0.620	0.00400	4
Age	24749	16.76	16.42	5.980	0.920	63
Size	24749	21.89	21.74	1.212	16.41	27.55
Lev	24749	0.437	0.391	1.527	0.00700	178.3
Fix	24749	0.229	0.201	0.142	0	0.902
Cash	24749	0.190	0.151	0.140	0	0.978
Rov	24749	0.0330	0.0420	0.490	-48.32	10.40
Growth	24749	0.232	0.0790	2.868	-0.981	288.0

### 4.2 Correlation analysis

The correlation analysis was performed on the main variables, as shown in Table 3. According to Table 3, in manufacturing companies, equity restriction was significantly and positively correlated with innovation quality, which preliminarily supports the hypothesis that "equity restriction has a significant positive impact on innovation quality in manufacturing companies." In terms of controlling variables, company size, cash flows ratio, and return on assets were all positively correlated with innovation quality. In addition, company age, leverage ratio, fixed asset ratio, and company growth were negatively correlated. The correlation coefficients between variables were all less than 0.5, indicating that there was no multicollinearity problem in this model. However, correlation analysis can only preliminarily test the impact of equity restriction on innovation quality. To obtain more accurate and reliable conclusions, regression analysis is needed.

**Table 3:** Correlation analysis.

	Patent	Balance1	Age	Size	Lev	Fix	Cash	Rov	Growth
Patent	1								
Balance1	0.024***	1							
Age	-0.024***	0.011*	1						
Size	0.283***	-0.092***	0.187***	1					
Lev	-0.012*	-0.030***	0.023***	-0.025***	1				
Fix	-0.068***	-0.101***	-0.00100	0.113***	0.038***	1			
Cash	0.050***	0.062***	-0.138***	-0.176***	-0.071***	-0.385***	1		
Rov	0.023***	0.00800	-0.023***	0.055***	-0.599***	-0.027***	0.042***	1	
Growth	-0.00500	0.00900	0.00600	0.039***	-0.00100	-0.0100	-0.00500	0.015**	1

Note: *, **, *** are significant at the 10%, 5%, and 1% levels, respectively.

### 4.3 Regression analysis

Table 4 displays the regression results for model (1). In column (1), no control variables were added, and the regression coefficient of equity restriction on innovation quality was 0.113, significant at the level of 1%. In column (2), control variables were added, and the regression coefficient of equity restriction was 0.171, still significant at the level of 1%. The results showed that whether or not control variables should be added, the regression coefficient of equity restriction on innovation quality was significantly positively correlated at the level of 1%, indicating that equity restriction had a significant positive impact on innovation quality, thereby proving Hypothesis 1 proposed in this paper, which was potential because equity restriction could prevent the first largest shareholder from abusing their controlling power to infringe on the interests of minority shareholders and that mutual supervision between large shareholders was beneficial for making more scientific innovation investment decisions conducive to the long-term development of the company, thereby promoting the improvement of innovation quality [26].

**Table 4:** Regression results of equity restriction and innovation quality.

Variable	(1)	(2)
	Patent	Patent
Balance1	0.113***	0.171***
	(4.59)	(7.29)
Age		-0.032***
		(-12.08)
Size		0.459***
		(36.75)
Lev		0.002
		(0.14)
Fix		-0.895***
		(-8.06)
Cash		1.017***
		(8.94)
Rov		0.537***
		(5.03)
Growth		-0.012**
		(-2.42)
Constant	0.056**	-9.481***
	(2.25)	(-34.20)
Industry	Control	Control
Year	Control	Control
Observations	19,087	19,087
Pseudo R^2^	0.000389	0.0282

Note: *, **, *** are significant at the 10%, 5%, and 1% levels, respectively.

The regression results for the control variables in column (2) show that there was a significant positive correlation between company size and innovation quality, indicating that the larger the company size, the more favorable it was for improving innovation quality. Cash flows ratio was also significantly positively correlated with innovation quality, indicating that the more liquid cash a company held, the more positive impact it had on innovation quality. Return on assets was also significantly positively correlated with innovation quality, indicating that as the return on assets increased, the innovation quality of the company continued to be significantly improved. Company age, fixed asset ratio, and company growth were all significantly negatively correlated with innovation quality. The coefficient between the leverage ratio and innovation quality was positive, but not significant.

### 4.4 Robustness test

To ensure the robustness of the regression analysis results, we conducted robustness checks by changing the measurement method of equity restriction and innovation quality, the timing of innovation quality measurement, and the regression model.

#### 4.4.1 Change the measurement method of equity restriction

According to Xin Long Xu [15], La Porta [61], Laeven [62], Lin [63], and BenNasr [64], we measured the equity restriction with two alternative variables. One was the sum of shareholding percentages of the second to the tenth largest shareholders divided by the shareholding percentage of the first largest shareholder (Balance2), and the other was the sum of shareholding percentages of the second and third largest shareholders divided by the shareholding percentage of the first largest shareholder (Balance3). The regression results are displayed in columns (1) and (2) in Table 5. It can be seen from the table that the regression coefficients for both balance 2 and balance 3 were significantly positive at the level of 1%, showing that the conclusion that equity restriction had a significant positive impact on innovation quality remained robust under the alternative measure of equity restriction.

**Table 5:** Robustness test results.

Variable	(1)	(2)	(3)	(4)	(5)	(6)
	Patent	Patent	Apply	Cite	Patent	Patent
Balance1			0.172***	0.106***	0.170***	0.087***
			(6.89)	(0.31)	(6.61)	(6.58)
Balance2	0.142***					
	(8.15)					
Balance3		0.108***				
		(2.98)				
Age	-0.032***	-0.043***	-0.038***	-0.026***	-0.038***	-0.014***
	(-12.08)	(-14.51)	(-13.72)	(-11.27)	(-13.12)	(-9.86)
Size	0.459***	0.475***	0.450***	0.521***	0.483***	0.301***
	(36.79)	(31.03)	(33.88)	(47.28)	(35.44)	(30.86)
Lev	0.003	-0.557***	0.010	0.020	-0.017	0.012***
	(0.16)	(-5.88)	(0.60)	(0.65)	(-0.67)	(2.95)
Fix	-0.875***	-0.594***	-0.851***	-0.914***	-0.794***	-0.571***
	(-7.88)	(-4.77)	(-7.25)	(-9.50)	(-6.61)	(-9.91)
Cash	1.017***	0.843***	1.183***	0.390***	1.189***	0.546***
	(8.95)	(6.28)	(9.79)	(3.95)	(9.70)	(9.25)
Rov	0.538***	0.479***	0.853***	0.286***	0.504***	0.015
	(5.05)	(4.07)	(7.56)	(2.68)	(4.41)	(1.47)
Growth	-0.012**	-0.009*	-0.016***	-0.008*	-0.010**	-0.006*
	(-2.43)	(-1.74)	(-2.93)	(-1.82)	(-2.08)	(-1.81)
Constant	-9.493***	-9.407***	-8.696***	-8.831***	-9.959***	-5.524***
	(-34.32)	(-28.48)	(-29.54)	(-36.58)	(-32.90)	(-25.95)
Industry	Control	Control	Control	Control	Control	Control
Year	Control	Control	Control	Control	Control	Control
Observations	19,087	19,087	19,114	14,411	16,763	19,087
Pseudo R^2^	0.0284	0.107	0.0228	0.0424	0.0301	0.102

Note: *, **, *** are significant at the 10%, 5%, and 1% levels, respectively.

#### 4.4.2 Change the measurement method of innovation quality

Referring to Tan et al. [54] and Trajtenberg M [65], we used the following two alternative indicators to measure innovation quality respectively. One was the natural logarithm of the number of invention patent applications plus one (Apply) in the t+2 year, and the other was the natural logarithm of the number of invention patents cited plus one (Cite) in the t+2 year. The regression results in columns (3) and (4) in Table 5 demonstrate that when Apply or Cite was used as the explained variable, the regression coefficient of equity restriction (Balance1) was significantly positive at the level of 1%, which suggests that the conclusion that equity restriction had a significant positive impact on innovation quality remained robust when Apply or Cite was utilized as an alternative indicator for innovation quality.

#### 4.4.3 Change the timing of innovation quality measurement

In essence, innovation activity is a long-term investment for companies, and it usually takes a long cycle to transform innovation inputs into high-quality innovation outputs in the future. To examine whether the research conclusion was sensitive to the timing of measuring innovation quality, we reset the timing of innovation quality measurement to t+3 year for regression analysis according to Model (1). The findings are displayed in column (5) of Table 5, with a regression coefficient of 0.170 for Balance1, significant at the level of 1%, which was consistent with the above conclusions.

#### 4.4.4 Change the regression model

According to the distribution characteristics of patent data—truncated data, the above studies all used the Tobit model for regression analysis. To examine the sensitivity of the research conclusions to the model setting, an ordinary least squares (OLS) model was used to test the robustness of the model [66, 67]. Column (6) of Table 5 displays the regression findings. After the regression model was replaced with the OLS model, the regression coefficient of equity restriction (Balance1) was 0.087, significant at the level of 1%, which was consistent with the above results.

### 4.5 Endogenous test

There may be endogeneity issues between equity restriction and innovation quality due to selection bias. On the one hand, equity restriction promotes the improvement of innovation quality, and the long-term profitability demonstrated by high-quality innovation will attract more shareholders to invest, thereby increasing equity restriction, which potentially results in self-selection bias. On the other hand, processing missing patent data is likely to cause bias in sample selection. The Heckman two-stage model, which is proposed by [68], is mainly suitable for endogeneity problems caused by self-selection bias and sample selection bias [69]. The model is divided into two stages: the first stage is a probabilistic model for estimating the likelihood of the existence of a selection bias variable, which allows the inverse Mills ratio (IMR) to be derived. The inverse Mills ratio is then added to the second stage model and regressed along with the other variables to correct for the endogeneity problem. Therefore, we control for endogeneity caused by selection bias with the Heckman two-stage model [70]. Firstly, we selected appropriate instrumental variables to include in a first-stage probability model. Following Paligorova and Xu [71], BenNasr [64], and Zhang et al. [72], we use the average equity restriction (IV_balance1) in the city where the enterprise was located in the previous year as an exogenous instrumental variable. As the equity restriction of a single enterprise is usually related to the average equity restriction in the city (industrial cluster) where it is located in the previous year, the change in the innovation quality of the enterprise cannot affect the average equity restriction in the entire city in the previous year. A dummy variable indicating whether patent data is missing was taken as the explained variable in the first-stage model, and an exogenous instrumental variable (IV_balance1) was added to calculate the inverse Mills ratio (IMR). Secondly, the calculated IMR from the first stage was used in the second-stage model. As shown in the second-stage regression results in column (2) in Table 6, the regression coefficient of IMR was not significant, indicating the absence of significant bias in self-selection or sample selection. Moreover, the regression coefficient of equity restriction (Balance1) on innovation quality (Patent) was 0.077 and significant at the level of 1%, demonstrating that the conclusion that equity restriction had a significant positive impact on innovation quality remained robust when endogeneity issues were taken into account.

**Table 6:** Regression results of Heckman two-stage model.

Variable	(1)	(2)
	First stage	Second stage
	Patent	
Balance1		0.077***
		(5.33)
Age	-0.068***	-0.018***
	(-41.23)	(-6.15)
Size	-0.079***	0.297***
	(-9.96)	(41.82)
Lev	-0.008	0.011
	(-1.08)	(1.33)
Fix	1.025***	-0.520***
	(13.69)	(-7.78)
Rov	-0.033	0.014
	(-0.98)	(0.76)
Cash	-0.224***	0.534***
	(-3.03)	(8.65)
Growth	0.014*	-0.006**
	(1.70)	(-2.46)
IV_balance1	-0.798***	
	(-20.49)	
IMR		0.129
		(1.57)
Constant	4.165***	-5.425***
	(23.27)	(-33.77)
Industry	Control	Control
Year	Control	Control
Observations	24,749	24,749

Note: *, **, *** are significant at the 10%, 5%, and 1% levels, respectively.

### 4.6 Mediating effect analysis

Baron and Kenny [73] proposed a mediating effects model to account for the underlying mechanism of the relationship between the explanatory and explained variables by introducing mediating variables. On this basis, to further explore the relationship between equity restriction and innovation quality, the three-step regression method of the mediation effect was adopted in this paper [74]. Models (2) and (3) were constructed using two mediating variables, equity incentives [31] and R&D investment [41], to test hypotheses 2 and 3. Equity incentives and R&D investment were used to validate the intrinsic mechanism by which equity restriction promoted the improvement of innovation quality.

$${\mathrm{Patent}}_{\mathrm{i,t}+2}{=\beta }_{0}{+\beta }_{1}{\mathrm{Balance}1}_{\mathrm{i,t}}{+\beta }_{2}{\mathrm{Controls}}_{\mathrm{i,t}}+\sum \mathrm{Year}+\sum \mathrm{Ind}+\varepsilon_{\mathrm{i,t}},$$

$${\mathrm{Shainc}}_{\mathrm{i,t}}{=\gamma }_{0}{+\gamma }_{1}{\mathrm{Balance}1}_{\mathrm{i,t}}{+\gamma }_{2}{\mathrm{Controls}}_{\mathrm{i,t}}+\sum \mathrm{Year}+\sum \mathrm{Ind}+\varepsilon_{\mathrm{i,t}},$$

$${\mathrm{Patent}}_{\mathrm{i,t}+2}{=\delta }_{0}{+\delta }_{1}{\mathrm{Balance}1}_{\mathrm{i,t}}{+\delta }_{2}{\mathrm{Shainc}}_{\mathrm{i,t}}{+\delta }_{3}{\mathrm{Controls}}_{\mathrm{i,t}}+\sum \mathrm{Year}+\sum \mathrm{Ind}+\varepsilon_{\mathrm{i,t}}.$$ (2)

$${\mathrm{Patent}}_{\mathrm{i,t}+2}{=\beta }_{0}{+\beta }_{1}{\mathrm{Balance}1}_{\mathrm{i,t}}{+\beta }_{2}{\mathrm{Controls}}_{\mathrm{i,t}}+\sum \mathrm{Year}+\sum \mathrm{Ind}+\varepsilon_{\mathrm{i,t}},$$

$${\mathrm{RD}}_{\mathrm{i,t}}{=\gamma }_{0}{+\gamma }_{1}{\mathrm{Balance}1}_{\mathrm{i,t}}{+\gamma }_{2}{\mathrm{Controls}}_{\mathrm{i,t}}+\sum \mathrm{Year}+\sum \mathrm{Ind}+\varepsilon_{\mathrm{i,t}},$$

$${\mathrm{Patent}}_{\mathrm{i,t}+2}{=\delta }_{0}{+\delta }_{1}{\mathrm{Balance}1}_{\mathrm{i,t}}{+\delta }_{2}{\mathrm{RD}}_{\mathrm{i,t}}{+\delta }_{3}{\mathrm{Controls}}_{\mathrm{i,t}}+\sum \mathrm{Year}+\sum \mathrm{Ind}+\varepsilon_{\mathrm{i,t}}.$$ (3)

where Shainc and RD are the mediating variables, of which Shainc represents equity incentives, expressed as the ratio of the sum of executives’ shareholdings to the total shares of the company, and RD represents R&D investment, expressed as the ratio of R&D expenses to operating income. The meanings of other variables are the same as above. If the regression coefficient (γ_1_) of equity restriction (Balance1) on the mediating variable is significant and the regression coefficient (δ_2_) of the mediating variable on innovation quality (Patent) is also significant, then the mediating variable plays a mediating role. Meanwhile, if δ_1_ is significant, it is partially mediated, and if δ_1_ is insignificant, it is fully mediated.

### 4.6.1 Equity incentives

The mediation effect path of "equity restriction → equity incentives → innovation quality" and Model (2) were utilized to analyze the mediating role of equity incentives between equity restriction (Balance1) and innovation quality (Patent). The results in column (1) of Table 7 show that without the mediating variable, equity restriction and innovation quality were significantly positively correlated at the level of 1%, indicating the existence of a basis for testing the mediation effect. Column (2) shows that the regression coefficient of equity restriction on equity incentives was 8.463, significant at the level of 1%, indicating a significant positive influence of equity restriction on equity incentives. Column (3) simultaneously includes equity restriction and equity incentives in the regression equation. The results show that the regression coefficient of equity incentives on innovation quality was significantly positive at the level of 1%, which means that equity incentives helped improve innovation quality. The correlation coefficient of equity restriction on innovation quality decreased from 0.171 in column (1) to 0.099, but the significant positive influence of equity restriction on innovation quality remained unchanged. The above analysis shows that equity incentives played a partial mediating role between equity restriction and innovation quality, thus validating hypothesis H2. Equity restriction optimized corporate governance structure, promoted equity incentives for managers, reduced agency costs, and ultimately improved innovation quality [75].

**Table 7:** Mediating effect of equity incentives.

Variable	(1)	(2)	(3)
	Patent	Shainc	Patent
Balance1	0.171***	8.463***	0.099***
	(7.29)	(32.20)	(4.17)
Shainc			0.012***
			(14.53)
Age	-0.032***	-0.457***	-0.028***
	(-12.08)	(-16.17)	(-10.54)
Size	0.459***	-4.624***	0.504***
	(36.75)	(-31.78)	(39.11)
Lev	0.002	-0.323**	0.012
	(0.14)	(-2.07)	(0.70)
Fix	-0.895***	-25.729***	-0.710***
	(-8.06)	(-19.73)	(-6.38)
Cash	1.017***	15.935***	0.846***
	(8.94)	(12.54)	(7.43)
Rov	0.537***	3.161***	0.490***
	(5.03)	(3.31)	(4.56)
Growth	-0.012**	-0.075	-0.012**
	(-2.42)	(-1.15)	(-2.37)
Constant	-9.481***	109.093***	-10.631***
	(-34.20)	(33.97)	(-36.79)
Industry	Control	Control	Control
Year	Control	Control	Control
Observations	19,087	24,749	19,087
Pseudo R^2^	0.0282	0.0263	0.0321

Note: *, **, *** are significant at the 10%, 5%, and 1% levels, respectively.

#### 4.6.2 R&D investment

The mediating effect of R&D investment between equity restriction and innovation quality was analyzed based on the path of "equity restriction → R&D investment → innovation quality" using Model (3). As shown in the results in Table 8’s column (2), the regression coefficient of equity restriction on R&D investment was 0.155, significant at the level of 1%, showing that equity restriction could effectively significantly promote R&D investment. Both equity restriction and R&D investment were added to the regression equation in column (3), and the results showed that the R&D investment regression coefficient on innovation quality was significantly positive at the level of 1%, which suggests that R&D investment promoted the improvement of innovation quality. Furthermore, the correlation coefficient of equity restriction on innovation quality decreased from 0.171 in column (1) to 0.097, but there was still a significant positive correlation between the two. The analysis above demonstrates that R&D investment played a partial mediating role between equity restriction and innovation quality, thus validating hypothesis H3. Equity restriction could effectively discourage speculation by the largest shareholder, reduce agency costs associated with R&D, promote an increase in R&D investment, and ultimately improve innovation quality [44].

**Table 8:** Mediating effect of R&D investment.

Variable	(1)	(2)	(3)
	Patent	RD	Patent
Balance1	0.171***	0.155***	0.097***
	(7.29)	(6.30)	(4.24)
RD			7.778***
			(28.50)
Age	-0.032***	0.022***	-0.034***
	(-12.08)	(8.47)	(-13.45)
Size	0.459***	0.133***	0.481***
	(36.75)	(9.42)	(39.30)
Lev	0.002	-1.236***	0.037**
	(0.14)	(-15.93)	(2.28)
Fix	-0.895***	-1.070***	-0.426***
	(-8.06)	(-8.92)	(-3.88)
Cash	1.017***	-0.320***	0.889***
	(8.94)	(-2.59)	(7.99)
Rov	0.537***	-1.120***	0.930***
	(5.03)	(-12.48)	(9.01)
Growth	-0.012**	-0.003	-0.011**
	(-2.42)	(-0.48)	(-2.22)
Constant	-9.481***	-2.873***	-10.257***
	(-34.20)	(-9.56)	(-37.56)
Industry	Control	Control	Control
Year	Control	Control	Control
Observations	19,087	24,749	19,087
Pseudo R^2^	0.0282	0.00590	0.0430

Note: *, **, *** are significant at the 10%, 5%, and 1% levels, respectively.

## 5. Discussion

The results obtained are mainly discussed in this section. Firstly, the results showed that equity restriction had a significant positive impact on innovation quality in manufacturing companies. Existing research, such as that conducted by Xin Long Xu et al. [15], has demonstrated the promoting effect of equity restriction on innovation by using innovation inputs as a measure of innovation. However, this study expanded the explained variable to "innovation quality" and measured it using the number of invention patents, mainly because invention patents represent higher technological content and innovation value in China [56]. All A-share listed companies in China’s manufacturing industry were used as the research sample, which enriched existing literature on innovation quality in manufacturing companies. The above research conclusion may be due to the fact that from the perspective of principal-agent theory, equity restriction in manufacturing companies restricts the behavior of the largest shareholder from using control rights for private benefits, reduces the squeeze on innovation resources, and equity restriction can promote an increase in innovation investment; increased innovation investment can promote the output of high-quality innovation results, thereby promoting the improvement of corporate innovation quality [76].

Secondly, the research results suggested that equity restriction promoted innovation quality through equity incentives. In the existing literature, Jensen and Meckling [31] propose that as the level of equity restriction increases, the board of directors is more inclined to implement management equity holdings and equity incentives for management; Wu et al. [77] believe that shareholder equity incentive enables managers to consider the long-term performance of enterprises more in daily operation and increase R&D investment, thus promoting the improvement of innovation quality. In contrast, this study further examined equity incentives as a mediating variable and analyzed its mediating effect between equity restriction and innovation quality. A mechanism analysis based on the intermediate effect path of "equity restriction → equity incentives → innovation quality" is proposed in this paper, extending the existing theoretical analysis. According to principal-agent theory, as the level of equity restriction increases, shareholders are more inclined to implement equity incentives to solve the agency problem between managers and shareholders, which can reduce agency costs and ensure consistency in the long-term development goals of managers and shareholders. When managers’ roles change from operators to shareholders, they will make more scientific decisions on innovation investment based on the long-term interests of the enterprise, thus improving innovation quality [78].

Thirdly, the research results also showed that R&D investment played a partial mediating role between equity restriction and innovation quality. Previous studies, such as that performed by Volpin [39], have found that equity restriction can help to diversify the risks of investing in innovation projects and promote an increase in R&D investment. Scholars such as Artz [79] and Bogner [80] conducted a series of empirical studies on the connection between R&D investment and innovation quality, all of which show a significant positive correlation between the two. However, this study further used R&D investment as a mediating variable to explore its role in mediating the relationship between equity restriction and innovation quality. A mechanism analysis based on the intermediate effect path of "equity restriction → R&D investment → innovation quality" was proposed in this paper to expand the content of existing theoretical analysis. The above research result may be attributed to the fact that, based on principal-agent theory, equity restriction can alleviate conflicts of interest among shareholders, enabling them to join forces and adopt a more positive attitude toward R&D investment for the long-term benefit of the company. Equity restriction has a significant positive impact on R&D investment, and as R&D investment increases, it can promote the production of high-quality innovative outcomes, thereby enhancing the innovation quality of the enterprise [44].

## 6. Conclusions and recommendations

### 6.1 Conclusions

This paper empirically analyzed the relationship between equity restriction and innovation quality, and verified the mediating role of equity incentives and R&D investment in this relationship. In this study, we take all A-share listed manufacturing companies in China from 2007 to 2021 as the research sample and use the number of invention patents to measure innovation quality. We arrive at the following conclusions: First, equity restriction has a significant positive impact on innovation quality in manufacturing companies, which is also confirmed by the robustness test and endogenous test using Heckman two-stage model. Secondly, the intrinsic impact mechanism of equity restriction on innovation quality was explored. The results show equity incentives and R&D investment play a partial mediating role between equity restriction and innovation quality and that equity restriction can enhance the innovation quality of manufacturing companies through these two paths.

As an important mechanism in corporate governance structure, the relationship between equity restriction and innovation has always been a very important research field. However, there is little research on how equity restriction affects innovation quality. The research findings of this paper provide new evidence for the relationship between equity restriction and innovation quality, enrich the research content of the influencing factors of innovation quality in Chinese manufacturing companies, and provide a theoretical basis for other developing countries’ manufacturing industries to shift towards high-quality innovation based on equity restriction. In addition, there are few studies in the existing literature on the intrinsic impact mechanism of equity restriction on innovation quality. This paper employed equity incentives and R&D investment as mediating variables to conduct mediation effect tests, which deepens the analysis of the mechanism of the role of equity restriction on innovation quality and further expands the content of existing theoretical analysis. At the same time, in the context of China’s innovation-driven development, the research findings of this paper have certain guiding and enlightening significance for manufacturing companies to improve their corporate governance structure, give full play to the role of equity restriction, and shift towards high-quality innovation [81].

### 6.2 Recommendations

Manufacturing companies should increase the level of equity restriction to give full play to the monitoring and restraining role of the monitoring shareholders over the first largest shareholder. The monitoring shareholders can also restrain the first largest shareholder’s interest encroachment behavior by monitoring the board of directors and the managerial layer, thus promoting the improvement of the quality of corporate innovation. The research findings in this paper show that equity restriction has a significant positive impact on innovation quality. Therefore, to improve the innovation quality of Chinese manufacturing companies, it is very important to improve the level of equity restriction. As a socialist emerging market country, China’s manufacturing listed companies are characterized by concentrated equity ownership, making it necessary to attract the attention of the government and society to effectively improve the agency conflicts between shareholders under high equity concentration. The use of equity restriction can restrain the self-interest behavior of major shareholders, improve the company’s governance structure, and promote the improvement of enterprise innovation quality. Furthermore, improving innovation quality can create more value for shareholders and society.

In the process of improving innovation quality, manufacturing companies should establish an equity incentive system to give executives equity incentives. Enterprises should select appropriate equity incentive plans based on their own circumstances such as development scale, development stage, and development strategy; and focus on the scientific implementation of the management equity incentive system. In addition to improving the level of equity restriction and exerting its direct effects, companies should also be aware of the mediating role of equity incentives between equity restriction and innovation quality. Improving the equity incentive system for executives and providing them with equity incentives can enable them to benefit from the long-term development of the company while ensuring that executives make innovative decisions centered on maximizing the company’s value, which in turn leads to more high-quality innovation activities and promotes the improvement of innovation quality in the company.

The research conclusion shows that the positive impact of equity restriction on innovation quality can be achieved through the mediating variable of R&D investment. Therefore, increasing R&D investment has a positive promoting effect on innovation quality. Manufacturing companies are advised to increase their efforts in R&D investment, improve the management mechanism of R&D funds, and improve fund utilization efficiency to promote the improvement of innovation quality. In addition, the government and relevant departments should encourage and guide enterprises to increase R&D investment by formulating policies such as financial subsidies and tax exemptions. Companies can also further increase R&D investment by improving the level of equity restriction, thereby utilizing the mediating role of R&D investment between equity restriction and innovation quality and comprehensively promoting the improvement of innovation quality.

### 6.3 Limitations and prospects

This paper also has certain limitations stated as follows. Firstly, only Chinese A-share listed manufacturing companies are used as the research sample, which makes research conclusions more applicable in the field of manufacturing. Secondly, according to property rights, manufacturing companies can be divided into state-owned and non-state-owned companies. Under the socialist market economy system with Chinese characteristics, these two types of companies have significant differences in resource endowment, which may affect the research conclusions of this paper. Thirdly, the characteristics of the shareholders may have a different impact on the results of the study, for example, distinguishing between natural persons and organizations, and natural person shareholders can be categorized into different genders [82, 83]. Therefore, manufacturing companies can be grouped and assessed according to different property rights and shareholder characteristics in future research, and whether there are differences in the conclusions of different groups should also be verified.

## Supporting information

S1 Dataset The data set used in this article for discussion and analysis. (ZIP)
