# Supplementary material for: How does equity restriction affect innovation quality? Evidence from listed manufacturing companies in China
Source: PLoS One. 2023 Dec 7;18(12):e0295553. doi: 10.1371/journal.pone.0295553 (PMC10703261; doi:10.1371/journal.pone.0295553)
Supplement: S1 Dataset — (ZIP) [file pone.0295553.s001.zip › Supporting information/S1 Dataset/╔╧╩╨╣1⁄2╦╛╫¿└√╔Ω╟δ╟Θ┐÷-CNRDS╩2╛▌┐Γ/░μ╚¿╔∙├≈╙δ╩2╛▌└┤╘┤╦╡├≈.pdf]

版权声明

1. 除非中国研究数据服务平台(CNRDS)另行声明外，由中国研究数据服务平台(CNRDS)运营的软件产品的全部版本（包括已有版本及未来更新版本）及与该软件作品全部版本有关的产品、技术、软件、程序、数据及其他信息（包括文字、图标、图片、照片、音频、视频、图表、色彩组合、版面设计、商标、商号、域名等）的所有权利（包括但不限于著作权、商标权、专利权、商业秘密等相关权利）均属中国研究数据服务平台(CNRDS)所有。

2. 任何单位和个人或其他任何形式的法律实体未经中国研究数据服务平台(CNRDS)书面授权，不得以任何目的（包括但不限于学习、研究等非商业用途）修改、使用、复制、截取、编纂、编译、上传、下载等或以任何方式和媒介复制、转载和传播本软件作品的任何部分，否则将视为侵权。

3. 对于不遵守本声明和/或其他侵权违法行为，中国研究数据服务平台(CNRDS)保留依法追究其法律责任的权利。

数据来源说明

为发挥中国研究数据服务平台(CNRDS)软件产品所提供数据服务的使用价值，并确保所引用之中国研究数据服务平台(CNRDS)软件产品的正确性，以下对如何正确引用中国研究数据服务平台(CNRDS)软件产品作如下说明：

任何使用中国研究数据服务平台(CNRDS)软件产品的单位和个人，承诺只将中国研究数据服务平台(CNRDS)的数据用于学术研究，并在所得研究成果（包括但不限于学术论文、咨询报告、新闻稿件等）中必须注明数据来源于中国研究数据服务平台(CNRDS)。数据来源的说明请参考：“本研究数据来源于中国研究数据服务平台(CNRDS)” 或 “本研究数据来源于中国研究数据服务平台(CNRDS)XXX 数据库”; 英文参考：“We get the data from Chinese Research Data Services(CNRDS) Platform” 或 “We get the data from XXX database of Chinese Research Data Services(CNRDS) Platform”。
