# Supplementary material for: How does equity restriction affect innovation quality? Evidence from listed manufacturing companies in China
Source: PLoS One. 2023 Dec 7;18(12):e0295553. doi: 10.1371/journal.pone.0295553 (PMC10703261; doi:10.1371/journal.pone.0295553)
Supplement: S1 Dataset — (ZIP) [file pone.0295553.s001.zip › Supporting information/S1 Dataset/╔╧╩╨╣1⁄2╦╛╣·─┌═Γ╫¿└√╔Ω╟δ╗±╡├╟Θ┐÷▒φ/░μ╚¿╔∙├≈.pdf]

版权声明

1. 本版权声明是深圳希施玛数据科技有限公司关于"CSMAR Solution软件V4.4"软件产品的全部版本(包括已有版本及未来更新版本)及与该软件作品全部版本有关的源代码、目标代码、文档资料以及任何由深圳希施玛数据科技有限公司基于软件技术维护或支持服务所提供的数据库及查询方式、数据、资料等(以下统称:本软件作品)做出的法律声明。

2. 本软件作品的著作权、商标权等知识产权属于深圳希施玛数据科技有限公司所有,受《中华人民共和国著作权法》、《计算机软件保护条例》、《知识产权保护条例》和相关国际版权条约、法律、法规,以及其它知识产权法律和条约的保护。

3. 本软件包含的所有产品、技术、软件、程序、数据及其他信息(包括但不限于源代码、目标代码、文字、图像、图片、照片、音频、视频、图表、色彩、版面设计、电子文档)的所有权利(包括但不限于版权、商标权、专利权、商业秘密及其他所有相关权利),均归我司或有权第三方所有。

4. 任何单位和个人未经深圳希施玛数据科技有限公司书面授权,不得以任何目的(包括但不限于商业用途以及学习、研究等非商业用途)修改、使用、复制、截取、编纂、编译、上传、下载等或以任何方式和媒介复制、转载和传播本软件作品的任何部分,否则将视为侵权,深圳希施玛数据科技有限公司保留依法追究其法律责任的权利。

CSMAR
