# Supplementary material for: How does equity restriction affect innovation quality? Evidence from listed manufacturing companies in China
Source: PLoS One. 2023 Dec 7;18(12):e0295553. doi: 10.1371/journal.pone.0295553 (PMC10703261; doi:10.1371/journal.pone.0295553)
Supplement: S1 Dataset — (ZIP) [file pone.0295553.s001.zip › Supporting information/S1 Dataset/╩╨│í╗»╓╕╩2/1997-2022─Ω╩╨│í╗»╓╕╩2║1⁄4╖╓╧ε╓╕╩2ú¿╫ε╓╒╩2╛▌▓╬┐╝╬─╧╫│⌡╩╝╩2╛▌╧Ω╧╕┤a└φ┤·┬δú⌐/1997-2022─Ω╩╨│í╗»╓╕╩2║1⁄4╖╓╧ε╓╕╩2ú¿╫ε╓╒╩2╛▌+▓╬┐╝╬─╧╫+│⌡╩╝╩2╛▌+╧Ω╧╕┤a└φ┤·┬δú⌐/╞≤╥╡┬╠╔1⁄2┤┤╨┬╩╡╝∙╚τ║╬╞╞╜Γí░║═╨│╣▓╔·í▒─╤╠Γú┐_╜Γ╤o├╖.pdf]

企业绿色创新实践如何破解“和谐共生”难题？*

□解学梅　朱琪玮

摘要：在当前中国经济转型过程中，制造业企业亟须借助绿色创新突破制度和效率困境以实现可持续发展。本研究立足本土“和谐共生”文化，基于二元合法性理论，构建了一个条件过程效应模型，探讨制造业企业绿色创新影响其可持续发展绩效的内在机制与边界条件。基于2013~2018年我国重污染制造业上市公司数据，研究发现：(1)绿色工艺创新、绿色产品创新均能促进企业可持续发展绩效，但绿色工艺创新相比绿色产品创新更能改善企业环境社会责任绩效，而绿色产品创新相比绿色工艺创新更能提升企业财务绩效；(2)二元合法性(适应合法性、战略合法性)的中介作用揭示了绿色创新与企业可持续发展绩效关系中的和谐机制；(3)伦理型领导正向调节二元合法性在绿色工艺创新与可持续发展绩效之间的中介效应；但未能显著调节二元合法性在绿色产品创新与可持续发展绩效之间的中介效应。为此，本文进一步设计Post-hoc检验，结果表明：伦理型领导与绿色补贴的交互作用可以增强二元合法性在绿色产品创新和可持续发展绩效之间的中介效应。此外，经过一系列内生性与稳健性检验，上述结论依然成立。本研究不仅从细分维度丰富了绿色创新理论研究范畴，为绿色创新与二元合法性理论的融合研究提供了新的视角；而且为制造业企业的绿色创新实践提供了理论借鉴和指导。

关键词：绿色工艺创新　绿色产品创新　适应合法性　战略合法性　伦理型领导

DOI:10.19744/j.cnki.11-1235/f.2021.0009

一、引言

当前，各国企业在推动经济增长的同时正面临着环境变化带来的诸多挑战；由此，寻求企业与环境“和谐共生”的可持续发展战略途径成为全球性问题(李维安等，2019)。中国企业作为新兴市场的重要参与者，推行可持续发展战略不仅是遵循绿色经济发展的内在要求，更是积极应对环境挑战的责任担当。然而，长期以来，我国企业在推行可持续发展战略过程中面临的一个重大挑战是如何改善环境效益的同时创造新的经济增长点。目前，被政府和消费者等利益相关者广泛认可的观点是：绿色创新是实现企业可持续发展目标的重要战略(Huang and Li，2017)。例如，西门子、丰田等国际企业已通过绿色创新赢得了良好的环境社会责任“面子”和经济价值“里子”，成为“全球最佳可持续发展企业百强”榜单的常客。然而，作为新兴经济体普遍具有的典型特征，制度和市场不够完善使得我国企业绿色创新战略的实施面临一系列挑战。首先，外部资源的获取(例如，政府补贴)依赖于企业对政府制度的“遵从”；而创新资源的优化和配置要求企业发挥“能动性”(郭海等，2018)，由此产生“二元悖论”。其次，市场导向可以为企业实施绿色创新提供保障(El-Kassar and Singh，2019)；但我国市场经济转型过程中存在不同程度的道德失范问题(例如，假冒商品、虚假广告等)使得企

*本文得到国家社科基金重大项目“我国市场导向的绿色技术创新体系构建研究”(20&ZD059)、国家自然科学基金优秀青年科学基金“创新管理”(71922016)和国家自然科学基金面上项目“中小企业开放式创新生态系统种群演化与平衡机理研究：生态重塑视角”(71772118)等项目的资助。朱琪玮为本文通讯作者。

(C)1994-2021 China Academic Journal Electronic Publishing House. All rights reserved.　http://www.cnki.net

业绿色创新进程步履维艰。然而,本土和谐理论的诞生与发展为绿色创新研究者与实践者提供了新的思路。根据和谐理论,在复杂多变的动态环境下,企业为实现长期发展战略目标,以"和谐"为价值判断,依据"和谐机制"采取不同的措施以应对管理过程中的行为规范趋同与整体优化设计问题(席酉民等,2020)。与该理论相对应的"和谐共生"理念强调经济活动应顺应生态法则,形成与自然环境和谐共生的科学发展状态;它在发展目标上与"天人合一"的绿色治理观相契合,在规模效应上与"道法自然"的规律法则相协调,在体系构建上与"仁义礼智"的伦理道德相一致(李维安等,2019;任起顺,2009;Xing and Starik,2017)。因此,立足本土"和谐共生"文化,深入探究中国企业如何有效践行绿色创新以提升可持续发展绩效成为当前亟须探究的重要问题。

纵观已有研究,有关绿色创新的理论文献主要基于"外界压力—绿色行为—企业绩效"的经典理论逻辑,以绿色发展为主线,运用制度理论和利益相关者理论识别政府、股东、消费者等对企业绿色创新的驱动作用(Hojnik and Ruzzier,2016);以及运用资源基础观剖析绿色创新对财务绩效(Xie et al.,2016)或环境绩效的作用机理(Seman et al.,2019)。然而,财务绩效与环境绩效的协同发展更能评估企业是否满足可持续性要求:一方面,它诠释了企业创造最佳财务绩效以确保其在市场上的长期生存能力;另一方面,它诠释了企业基于减少环境负担甚至产生环境效益的技术而提供产品或服务的能力(Ilias et al.,2018)。因此,本文同时采用财务绩效和环境社会责任绩效全面衡量企业的可持续发展绩效。尽管近年来学术界逐渐开始关注绿色创新对企业可持续绩效的综合影响(Fernando et al.,2019),但融入时间维度的影响机制研究较少;此外,不同类型的绿色创新对企业可持续发展绩效的影响差异也需进行更加深入的探讨。一方面,绿色创新是一个相对动态的过程,其长期和短期的影响路径存在差异(Van den Bergh,2013),因此,基于中长期的面板数据模型对于拓展绿色创新理论研究不可或缺;另一方面,不同类型的绿色创新往往具有不同的比较优势,例如,绿色工艺创新可以为企业带来技术领先优势(Xie et al.,2019),而绿色产品创新可以为企业带来市场领先优势(Lin et al.,2013)。因此,本文拟在"绿色创新—企业绩效"理论框架下探究不同类型的绿色创新对企业可持续发展绩效的影响是否存在差异。

本文另一个研究问题在于回答绿色创新通过何种内在机制影响企业的可持续发展绩效。已有研究往往基于合法性视角探讨绿色创新的影响因素,包括环境法规(Yu et al.,2017)和市场绿色需求等(Lin et al.,2013);此外,一些研究指出,合法性作为企业的战略资源可以显著提升企业绩效(Wei et al.,2017;Zhang et al.,2018)。可见,合法性可以作为链接绿色创新与企业可持续发展绩效的桥梁。同时,从信号传递角度看,企业实施绿色创新不仅会对外界传达积极遵守环境法规以及满足绿色需求的意愿;而且还会借助绿色创新向外界传达履行社会责任等合法化的信号,从而获得政府和消费者等利益相关者的认可与信任,有利于企业获取自身发展所需的关键资源。因此,本研究采用合法性理论(Legitimacy theory)作为分析框架,用于诠释绿色创新影响企业可持续发展绩效的内在机制。此外,为解决制度和效率之间的悖论,学者们尝试基于"适应合法性"和"战略合法性"两大理论视角来探讨企业合法性问题(Suchman,1995;Zimmerman and Zeitz,2002)。适应合法性(Conforming legitimacy)强调"遵从性"(Conformity),认为合法性是企业生存与发展的前提,企业只有遵守制度管制,才能缓解外部压力的冲击(DiMaggio and Powell,1983;Meyer and Rowan,1977);战略合法性(Strategic legitimacy)强调"能动性"(Initiativeness),认为合法性是一种企业可利用的稀缺资源,对企业绩效具有重要影响(Ashforth and Gibbs,1990;Tolbert and Zucker,1983;Tornikoski and Newbert,2007)。在实践中,鉴于扮演的环境责任角色的差异,企业会表现出不同程度的绿色创新行为。例如:当仅仅满足政府规制要求时,企业往往执行"浅绿色"(Light green)的创新战略——即被动采取与制度或社会认知相一致的绿色行为;当面对绿色需求时,企业会实施"中绿色"(Medium green)的创新战略——即对市场做出积极响应;当以承担社会责任为己任时,企业会进行"深绿色"(Dark green)的创新战略——即充分发挥能动性,协同各方资源以实现战略目标。由此可见,企业在进行绿色创新过程中会兼顾合法化的"二元性",即"适应性"和"战略性";因此,本文拟从"适应合法性"和"战略合法性"整合的理论视角探讨二元合法性在绿色创新与企业可持续发展绩效关系之间的作用机理。

此外,绿色创新对企业可持续发展绩效的作用机制存在情境依赖,因此,本文关心的第三个研究问题是探

(C)1994-2021 China Academic Journal Electronic Publishing House. All rights reserved. http://www.cnki.net

讨影响绿色创新与企业可持续发展绩效关系的边界条件。虽然以往文献已经引入多种理论拓展绿色创新的情境机制，例如，资源基础观（Chen et al.，2006）、动态能力理论（Yu et al.，2017），但基于二元合法性理论的情境研究仍较缺乏。二元合法性描述了企业从社会系统中获取与整合重要资源的过程：“适应合法性”强调企业通过获取符合社会期待的行动等方式获得合法性；“战略合法性”则强调企业通过借助激励性符号向外界传达合法化信号来主动获得合法性（Czinkota et al.，2014）。在具有“尊礼重道”等道德规范的中国特色传统文化背景下，面临经济转型过程中存在的道德缺位和道德失范问题，环境法规的有效实施离不开伦理道德的支持，即“德法兼备”是合乎我国现实国情的战略导向。而企业的伦理道德源自伦理型领导的战略导向：伦理型领导不仅能够发挥“规制阐释”作用，即通过“意义给赋”影响企业内部员工对绿色创新实践的认知（徐建中等，2017），而且承载着企业的声誉和形象，可以视为企业向外界释放的“激励性信号”（Fu et al.，2020）。因此，企业可以通过任命倡导可持续发展的伦理型领导向政府、顾客等利益相关者传递企业是值得信赖的信号。由此，探讨以伦理道德和社会责任为核心特征的伦理型领导在实施绿色创新过程中的作用具有重要的现实意义。因此，我们提出，倡导绿色创新的伦理型领导能够影响企业内部成员对绿色创新的承诺、认同与投入，从而促进企业绿色创新转化为实际的成果产出，以此提高企业的合法性。因此，结合二元合法性理论，本文拟探究伦理型领导在“绿色创新—二元合法性—可持续发展绩效”逻辑链条中的边界机制。

综上，本文立足于当前制度与经济转型现状，拟采用内容分析法挖掘我国重污染制造业上市公司2013-2018年企业社会责任报告披露的环境信息，探讨绿色创新对企业可持续发展绩效的作用机理；尤其是基于二元合法性理论视角，验证二元合法性的中介效应与伦理型领导的调节效应。由此，本研究拟产生如下理论贡献：（1）本研究通过聚焦绿色创新的细分维度揭示不同类型的绿色创新对企业二元合法性和可持续发展绩效的影响差异，丰富了绿色创新与企业绩效关系的研究，深化了现有绿色创新理论研究；（2）本研究通过引入二元合法性理论，检验“适应合法性”和“战略合法性”在绿色创新和可持续发展绩效之间的中介作用，拓展了绿色创新发挥价值的渠道，揭开了绿色创新与企业可持续发展绩效之间的“理论黑箱”；（3）通过检验伦理型领导在绿色创新影响企业可持续发展绩效过程中的边界作用，拓展了现有绿色创新理论研究的边界，完善了绿色创新对企业绩效影响过程的理论外延。此外，本研究拟产生如下管理启示：（1）深化了企业对可持续发展绩效驱动因素的理解，为企业发挥不同类型绿色创新对可持续发展绩效的积极作用提供理论依据；（2）为企业将绿色创新、二元合法性、伦理型领导以及可持续发展绩效纳入整体框架进行系统管理提供了可能，也为有效改善企业绩效以及促进企业可持续发展开辟了新的思路。

二、理论基础与研究假设

（一）绿色创新与可持续发展绩效

绿色创新（Green Innovation）是由新的或改良的产品、流程、服务和管理等组成的创新，它既能为客户和企业实现增值，也能显著降低对环境的不利影响（Hojnik and Ruzzier，2016）。不同于传统创新，绿色创新更强调采用新技术、新理念以实现资源的高效利用和污染的有效降低，同时获得相应的经济绩效（王彩明、李健，2019）。此外，绿色创新的宗旨是产生良好的环境效益，而不仅仅是降低环境压力（Driessen et al.，2013）。因此，绿色创新日益被企业视为在全新竞技场中获得可持续竞争优势的重要战略（Fernando et al.，2019；Zhu et al.，2012）。依据已有研究，绿色创新可以划分为绿色工艺创新和绿色产品创新（El-Kassar and Singh，2019）：其中，绿色工艺创新（Green Process Innovation）包括清洁生产技术创新和末端治理技术创新，旨在通过改进现有生产工艺或开发新工艺来减少有害物质产生、降低污染物排放以及提高能源使用效率（Xie et al.，2019）；而绿色产品创新（Green Product Innovation）强调将环保理念融入原材料选择、产品设计、产品包装等各个环节，旨在减轻整个产品生命周期所产生的负面环境影响（Chan et al.，2016；Lin et al.，2013）。已有研究基于资源基础观对绿色工艺创新和绿色产品创新的作用机制进行了探讨，包括绿色工艺创新对企业财务绩效的影响（Xie et al.，2016）；绿色工艺创新和绿色产品创新对企业环境绩效（Huang and Li，2017）以及竞争优势的影响（Chen et al.，

(C)1994-2021 China Academic Journal Electronic Publishing House. All rights reserved. http://www.cnki.net

2006）。然而，已有研究主要强调绿色创新的重要性，忽略了不同类型绿色创新作用机制的深层次差异性比较。

尽管绿色创新的两个维度对企业可持续发展绩效发挥作用的途径不同，但均会产生积极影响。就绿色工艺创新而言，该创新战略通过使用替代能源、改善工艺以及资源循环使用等途径能够有效提高能源利用率、降低废弃物产生率，确保企业的生产制造流程符合环境规制，从而规避环境污染处罚（Xie et al.，2016；Yu et al.，2017）；同时，绿色工艺创新可以通过改良的工艺提高生产效率，并通过减少资源使用促进成本最小化（Xie et al.，2016）。此外，作为一项前沿技术创新，绿色工艺创新能够为企业带来技术领先优势以及提高环境治理能力（Chiou et al.，2011）。就绿色产品创新而言，该创新战略通过采用环保材料能够降低产品在使用过程中的能耗，构建更完善的回收处理体系（Chan et al.，2016），从而降低产品在整个生命周期过程中对环境的不利影响（Chen et al.，2006）。更重要的是，绿色产品突出的环保特性有助于企业构建差异化竞争优势（Porter，1991），包括树立良好的绿色形象以及提高利益相关者对企业环境表现的信任度等；尤其是绿色产品创新带来的差异化产品优势可以帮助企业获得环境溢价，从而提高企业财务绩效（宗计川等，2014）。综上，在绿色创新实施过程中，企业不仅可以通过绿色工艺创新从源头节约资源成本，改善工艺技术，并实现环境保护；而且可以通过绿色产品创新获得有竞争力的环境溢价，并树立良好的企业形象，从而提高企业的可持续竞争优势。据此，提出以下假设。

H1a：绿色工艺创新在改善环境社会责任绩效的同时有利于提升企业财务绩效，从而对企业可持续发展绩效产生积极影响。

H1b：绿色产品创新在提升企业财务绩效的同时有利于改善环境社会责任绩效，从而对企业可持续发展绩效产生积极影响。

绿色工艺创新和绿色产品创新作为绿色创新的两个维度，两者追求的环境目标不同（Arfi et al.，2017）；而环境目标的差异会导致绿色投资偏好程度和能源约束程度存在差异，进而对企业财务绩效和环境社会责任绩效的改善空间产生不同的影响。由此，本文提出，相比绿色工艺创新，绿色产品创新能够为企业带来更多差异化的产品和资本投资组合，进而更好地提升企业的财务绩效；而相比绿色产品创新，绿色工艺创新的两个层面——清洁生产技术与末端治理技术能够帮助企业更有效地突破资源约束以达到政府节能减排要求，从而更好地改善环境社会责任绩效。一方面，从市场需求的驱动机制来看，相比绿色工艺创新，绿色产品创新主要是针对用户的市场需求进行产品开发，从而强化产品的差异化优势（Li et al.，2019）。这种优势不仅有助于企业开拓新市场和提升市场占有率，而且能够满足用户对产品品质与环保意识的追求，进而带来更多的环境溢价（宗计川等，2014）。与此同时，资本市场的“绿色投资”也越来越偏好流向更容易产生差异化绿色产品的企业（周方召、戴亦捷，2020），因此，相比绿色工艺创新，绿色产品创新能够帮助企业吸引更多投资者，降低企业产品研发成本和风险，从而带来更好的财务绩效。另一方面，从节能减排的监管机制来看，相比绿色产品创新，绿色工艺创新不仅侧重采用清洁能源和引进节能设备等源头生产技术缓解非再生能源的约束程度，以此更有效地达到政府环境规制要求；而且致力于末端污染物的有效治理，从而降低企业生产末端排放的污染物，获得良好的企业绿色形象（Xie et al.，2019）。此外，绿色工艺创新还致力于精简工艺流程，从而缩短生产时间，提高生产效率，避免能源的过度消耗（Yu et al.，2017）。因此，绿色工艺创新更能提高企业的能源利用率，降低污染物排放，获得更好的环境社会责任绩效。据此，提出以下假设。

H1c：相比绿色工艺创新，绿色产品创新对提升企业财务绩效的作用更显著。

H1d：相比绿色产品创新，绿色工艺创新对提升企业环境社会责任绩效的作用更显著。

（二）基于二元合法性理论的中介机制与调节机制

1.适应合法性与战略合法性整合的二元合法性理论基础

美国学者Suchman（1995）将合法性定义为：“在一个由规范、价值、信念和定义构建的社会体制内，认定一个实体的行为是可取的、恰当的、合适的等一般性的感知和假定”。从组织层面，合法性源于组织与其所嵌入的社会环境期望之间的一致性，是组织获得社会环境中资源拥有者的认可程度（冯天丽、井润田，2009）。此

(C)1994-2021 China Academic Journal Electronic Publishing House. All rights reserved. http://www.cnki.net

外,合法化是制度理论最重要的假设之一,因而,有关合法性的最初研究是以“制度理论”为视角,强调企业是嵌入特定制度环境之中,只有顺应制度环境才能获得合法性以维系其生存与发展(DiMaggio and Powell,1983;Meyer and Rowan,1977)。这种以“制度逻辑”为出发点的合法性理论被称为“适应合法性”,即将合法性视为企业需要遵守相关规范和行为准则的一种约束(Tornikoski and Newbert,2007)。该理论强调政府在企业经营过程中的重要作用,但忽略了企业在实践中的主观能动性。随着研究深入,学者们发现,组织合法性的提升不仅需要通过企业行为趋同(强制趋同、模仿趋同以及规范趋同)来获得相关社会参与者的认可,进而获取自身所需资源,还需要通过最优资源的选择和配置来高效利用资源,将资源投入转化为创新产出,并创造一个值得信赖的组织形象,以此获得持续性竞争优势(Ashforth and Gibbs,1990;Tolbert and Zucker,1983;Tornikoski and Newbert,2007)。通常,经济转型中的企业需要通过两种经营战略来获取合法性:一是创建关系网络尤其是政治关联,以改善企业的制度环境;二是通过慈善捐助等改善自身形象和经营模式(冯天丽、井润田,2009;Tornikoski and Newbert,2007)。因此,合法性理论得到了新的诠释,即合法性被认为是企业的关键资源,能够帮助企业获得生存和发展所需的资源,包括资本、信息、技术、政府支持以及顾客信赖等(Zimmerman and Zeitz,2002)。不同于“适应合法性”,这种以“效率逻辑”为出发点的合法性理论被称为“战略合法性”,即强调合法性是一种通过企业战略来获得的资源,聚焦企业如何主动采取合法化行为以快速协调相应资源(Tornikoski and Newbert,2007)。该理论强调企业的能动性在合法性获取过程中的重要作用,但缺乏对外部环境影响的诠释。由此,学者们开始从“制度逻辑”与“效率逻辑”整合的视角研究二元合法性问题——即适应合法性和战略合法性(Tornikoski and Newbert,2007)。二元合法性既反映了各方利益相关者对企业行为是否合乎期望而做出的肯定性评价(Suchman,1995),同时反映了企业对资源要素投入和分配是否合理而做出的资源协调战略(Tornikoski and Newbert,2007;魏江等,2020)。

然而,有关绿色创新的研究主要基于适应合法性视角探讨政府的环境法规对企业绿色创新的驱动作用(Hojnik and Ruzzier,2016),而关于企业如何运用相关战略主动获取合法性的研究并未得到广泛探讨。本研究提出,在中国特色制度文化背景下,政府始终扮演着重要角色,因此,企业不可避免地要遵从环境法规。然而,随着经济转型的推进,企业从消费者手中获得的“环境溢价”会降低合法性的成本,提升自身的竞争优势,因此,企业会主动抢占绿色市场并通过对资源优化设计获得所需资源。鉴于此,本研究的绿色创新战略不仅代表了企业被动遵守政府规制,而且代表了企业主动获取合法性的有效策略:首先,实施绿色创新意味着企业具有履行环境社会责任的意识(Poussing,2019);其次,在环保主义兴起的背景下,绿色创新更容易被看作是通用的认知框架和企业特性(即“存在的理由”);最后,企业实施绿色创新是对政府给予绿色补贴、客户购买“溢价”型环保产品的合理“回馈”。而合法性不仅决定了利益相关者如何看待企业,还决定了利益相关者如何认识和理解企业的经济行为。由此,政府、消费者等利益相关者往往会认为具备合法性的企业更可预测、更有价值、更具有社会使命以及更值得信赖。由此,本研究将从二元合法性理论视角探讨绿色创新与企业可持续发展绩效之间的内在机理和边界条件,以期更好地回答为什么采取绿色创新的企业更可能实现可持续发展,以及企业如何才能更有效地从绿色创新战略中赢得声誉和经济价值。

2.二元合法性的中介效应

根据本土和谐理论,企业实施绿色创新旨在解决企业与自然的“和谐共生”问题,即“和则”与“谐则”机制问题:其中,“和则机制”蕴含了同行的规范模仿、政府激励政策等能够促使企业保持与社会期望行为趋同的规范准则;而“谐则机制”体现了高效的生产流程与组织结构等能够优化企业资源配置的战略体系(席酉民等,2020;El-Kassar and Singh,2019)。与之相对应的二元合法性的“制度逻辑”与“效率逻辑”也体现了“和谐共生”思想:“和”意味着“合意”,即企业的行为在社会系统中合意;“谐”意味着“协调”,即企业通过能动性对资源要素进行整体优化与协同;“共生”意味着“循环再生”,即企业与社会生态系统形成良性循环,从而实现可持续发展。因此,下面基于“制度逻辑”与“效度逻辑”的二元合法性理论视角,立足本土“和谐共生”文化,深入探究绿色创新与可持续发展绩效的内在机制。

(C)1994-2021 China Academic Journal Electronic Publishing House. All rights reserved. http://www.cnki.net

(1)适应合法性的中介效应。“制度逻辑”旨在资源获取,是指在绿色创新过程中企业通过“和则机制”将其行为与社会期望趋于一致,提高企业行为的“社会合意度”,进而增强其适应合法性,提升企业的可持续发展绩效。一方面,企业的绿色创新实践能够提高其适应合法性。具体如下:绿色工艺创新通过工艺改善等途径实现从源头上遏止污染以及有害废弃物产生的环境治理目标(Xie et al.,2016),促使企业达到清洁生产和污染排放降低等环境法规标准,由此提高企业的适应合法性(Shu et al.,2016)。绿色产品创新则以降低产品生命周期中产生的消极环境影响为最终目的(Chan et al.,2016),通过使用环保材料、优化包装设计以及回收再制造等方式降低能源损耗,改善产品质量,从而获得客户等利益相关者对企业环境行为的认可(Wei et al.,2017;Shu et al.,2016)。

另一方面,依据“制度逻辑”,适应合法性能够提高企业的可持续发展绩效。基于我国政府在经济活动中的主导地位,适应合法性能够为企业提供大量稀缺资源和优惠待遇以促进企业成长(郭海等,2018;Wei et al.,2017)。近些年来,我国政府一直将“绿色发展”作为我国制造业未来发展规划的一项基本方针。这表明在我国制造业绿色转型的关键节点,企业通过采用与政府环境规划相一致的环境战略,有助于企业获得政府的稀缺资源,包括税收减免、污染治理专项款、软贷款、行业准入许可等资源和相关技术支持(Xie et al.,2016)。此外,获得政府专属认可能够有效避免政府对其经营活动的过多干预,同时政府与消费者对企业产品的认同能够为企业进行市场扩张和进入新市场提供便利,从而促进企业可持续发展绩效的增长(Wei et al.,2017;Sheng et al.,2011)。

综上,适应合法性不仅反映了企业的行为与社会规制的一致性,而且能够通过“制度逻辑”缓解社会环境体制压力,赢得利益相关者的认可(Suchman,1995);并且,感知到绿色创新价值的政府会将合法性作为一种公平的回报反馈给企业;有助于企业更易获得生存和发展所需的资源,从而促进企业的可持续发展绩效。由此,本研究认为,绿色创新影响企业可持续发展绩效过程的重要传导机制在于适应合法性。据此,提出以下假设。

H2a:绿色工艺创新能够通过为企业获得更高的适应合法性,从而促进企业可持续发展绩效的提升。

H2b:绿色产品创新能够通过为企业获得更高的适应合法性,从而促进企业可持续发展绩效的提升。

(2)战略合法性的中介效应。“效率逻辑”旨在资源协同,是指在激烈的市场竞争环境中企业通过积极承担环境社会责任,营造良好的企业形象,并通过“谐则机制”协调整合与优化各方资源,提升企业的核心竞争优势。因此,战略合法性也是解释企业绿色创新过程中可持续发展绩效改善的重要原因。一方面,企业的绿色创新实践能够提高其战略合法性,具体如下:绿色工艺创新能够帮助企业实现资源节约、环境保护的战略目标,有效避免企业环境污染的负面媒体报道,营造良好的绿色形象(Xie et al.,2016);并通过技术领先优势获得同行信任,从而有助于企业构建牢固的关系网络(Wei et al.,2017;Shu et al.,2016);而绿色产品创新则代表企业向社会各界传递主动履行环境社会责任的信号,能够满足甚至创造客户的绿色需求,从而提升消费者的剩余价值。

另一方面,战略合法性能够提高企业的可持续发展绩效。首先,战略合法性高的企业往往拥有较高的资源协调能力,能够保证资源要素在企业经营过程中的合理投入以及快速产出;并且战略合法性可以提高企业的议价能力,从而降低经营成本。其次,企业具有良好的战略合法性能够促使顾客感知的企业价值观与企业内部价值观保持一致,从而达成情感共鸣,提高消费者的购买意愿和黏性(Wei et al.,2017)。再次,较高的战略合法性能够帮助企业建立良好的社会关系网络,从而为其带来多元化信息和更广泛的知识储备(Sheng et al.,2011)。具体而言,战略合法性能够促进企业与外界进行密切的社交互动(Huang and Li,2017),促使企业协调、整合各方利益相关者提供的信息和资源,进而有助于企业重构自身的知识体系,把握市场先机(Huang and Li,2017),提升企业的可持续发展绩效。

综上,战略合法性诠释了企业履行环境社会责任的能动性,通过“效率逻辑”提高了资源使用效率,积极响应了市场导向;且感知绿色创新价值的消费者会将可观的环境溢价作为对企业环保产品的合理回馈。此外,战略合法性有助于企业达到引导社会舆论的目的,更好地提升企业声誉和绿色形象。由此,本研究认为,绿色创新影响企业可持续发展绩效过程的另一重要传导机制在于战略合法性。据此,提出以下假设。

(C)1994-2021 China Academic Journal Electronic Publishing House. All rights reserved. http://www.cnki.net

H2c：绿色工艺创新能够通过为企业获得更高的战略合法性，从而促进企业可持续发展绩效的提升。

H2d：绿色产品创新能够通过为企业获得更高的战略合法性，从而促进企业可持续发展绩效的提升。

3.伦理型领导的调节效应

伦理型领导（Ethical Leadership）的概念最早是由Enderle（1987）提出，旨在明确刻画管理者在决策过程中的伦理问题，并且对决策过程中所参照的原则加以规范。根据Brown等（2005）的观点，伦理型领导具备两个特征：“伦理个人”和“伦理管理者”，即伦理型领导是指具备诚实、正直及关爱等符合社会规范行为的管理者；同时他们通常会以身作则并且鼓励与引导员工表现出类似行为。在中国传统文化背景下，伦理道德与“和谐共生”思想逻辑趋向一致：“仁者爱人”的伦理原则体现了克己复礼等人性信念，而“道法自然”的伦理共识则揭示了善待自然等法律规制（任起顺，2009；Xing and Starik，2017）。此外，Jones等（2017）提出，在可持续发展背景下，伦理框架涵盖两个重要部分：关怀伦理（Ethics of Care）与可持续发展伦理（Ethics of Sustainability）。因此，本研究提出，伦理型领导不仅应具有正直、诚信、公正等伦理特征（Brown et al.，2005），还应具有提倡环保和关注可持续发展的特质（Jones et al.，2017），即需要同时体现企业领导者的人文主义和环保主义。

首先，本文提出，绿色创新与企业适应合法性之间的关系会受到伦理型领导的影响。伦理型领导所塑造的企业文化有助于改善企业的社会责任绩效，使得企业活动能够达到多方利益相关者的期望水平（Pasricha et al.，2018）。然而，不同的领导者会因对环境规则的不同解读（例如，视环境规制为消极的阻碍性压力或积极的挑战性压力）而表现出不同的回应（主动支持、被动适应或消极抵触），进而对企业绿色创新的实施过程产生不同的影响。首先，基于“意义给赋”（Sense-giving）机制，伦理型领导更能准确地识别社会系统对环境规范的要求与期望；并且通过领导者的“伦理模范”促使内部员工模仿学习，让更多的员工认同绿色生产的必要性，从而提升企业整体的环保意识（徐建中等，2017）。因此，企业更可能采用清洁能源与环保设备，不断提升绿色工艺创新水平，以有效进行符合环保规范的经济活动，提升企业的“社会合意度”，即适应合法性。其次，基于“情感共鸣”（Emotional Resonance）机制，伦理型领导更易得到员工的“信任回报”，与之相应，员工也会觉得更有义务表现出对企业有利的工作行为，努力提升自己的业务水平（仲理峰等，2019）；并且，伦理型领导的“赏罚分明”会督促员工力行资源节约，进而强化企业整体的环保行为。因此，企业的绿色产品质量更易得到保障；从而确保企业绿色产品创新被政府认可，提高企业的适应合法性。据此，提出以下假设。

H3a：伦理型领导在绿色工艺创新与适应合法性关系中发挥正向调节作用。

H3b：伦理型领导在绿色产品创新与适应合法性关系中发挥正向调节作用。

其次，本文提出，绿色创新与企业战略合法性之间的关系会受到伦理型领导的影响。伦理型领导正直、公平和诚实的处事原则不仅有助于形成节约资源的企业文化氛围，促进成员之间的沟通交流、知识共享，进而激发新颖的想法，提高企业资源的利用率（Mo et al.，2019）；而且会影响整个社会环境保护的文化氛围，从而营造良好的企业形象。在企业运营过程中，伦理型领导可以利用对关键资源的协调权利，影响企业内部员工的工作行为，进而影响企业绿色创新活动的实施。一方面，基于“资源协调”（Resource Coordination）机制，伦理型领导更倾向支持企业员工进行绿色创新活动，并给员工提供所需的资源，由此提升员工的绿色创造力，获得员工对绿色工艺改善的建议（Tu and Lu，2013）。此外，伦理型领导的言传身教会感染且触发员工的道德认同，激发员工表现出较强的环保担当责任（Schaubroeck et al.，2012）。因此，企业更可能实现资源的有效利用，以更快的速度将资源投入转化为创新产出，从而在绿色市场中赢得主动先机，提升企业的战略合法性。另一方面，基于“信号传递”（Signalling）机制，伦理型领导更倾向于关怀下属员工，在员工心中塑造公正、赏罚分明的领导形象（Mayer et al.，2012），从而为企业营造良好的伦理文化氛围，有利于企业绿色产品创新的开展，提升企业的战略合法性。此外，伦理型领导承载着企业积极履行社会责任意愿的传递，帮助企业建立高质量的社会网络关系（仲理峰等，2019），从而加快绿色产品推广，提升企业的战略合法性。因此，在企业实施绿色创新过程中，受到伦理型领导关怀的员工会有很强的环保意愿，并且他们会将这种环保意愿转化到自己的工作任务中，从而促使企业的绿色创新活动被公众认可，进而强化绿色创新与战略合法性之间的积极关系。据此，提出以下假设。

(C)1994-2021 China Academic Journal Electronic Publishing House. All rights reserved. http://www.cnki.net

H3c:伦理型领导在绿色工艺创新与战略合法性关系中发挥正向调节作用。

H3d:伦理型领导在绿色产品创新与战略合法性关系中发挥正向调节作用。

综上,企业绿色创新实践(绿色工艺创新、绿色产品创新)可以通过提升合法性水平(适应合法性、战略合法性)来改善企业的可持续发展绩效;而在企业经营过程中,领导者的伦理道德不仅关系到员工的利益,而且影响企业的声誉,是企业向政府和客户等利益相关者传递"企业是值得信任和认可"的重要信号(仲理峰等,2019)。因此,本文提出,伦理型领导会影响合法性在绿色创新与可持续发展绩效关系之间的中介效应(如图1所示)。具体而言,当企业的领导者具有关怀伦理与可持续伦理特质时,绿色创新对合法性的边际效应会增大,进而绿色创新对企业可持续发展绩效的"和谐"机制也会强化。据此,在H3a~H3d基础上,本研究进一步提出如下假设。

H4a:伦理型领导正向调节适应合法性在绿色工艺创新与可持续发展绩效之间的中介关系。

H4b:伦理型领导正向调节适应合法性在绿色产品创新与可持续发展绩效之间的中介关系。

H4c:伦理型领导正向调节战略合法性在绿色工艺创新与可持续发展绩效之间的中介关系。

H4d:伦理型领导正向调节战略合法性在绿色产品创新与可持续发展绩效之间的中介关系。

三、研究设计

(一)样本选择与数据来源

本研究数据主要来源于上交所或深交所A股上市制造业企业。鉴于我国制造业上市公司对环境信息的披露暂无统一范式,借鉴现有关于企业环境信息披露的研究方法(Albertini,2014;Mallin et al.,2013),除了源自Wind等数据库的二手数据,本文主要采用内容分析法对制造业重污染上市公司2013~2018年的企业社会责任报告进行数据挖掘。为了获取可靠而全面的数据,本研究通过以下步骤筛选研究对象:(1)通过Wind数据库证监会制造业分类获得按行业分类排序的上市公司名录,共2300家;(2)根据《上市公司环保核查行业分类管理名录》(环办函〔2008〕373号),进一步筛选出我国制造业重污染上市公司,共1205家;(3)删除有ST和PT的上市公司及2013~2018年未公布企业社会责任报告的上市公司;最终获得227家上市公司研究数据,其中,金属非金属行业、石化塑胶行业和医药制造行业的样本比重较大,占比依次为26%、23%和16%,其次为汽车制造业、纺织服装皮毛业、食品制造和农副产品加工业以及酒、饮料和精制茶制造业的上市公司,占比依次为9%、9%、8%和6%,造纸及纸制品行业上市公司占比为3%。尽管如此,仍有53家上市公司因未按年度披露企业社会责任报告或其他财务信息不全等导致数据存在缺失。为保证样本容量,本研究采用回归插补法对缺失值进行填补,最终获得由227家重污染制造业上市公司2013~2018年度共1362个研究样本构成的平衡面板数据。

(二)研究方法

内容分析法(Content Analysis)是一种对研究内容进行客观、系统量化并加以描述的研究方法(Albertini,2014;Mallin et al.,2013)。近些年,内容分析法被广泛应用于研究企业的环境信息披露问题。本文采用内容分析法对企业披露的社会责任报告进行数据挖掘,具体步骤如下:(1)确定研究目标、选择样本和信息载体。根据研究内容,本文研究目标是通过采用内容分析法获取绿色创新、二元合法性

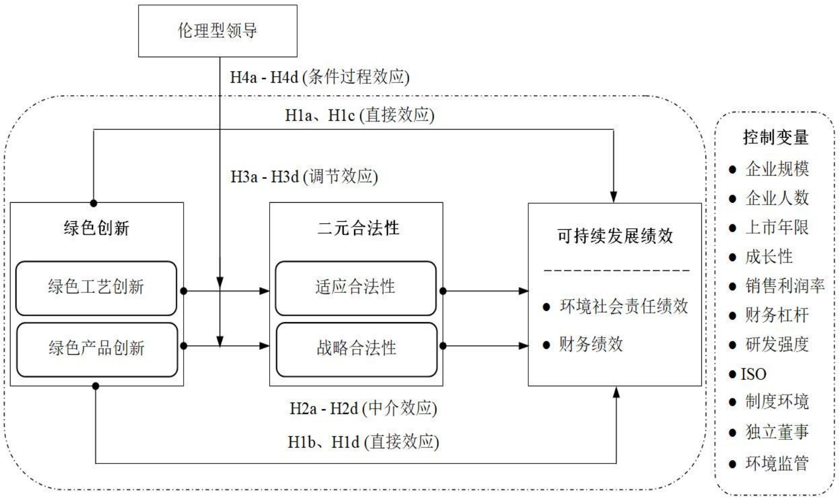

图1 理论模型

(C)1994-2021 China Academic Journal Electronic Publishing House. All rights reserved. http://www.cnki.net

和伦理型领导等变量的数据;并选择企业社会责任报告作为信息载体进行数据量化。(2)明确类目和分析单元,制定编码规则。内容分析法要求各变量的衡量指标应具有详尽且互斥特点,本研究通过对大量文献的梳理明确各变量含义,并且使用现有文献的成熟量表来衡量相应变量,从而确保编码工具的可靠性。此外,根据Albertini(2014)和Mallin等(2013)提出的内容分析法编码准则,本研究对各个变量制订了评分标准。(3)预编码与信度分析。编码过程是对文本信息的量化过程,结合内容分析法编码过程的操作要求,本研究通过以下措施提高编码者信度:一是邀请4位具有相关研究背景的编码者共同参与编码过程;二是对4位编码者进行培训,向编码者详细阐述研究内容、变量及其衡量指标的含义、编码规则和操作流程,以确保编码者对研究内容的认知和熟悉程度保持一致;三是使用编号为1~20的企业社会责任报告进行预编码试验,由4位编码者同时且独立完成对20份企业社会责任报告的编码,以检验编码者之间的信度。本研究使用Krippendorff α系数来检验编码者之间的信度(Krippendorff,2018)。检验结果显示,绿色创新、二元合法性和伦理型领导对应的各个衡量指标的Krippendorff α系数均大于0.667,说明由内容分析法获得的数据具有良好的可靠性(Krippendorff,2018)。

(三)变量测度

本研究的绿色工艺创新、绿色产品创新、适应合法性、战略合法性和伦理型领导的数据是通过内容分析法挖掘的一手数据;其他变量均来源于Wind等数据库的二手数据。以下对各个变量的测度以及衡量方法进行具体说明(详见表1)。

1.绿色创新

根据Chiou等(2011)研究观点,绿色创新划分为绿色工艺创新和绿色产品创新两个维度;其中,绿色工艺创新采用5个指标进行测度;绿色产品创新采用4个指标进行衡量。此外,引用Mallin等(2013)使用内容分析法量化环境信息的编码准则,绿色工艺创新和绿色产品创新的赋值方法如下:如果企业社会责任报告对相关指标有文字描述,评分为1;如果涉及量化或详细深入的描述,评分为2;如果没有描述,评分为0;最终数值分别为所有指标的评分总和。

2.可持续发展绩效

参考Ilias等(2018)研究,本文将可持续发展绩效划分为财务绩效和环境社会责任绩效两个维度。如表1所示,参照Xie等(2016)研究,本研究采用总资产报酬率(ROA)衡量财务绩效,并用百分比形式表示(邵帅、吕长江,2015);参照贾兴平和刘益(2014)的研究,本研究采用第三方机构发布的企业社会责任评级总得分来衡量企业的环境社会责任绩效。

3.二元合法性

参考Tornikoski和Newbert(2007)的研究,本文将二元合法性划分为适应合法性和战略合法性两个维度。如表1所示,适应合法性主要结合Wei等(2017)和郭海等(2018)的研究观点,设计3个指标进行测量;战略合法性主要结合冯天丽和井润田(2009)、Tornikoski和Newbert(2007)以及魏江等(2020)的研究观点,设计3个指标进行测度。同上,基于内容分析法以及引用Albertini(2014)和Mallin等(2013)的编码准则,适应合法性和战略合法性的赋值方法如下:如果企业社会责任报告有对应指标的描述,评分为1;否则为0;最终取值分别为所有指标的评分总和。

4.伦理型领导

鉴于本研究是在可持续背景下探讨伦理型领导,参照Jones等(2017)研究,本文从人文关怀导向和环境可持续发展导向两个方面衡量伦理型领导。如表1所示,基于Brown等(2005)和Wang等(2017)研究,本文分别采用4个指标对人文关怀导向伦理型领导和环境可持续发展导向伦理型领导进行测度。同上,根据Albertini(2014)和Mallin等(2013)基于内容分析法的编码准则,伦理型领导的赋值方法如下:如果企业社会责任报告有相关指标的描述,评分为1;否则评分为0;两个维度的取值分别为所有指标的评分总和,伦理型领导的最终取值则为其所有指标的评分总和。

(C)1994-2021 China Academic Journal Electronic Publishing House. All rights reserved. http://www.cnki.net

5.控制变量

鉴于企业可持续发展绩效会受到诸多因素的影响,例如组织基本特征、组织资源与研发能力等,本文选取以下变量作为控制变量。(1)企业规模(*Size*)。采用企业总资产的自然对数进行测度(徐建中等,2017)。(2)企业人数(*Numb*)。采用企业总员工人数的自然对数进行测度(郭海等,2018)。(3)上市年限(*Age*)。使用企业上市年份至企业社会责任报告汇报年份的年数进行衡量(贾兴平、刘益,2014)。(4)成长性(*Growth*)。使用营业总收入同比增长率进行衡量(邵帅、吕长江,2015)。(5)销售利润率(*ROS*)。使用营业利润与营业总收入的比值进行衡量(董静等,2017)。(6)财务杠杆(*Lev*)。财务杠杆对企业环境行为投资有着重要作用,本研究使用资产负债率进行衡量(杨兴全等,2015)。(7)研发强度(*R&D*)。研发强度代表企业的创新潜力,被视为抵御绿色创新风险和不确定性的重要因素,本研究使用研发支出总额占营业总收入的比例进行衡量(杨洋等,2015)。(8)环境管理认证(*ISO*)。ISO14001认证代表了企业绿色环保达到社会系统制定的标准,对企业绩效有着重要作用(Li et al.,2018),本研究通过全国认证认可信息公共服务平台检索样本企业研究年度期间是否获得ISO14001认证,取得该认证赋值为1,否则为0。(9)制度环境(*Inst*)。制度环境是影响企业绿色创新实践的重要因素,本文采用市场化总指数进行衡量(赵云辉等,2019)①。(10)独立董事比(*Dire*)。独立董事比例是衡量公司治理程度的一个重要指标,本文采用独立董事占董事会总人数的比例进行测度(杨兴全等,2015)。(11)环

表1 变量测度与数据来源

变量	维度	测度方法	文献来源	数据来源
绿色创新	绿色工艺创新(*Proc*)	① 低能耗,生产过程中减少了水、电、煤、石油等能源的使用<br>② 使用清洁生产技术,以节约能源和防止污染物产生<br>③ 回收、再利用和再制造材料<br>④ 制造过程中有效减少了有害物质和废弃物排放;⑤ 制造过程中有效减少了原材料使用	Chiou et al.,2011	2013~2018年企业社会责任报告(内容分析法)
	绿色产品创新(*Prod*)	① 产品改进和设计过程中选择环境友好型产品材料<br>② 对已有或新产品采用可降解型包装<br>③ 产品改进和设计过程中评估该产品是否易于回收、再利用和分解<br>④ 产品改进和设计过程中使用较少资源,并且采用绿色产品标签		
可持续发展绩效	财务绩效(*Fina*)	总资产报酬率(%)	Xie et al.,2016;邵帅、吕长江,2015	Wind数据库
	环境社会责任绩效(*Envi*)	企业环境社会责任指标评级得分	贾兴平、刘益,2014	和讯网数据库
二元合法性	适应合法性(*Conf*)	① 达到政府环保部门监管要求,获得政府认可<br>② 获得同行、供应商、销售商、中介、消费者、员工和股东等利益相关者认可<br>③ 获得社区、公众和环保机构认可	Wei et al.,2017;郭海等,2018	2013~2018年企业社会责任报告(内容分析法)
	战略合法性(*Stra*)	① 积极与政府构建战略合作关系,参与政府部门主管的环保协会,提升政府对企业的信任度<br>② 积极制定明确具体的环境管理战略规划(如绿色供应商合作协议、绿色培训等),提升供应商、消费者、员工和股东等利益相关者对企业的信任度<br>③ 积极参与环保公益活动(如慈善捐助、致力于保护环境和节约资源等),提升社区、公众和环保机构对企业的信任度	冯天丽、井润田,2009; Tornikoski and Newbert,2007;魏江等,2020	
伦理型领导(Ethi)	人文关怀导向	① 对来自员工的批评和不同意见持开放态度<br>② 将员工的利益置于最重要的位置<br>③ 与员工讨论伦理和价值问题<br>④ 在道德上树立合理处理问题的榜样	Brown et al.,2005	
	环境可持续发展导向	⑤ 关注环境问题并倡导回收利用<br>⑥ 以社会利益为重<br>⑦ 关心可持续发展问题<br>⑧ 提倡环境友好型工作方式	Wang et al.,2017	
企业规模(*Size*)	-	总资产的自然对数	徐建中等,2017	Wind数据库
企业人数(*Numb*)	-	总员工人数的自然对数	郭海等,2018	
企业上市年限(*Age*)	-	上市年份至企业社会责任报告汇报年份的年数	贾兴平、刘益,2014	
成长性(*Growth*)	-	营业总收入同比增长率	邵帅、吕长江,2015	
销售利润率(*ROS*)	-	营业利润与营业总收入的比值	董静等,2017	
财务杠杆(*Lev*)	-	资产负债率	杨兴全等,2015	
研发强度(*R&D*)	-	研发支出总额占营业总收入的比例	杨洋等,2015	
环境管理认证(*ISO*)	-	公司是否通过ISO14000认证	Li et al.,2018	信息服务平台
制度环境(*Inst*)	-	市场化指数	赵云辉等,2019	市场化指数报告
独立董事比(*Dire*)	-	独立董事人数/董事会人数	杨兴全等,2015	CSMAR数据库
环境监管(*Supe*)	-	公司是否受到环境监管部门处罚	叶陈刚等,2015	中国研究数据服务平台

(C)1994-2021 China Academic Journal Electronic Publishing House. All rights reserved. http://www.cnki.net

境监管(*Supe*)。采用企业是否受到环境监管部门处罚衡量(叶陈刚等，2015)。(12)虚拟变量：为使研究内容更加严谨，本文引入了时间虚拟变量、行业虚拟变量②和地区虚拟变量。

四、实证结果检验

(一)直接效应检验

表2为各变量的Pearson相关系数矩阵、均值和标准差，表3为绿色创新对可持续发展绩效的回归结果。模型2的结果显示，绿色工艺创新和绿色产品创新均对财务绩效具有显著的正向影响(β_1=0.071，p_1<0.1；β_2=0.370，p_2<0.01)；模型6的回归结果表明，绿色工艺创新和绿色产品创新均对环境社会责任绩效具有显著的积极作用(β_1=2.395，p_1<0.01；β_2=2.969，p_2<0.01)。在此基础上，将全模型的回归系数进行标准化处理③，以比较绿

表2 Pearson相关系数矩阵

变量	1	2	3	4	5	6	7	8	9	10	11	12	13	14	15	16	17	18
1. *Size*	1.000																	
2. *Numb*	−0.003	1.000																
3. *Age*	0.354***	0.064**	1.000															
4. *Growth*	0.053**	−0.005	−0.103***	1.000														
5. *ROS*	−0.010	−0.047*	−0.054**	0.245***	1.000													
6. *Lev*	0.516***	0.006	0.158***	−0.053*	−0.472***	1.000												
7. *R&D*	−0.143***	−0.053**	−0.172***	0.074***	0.129***	−0.172***	1.000											
8. *ISO*	0.054**	−0.001	−0.067***	−0.033	−0.003	0.062**	−0.044	1.000										
9. *Inst*	−0.081***	−0.067**	−0.111***	0.047*	0.104***	−0.176***	0.233***	0.041	1.000									
10. *Dire*	0.093***	0.017	−0.058**	0.004	0.070***	−0.006	0.017	0.047*	−0.012	1.000								
11. *Supe*	−0.001	0.030	0.003	−0.001	0.099***	−0.143***	−0.008	0.060**	0.087***	−0.066**	1.000							
12. *Proc*	0.363***	0.052*	0.115***	0.023	0.028	0.153***	−0.074***	−0.020	0.002	0.000	0.122***	1.000						
13. *Prod*	0.239***	0.080***	−0.011	0.007	−0.068**	0.156***	0.063**	0.022	0.120***	0.028	0.137***	0.397***	1.000					
14. *Conf*	0.021	0.069**	−0.090***	0.044	0.036	−0.028	0.043	0.087***	0.008	0.031	0.133***	0.068**	0.175***	1.000				
15. *Stra*	0.168***	−0.017	0.017	0.003	0.036	0.052*	0.062**	0.069**	−0.024	0.097***	0.116***	0.145***	0.245***	0.510***	1.000			
16. *Ethi*	−0.134***	0.325***	−0.028	0.010	0.051*	−0.072***	0.000	0.008	0.040	0.061**	0.035	−0.039	−0.022	0.036	0.014	1.000		
17. *Fina*	0.214***	0.063**	0.005	0.183***	0.515***	−0.176***	0.018	0.049*	0.033	0.121***	0.176***	0.283***	0.319***	0.272***	0.317***	0.018	1.000	
18. *Envi*	0.370***	0.049*	0.117***	0.052*	0.024	0.131***	0.012	−0.005	−0.033	0.081***	0.240***	0.466***	0.383***	0324***	0.429***	−0.044	0.514***	1.000
Mean	22.927	8.551	13.020	0.099	0.082	0.442	0.026	0.734	7.901	0.377	0.178	2.994	1.558	1.369	1.292	2.636	4.851	35.126
S.D.	1.300	1.216	6.049	0.233	0.162	0.202	0.020	0.442	1.976	0.079	0.380	2.027	1.589	0.545	0.693	1.447	1.955	25.989

注：***p<0.01；**p<0.05；*p<0.1。

表3 绿色创新对可持续发展绩效的直接效应检验结果

Variables	*Financial performance*				*Environmental performance*			
	模型1	模型2	模型3	模型4	模型5	模型6	模型7	模型8
Explanatory variables								
Proc		0.071* (0.041)	0.049 (0.040)	0.047 (0.039)		2.395*** (0.630)	1.992*** (0.548)	1.891*** (0.531)
Prod		0.370*** (0.056)	0.318*** (0.053)	0.284*** (0.053)		2.969*** (0.772)	2.001*** (0.674)	1.163** (0.560)
Mediators								
Conf			0.826*** (0.099)				15.388*** (1.287)	
Stra				0.676*** (0.073)				14.225*** (1.017)
Controls								
Size	0.225 (0.238)	0.049 (0.238)	−0.083 (0.231)	−0.120 (0.243)	9.514*** (3.363)	7.987** (3.111)	5.526** (2.555)	4.436* (2.369)
Numb	0.251 (0.197)	0.287 (0.182)	0.324* (0.180)	0.379** (0.174)	0.461 (2.552)	0.558 (2.119)	1.260 (1.835)	2.494 (1.597)
Age	−0.039 (0.355)	0.106 (0.317)	0.406 (0.315)	0.588* (0.311)	−13.580*** (5.106)	−13.629***(4.969)	−8.055 (4.980)	−3.512 (4.704)
Growth	0.561** (0.216)	0.640*** (0.213)	0.597*** (0.200)	0.623*** (0.198)	1.474 (2.207)	2.308 (2.200)	1.508 (1.936)	1.954 (1.680)
ROS	5.120*** (1.075)	5.310*** (1.085)	5.431*** (1.097)	5.296*** (1.017)	1.655 (3.321)	3.697 (3.177)	5.949** (3.006)	3.401 (3.473)
Lev	−1.748** (0.870)	−1.500* (0.868)	−1.616* (0.851)	−1.583** (0.761)	−11.060 (6.999)	−8.826 (6.997)	−10.990* (6.118)	−10.574* (5.714)
R&D	1.624 (5.091)	3.499 (5.003)	3.418 (4.957)	2.733 (4.732)	−13.520 (60.330)	10.640 (61.060)	9.133 (55.510)	−5.460 (49.96)
ISO	−0.214 (0.349)	−0.167 (0.315)	−0.134 (0.310)	−0.100 (0.284)	−5.603 (3.970)	−4.157 (3.756)	−3.555 (3.596)	−2.762 (2.955)
Inst	−0.271 (0.179)	−0.234 (0.162)	−0.168 (0.157)	−0.157 (0.154)	0.080 (2.079)	0.048 (1.930)	1.277 (1.682)	1.669 (1.652)
Dire	1.367* (0.719)	1.562** (0.682)	1.357** (0.622)	1.077* (0.610)	8.179 (11.01)	9.498 (10.59)	5.677 (8.872)	−0.708 (8.348)
Supe	0.571*** (0.191)	0.449** (0.191)	0.255 (0.178)	0.157 (0.184)	11.870*** (2.306)	10.520*** (2.382)	6.915*** (1.836)	4.400** (2.029)
_Cons	0.012 (6.414)	1.616 (6.168)	−0.321 (6.157)	−1.579 (6.226)	−37.140 (102.500)	−5.947 (94.389)	−42.061 (84.279)	−73.144 (76.662)
Year dummies	Included	Included	Included	Included	Included	Included	Included	Included
Industry dummies	Included	Included	Included	Included	Included	Included	Included	Included
Province dummies	Included	Included	Included	Included	Included	Included	Included	Included
R^2	0.220	0.287	0.343	0.351	0.101	0.183	0.358	0.438
F-value	7.260***	9.470***	13.550***	18.470***	14.730***	18.170***	26.970***	22.110***

注：***p<0.01，**p<0.05，*p<0.1；括号为聚类稳健性标准误(Cluster-robust standard error)；模型包含年份、行业和省份虚拟变量；样本量：1362。

(C)1994-2021 China Academic Journal Electronic Publishing House. All rights reserved. http://www.cnki.net

色工艺创新和绿色产品创新对可持续发展绩效相对效应的大小:消除量纲和数量级等差异的影响后,绿色工艺创新对财务绩效的效应(β=0.074,p<0.1)小于绿色产品创新对财务绩效的效应(β=0.301,p<0.01)[4];而绿色工艺创新对环境社会责任绩效的效应(β=0.187,p<0.01)大于绿色产品创新对环境社会责任绩效的效应(β=0.181,p<0.01)[5]。据此,研究结果表明,绿色工艺创新和绿色产品创新均对企业可持续发展绩效具有显著的正向影响;但相比绿色工艺创新,绿色产品创新对财务绩效的影响更显著;而相比绿色产品创新,绿色工艺创新对环境社会责任绩效的影响更显著,即H1a~H1d均得到验证。

(二)中介效应检验

1.适应合法性的中介效应检验

表4的模型2结果表明,绿色工艺创新和绿色产品创新均对适应合法性具有显著的积极作用(β_1=0.026,p_1<0.1;β_2=0.063,p_2<0.01)。表3的模型3和模型7结果显示,在控制绿色工艺创新和绿色产品创新下,适应合法性对财务绩效和环境社会责任绩效均产生显著的正向效应(β_1=0.826,p_1<0.01;β_2=15.388,p_2<0.01)。由此,结合H1a和H1b可以证明,适应合法性在绿色创新(绿色工艺创新、绿色产品创新)和可持续发展绩效关系之间发挥中介作用,即H2a和H2b成立。

2.战略合法性的中介效应检验

表4的模型5结果显示,绿色工艺创新和绿色产品创新均能显著提升企业的战略合法性水平(β_1=0.035,p_1<0.05;β_2=0.127,p_2<0.01)。表3的模型4和模型8结果表明,在控制绿色工艺创新和绿色产品创新下,战略合法性对财务绩效和环境绩效均具有显著的积极影响(β_1=0.676,p_1<0.01;β_2=14.225,p_2<0.01)。由此,结合H1a和H1b可以证明,战略合法性在绿色创新(绿色工艺创新、绿色产品创新)和可持续发展绩效关系之间发挥中介作用,即H2c和H2d成立。

(三)调节效应检验

表4的模型3和模型6结果表明,绿色工艺创新与伦理型领导的交互项(*Proc×Ethi*)对适应合法性和战略合法性均具有显著的正向影响(β_1=0.053,p_1<0.05;β_2=0.071,p_2<0.05)。因此,伦理型领导正向调节绿色工艺创新与适应合法性/战略合法性之间的关系,即H3a和H3c成立。此外,基于模型3和模型6的结果,对伦理型领导在绿色工艺创新与适应合法性/战略合法性的调节效应进行可视化分析(图2)。图2a表明,当伦理型领导取值高于阈值3.96或低于阈值1.64时,绿色工艺创新对适应合法性的边际效应随着伦理型领导的取值增大而增大。类似地,图2b表明,当伦理型领导的取值高于阈值3.92时,绿色工艺创新对战略合法性的边际效应随着伦理型领导的取值增大而增大。由此可见,绿色工艺创新对二

表4 伦理型领导对绿色创新与二元合法性调节效应检验结果

Variable	*Conforming legitimacy*			*Strategic legitimacy*		
Explanatory variables	模型1	模型2	模型3	模型4	模型5	模型6
Proc		0.026^{*} (0.015)	0.026^{*} (0.015)		0.035^{**} (0.018)	0.035^{**} (0.018)
Prod		0.063^{***} (0.016)	0.060^{***} (0.016)		0.127^{***} (0.022)	0.125^{***} (0.022)
Moderator						
Ethi			0.015 (0.024)			0.033 (0.030)
Interactions						
Proc × Ethi			0.053^{**} (0.022)			0.071^{**} (0.034)
Prod × Ethi			0.038 (0.029)			0.006 (0.042)
Controls						
Size	0.191^{***} (0.068)	0.160^{**} (0.066)	0.174^{***} (0.065)	0.311^{***} (0.117)	0.250^{**} (0.112)	0.264^{**} (0.111)
Numb	−0.050 (0.064)	−0.046 (0.062)	−0.049 (0.063)	−0.147 (0.103)	−0.136 (0.093)	−0.139 (0.094)
Age	-0.377^{***} (0.079)	-0.362^{***} (0.081)	-0.369^{***} (0.089)	-0.754^{***} (0.117)	-0.711^{***} (0.116)	-0.737^{***} (0.118)
Growth	0.037 (0.068)	0.052 (0.069)	0.058 (0.069)	−0.004 (0.117)	0.025 (0.117)	0.028 (0.117)
ROS	−0.183 (0.137)	−0.146 (0.133)	−0.148 (0.132)	−0.048 (0.228)	0.021 (0.233)	0.015 (0.233)
Lev	0.096 (0.212)	0.140 (0.216)	0.146 (0.211)	0.036 (0.347)	0.123 (0.357)	0.138 (0.356)
R&D	−0.291 (1.982)	0.099 (1.978)	0.018 (1.950)	0.433 (2.791)	1.132 (2.758)	0.940 (2.764)
ISO	−0.056 (0.082)	−0.040 (0.082)	−0.043 (0.081)	−0.121 (0.115)	−0.098 (0.114)	−0.103 (0.111)
Inst	−0.084 (0.056)	−0.080 (0.056)	−0.087 (0.055)	−0.125 (0.080)	−0.114 (0.077)	−0.121 (0.076)
Dire	0.217 (0.268)	0.248 (0.265)	0.257 (0.267)	0.652^{*} (0.362)	0.717^{**} (0.349)	0.723^{**} (0.353)
Supe	0.258^{***} (0.083)	0.235^{***} (0.082)	0.235^{***} (0.082)	0.475^{***}(0.092)	0.430^{***} (0.089)	0.430^{***} (0.090)
_Cons	1.932 (1.943)	2.347 (1.881)	2.184 (1.898)	4.061 (3.090)	4.724 (2.961)	4.767 (2.947)
Year dummies	Included	Included	Included	Included	Included	Included
Industry dummies	Included	Included	Included	Included	Included	Included
Province dummies	Included	Included	Included	Included	Included	Included
R^2	0.057	0.086	0.099	0.098	0.151	0.160
F-value	9.020^{***}	9.060^{***}	9.230^{***}	19.500^{***}	19.310^{***}	17.420^{***}

注:***p<0.01,**p<0.05,*p<0.1;括号为聚类稳健性标准误(Cluster-robust standard error);模型包含年份、行业和省份虚拟变量;样本量:1362。

(C)1994-2021 China Academic Journal Electronic Publishing House. All rights reserved. http://www.cnki.net

元合法性的效应增量取决于伦理型领导的取值大小，即伦理型领导取值越大，绿色工艺创新对适应合法性/战略合法性的影响增量越大。上述结果表明：在“层级文化”比较突出的中国，领导往往控制着企业的资源与发展方向，即企业是否引进新能源、新设备在很大程度上依赖于领导的决策（李锐、田晓明，2014）；而绿色工艺创新主要涉及使用清洁能源以及引进清洁设备等（Xie et al.，2016），因此伦理型领导在绿色工艺创新过程中会起到重要作用。对于绿色产品创新，表4的模型3和模型6结果显示，绿色产品创新与伦理型领导的交互项（*Prod*×*Ethi*）对适应合法性和战略合法性的影响均不显著（β_1=0.038，p_1>0.1；β_2=0.006，p_2>0.1）。因此，伦理型领导在绿色产品创新与适应合法性/战略合法性关系之间的调节作用不成立，即H3b和H3d未得到验证。

表5是基于伦理型领导的条件过程分析结果。结果表明，伦理型领导正向调节适应合法性在绿色工艺创新与可持续发展绩效之间的中介效应（β_1=0.044，CI_1=[0.014，0.077]；β_2=0.813，CI_2=[0.255，1.391]），因此H4a成立。但适应合法性在绿色产品创新与可持续发展绩效之间的中介效应并不依赖于伦理型领导取值的变化而改变（β_1=0.032，CI_1=[−0.008，0.073]；β_2=0.583，CI_2=[−0.148，1.329]），即H4b未得到验证。此外，表5结果显示，伦理型领导对战略合法性在绿色工艺创新与可持续发展绩效之间的中介效应具有调节作用（β_1=0.048，CI_1=[0.010，0.088]；β_2=1.008，CI_2=[0.212，1.821]），因此，H4c得到验证；但战略合法性在绿色产品创新与可持续发展绩效之间的中介效应不依赖于伦理型领导取值的变化而改变（β_1=0.004，CI_1=[−0.043，0.051]；β_2=0.085，CI_2=[−0.897，1.068]），即H4d不成立。

（四）Post-hoc分析

为进一步挖掘伦理型领导在绿色产品创新与二元合法性之间未能发挥调节作用的可能情境，本文进行Post-hoc检验。鉴于绿色补贴是政府为激励企业实施绿色创新活动以降低环境污染为宗旨而提供的环保激励政策（Xie et al.，2016），它既能在一定程度上弥补因“知识溢出”效应等导致的市场失灵，又能向外界传达企业的经营能力是与制度要求相匹配和值得认可的信号（Xie et al.，2016；杨洋等，2015），因此，本研究将全样本划分为有绿色补贴和无绿色补贴两个子样本，以进一步挖掘伦理型领导未能调节绿色产品创新与合法性关系的原因，分组回归结果见表6。

表6结果表明，当企业享有绿色补贴时，伦理型领导在绿色产品创新与二元合法性之间发挥正向调节作用（β_1=0.123，p_1<0.01；β_2=0.119，p_2<0.05）；然而，当企业未获得绿色补贴时，伦理型领导在绿色产品创新与二元合法性之间的调节作用不显著（β_1=0.018，p_1>0.1；β_2=−0.015，p_2>0.1）。因此，伦理型领导的调节效应在是否有绿色补贴的分组中存在差异。为进一步检验两组之间系数差异是否具有统计显著性，本研究进行了费舍尔组合检验，通过Bootstrap法得到的经验p值见表6下方：结果显示，经验p值在10%水平上显著，可见伦理型领导在绿色产

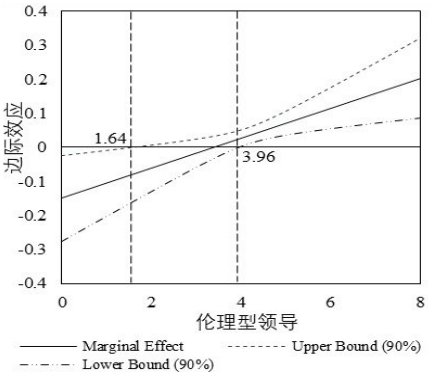

图2a 绿色工艺创新对适应合法性的影响增量变化图

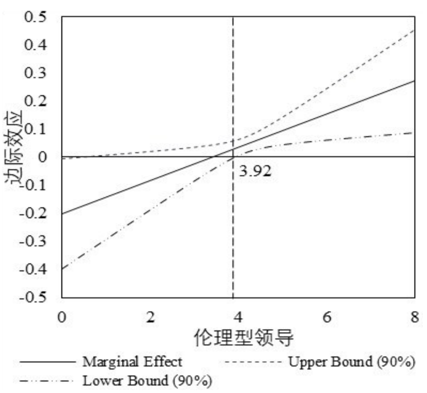

图2b 绿色工艺创新对战略合法性的影响增量变化图

表5 基于伦理型领导的条件过程分析结果：伦理型领导对中介路径的前端调节作用

Mediators（M）	Dependent Variable（Y）	Interactions（Int）	First stage Coeff($\hat{a}$)（*Int*→*M*）	Second stage Coeff($\hat{b}$)（*M*→*Y*）	Index of moderated mediation（Monte Carlo method）Index	90% CI LLCI	90% CI ULCI
Conforming legitimacy	*Financial performance*	*Proc*×*Ethi*	0.053** (0.022)	0.834*** (0.099)	0.044 (0.019)	0.014	0.077
		Prod×*Ethi*	0.038 (0.029)		0.032 (0.025)	−0.008	0.073
	Environmental performance	*Proc*×*Ethi*	0.053** (0.022)	15.347*** (1.299)	0.813 (0.346)	0.255	1.391
		Prod×*Ethi*	0.038 (0.029)		0.583 (0.449)	−0.148	1.329
Strategic legitimacy	*Financial performance*	*Proc*×*Ethi*	0.071** (0.034)	0.680*** (0.073)	0.048 (0.024)	0.010	0.088
		Prod×*Ethi*	0.006 (0.042)		0.004 (0.029)	−0.043	0.051
	Environmental performance	*Proc*×*Ethi*	0.071** (0.034)	14.195*** (1.020)	1.008 (0.489)	0.212	1.821
		Prod×*Ethi*	0.006 (0.042)		0.085 (0.598)	−0.897	1.068

注：***p<0.01，**p<0.05，*p<0.1；CI代表置信区间（Confidence Interval）；LL代表下限（Lower Limit）；UL代表上限（Upper Limit）；$\hat{a}$、$\hat{b}$分别表示a、b的估计量，而a、b分别代表交互项对中介、中介对因变量的真实参数；一般来说a，b相关性很小，不会影响效应显著检验结果，由此本研究令所有ρ_{ab}=0；括号为标准误。

(C)1994-2021 China Academic Journal Electronic Publishing House. All rights reserved.   http://www.cnki.net

品创新与二元合法性之间的调节作用在分样本中存在显著的异质性。其次,为了比较伦理型领导对“绿色产品创新—二元合法性—可持续发展绩效”逻辑过程的调节效应是否存在分组差异,本研究进行了条件过程分析。表7结果表明,当企业享有绿色补贴时,伦理型领导调节合法性在绿色产品创新和可持续发展绩效之间的中介效应;然而,当企业未获得绿色补贴时,伦理型领导未能显著调节合法性在绿色产品创新和可持续发展绩效之间的中介效应。该结果表明,与非伦理型领导相比,伦理型领导更愿意将绿色补贴投入到绿色创新实践,以降低产品研发中的“试错成本”,进而提高产品—市场匹配度(Product-market fit),从而改善企业合法性,提升企业的可持续发展绩效。因此,在获得绿色补贴的企业中,伦理型领导调节二元合法性在绿色产品创新与可持续发展绩效之间的中介效应。

此外,表6结果表明,绿色产品创新的积极作用并不存在显著的组别差异性(β_1=-0.031,p_1>0.1;β_2=-0.041,p_2>0.1)。该研究结果不仅拓展了Xie等(2019)研究结论——企业绿色产品创新的积极作用不能仅靠外部绿色补贴来强化;而且进一步表明:源于西方管理思想的绿色创新理论(健全政治生态为宗旨)尚无法与中国企业实践进行完美融合。该结果揭示,绿色产品创新作用的有效发挥需要嵌入本土“和谐共生”思想(例如,“道法自然”、“仁义礼智”)。目前,虽然政府基于环境规制积极推进绿色治理,但倘若没有以基于伦理道德的“和谐共生”文化作为根基,环保政策很难发挥实际效应。综上,该结果揭示,企业通过内外因素的有效融合——即绿色补贴与伦理型领导的联合效应,将有助于企业更好地通过绿色创新获得合法性和提升可持续发展绩效。

表6 Post-hoc分组检验结果

Variable	*Conforming legitimacy*		*Strategic legitimacy*	
	Subsidies=1	*Subsidies*=0	*Subsidies*=1	*Subsidies*=0
	模型1	模型2	模型3	模型4
Explanatory variable				
Prod	0.059*** (0.018)	0.090*** (0.026)	0.125*** (0.027)	0.166*** (0.033)
Moderator				
Ethi	0.002 (0.039)	0.036 (0.030)	0.004 (0.049)	0.067 (0.042)
Interaction				
Prod × Ethi	0.123*** (0.046)	0.018 (0.028)	0.119** (0.046)	−0.015 (0.042)
Controls				
Size	0.124 (0.098)	0.165* (0.098)	0.186 (0.147)	0.239 (0.182)
Numb	−0.082 (0.104)	0.010 (0.086)	−0.156 (0.158)	−0.100 (0.108)
Age	−0.198** (0.090)	−0.400*** (0.139)	−0.601*** (0.130)	−0.784*** (0.172)
Grow	0.155 (0.111)	−0.022 (0.079)	0.189 (0.204)	−0.115 (0.101)
ROS	0.062 (0.145)	−0.351* (0.205)	−0.146 (0.264)	0.199 (0.375)
Lev	0.190 (0.321)	0.066 (0.266)	−0.114 (0.529)	0.281 (0.448)
R&D	−0.850 (2.290)	1.009 (2.928)	−2.972 (4.260)	4.798 (3.538)
ISO	−0.131 (0.113)	−0.031 (0.085)	−0.208 (0.139)	−0.119 (0.153)
Inst	−0.002 (0.085)	−0.125 (0.080)	−0.105 (0.122)	−0.088 (0.106)
Dire	0.461 (0.381)	0.069 (0.370)	0.977* (0.508)	0.401 (0.496)
Supe	0.315** (0.129)	0.146 (0.088)	0.443*** (0.120)	0.473*** (0.132)
_Cons	1.119 (2.737)	2.449 (2.846)	5.469 (3.825)	4.887 (4.693)
R^2	0.091	0.142	0.139	0.211
F-value	9.900***	4.690***	25.460***	9.730***
N	735	627	735	627
Year dummies	Included	Included	Included	Included
Industry dummies	Included	Included	Included	Included
Province dummies	Included	Included	Included	Included
Differences				
$\beta_{Prod1}-\beta_{Prod0}$	−0.031		−0.041	
$\beta_{Interaction1}-\beta_{Interaction0}$	0.105		0.134	
p-values				
$p(\beta_{Prod1}-\beta_{Prod0})$	0.160		0.176	
$p(\beta_{Interaction1}-\beta_{Interaction0})$	0.036		0.020	

注:***p<0.01,**p<0.05,*p<0.1;括号为聚类稳健性准误(Cluster-robust standard error);模型包含年份、行业和省份虚拟变量,p值用于检验组间系数差异显著性,通过Bootstrap抽样1000次得到。

五、内生性与稳健性检验

(一)内生性检验

双向因果导致的内生性问题是本研究需要解决的重要内容,即绿色创新提高了企业合法性水平,而企业合法性反过来也有可能促进企业绿色创新能力的改善。为此,本文使用两阶段工具变量模型对绿色创新与二元合法性的直接效应做进一步检验。鉴于绿色工艺创新旨在从生产源头和末端减少污染物排放,提高能源利用率(Xie et al.,2019),因此,能源的价格机制是影响企业能源投入决策的重要因

表7 分组检验的条件过程分析结果

Mediators (M)	Dependent Variable (Y)	Interactions (Int)	Groups	First stage	Second stage	Index of moderated mediation (Monte Carlo method)		
			Subsidies (*dummies*)	Coeff($\hat{a}$) ($Int \rightarrow M$)	Coeff($\hat{b}$) ($M \rightarrow Y$)	Index	90% CI	
							LLCI	ULCI
Conforming legitimacy	*Financial performance*	*Prod × Ethi*	1	0.123*** (0.046)	0.843*** (0.133)	0.104 (0.043)	0.038	0.177
			0	0.018 (0.028)	0.860*** (0.157)	0.015 (0.025)	−0.024	0.057
	Environmental performance	*Prod × Ethi*	1	0.123*** (0.046)	15.220*** (1.860)	1.872 (0.742)	0.699	3.132
			0	0.018 (0.028)	16.412*** (1.909)	0.295 (0.464)	−0.459	1.064
Strategic legitimacy	*Financial performance*	*Prod × Ethi*	1	0.119** (0.046)	0.632*** (0.091)	0.075 (0.031)	0.026	0.129
			0	−0.015 (0.042)	0.773*** (0.107)	−0.012 (0.033)	−0.066	0.042
	Environmental performance	*Prod × Ethi*	1	0.119** (0.046)	14.316*** (1.349)	1.704 (0.681)	0.610	2.846
			0	−0.015 (0.042)	14.782*** (1.541)	−0.222 (0.625)	−1.252	0.800

注:***p<0.01,**p<0.05,*p<0.1;CI代表置信区间(Confidence Interval);LL代表下限(Lower Limit);UL代表上限(Upper Limit);$\hat{a}$、$\hat{b}$分别表示a、b的估计量,而a、b分别代表交互项对中介、中介对因变量的真实参数;一般来说,a、b相关性很小,不会影响效应显著检验结果,所以,本研究令所有ρab=0;括号为标准误。

(C)1994-2021 China Academic Journal Electronic Publishing House. All rights reserved. http://www.cnki.net

素。在本研究中,能源价格指数⑥是由石油价格指数、煤炭价格指数和天然气价格指数三者加权而得,能够综合反映能源市场状况以及能够作为企业采购与投资的标准;同时能源价格波动的异质性能够保证其具有较好的外生性。综上,能源价格指数能够较好地满足工具变量的“相关性”与“排他性”假设。因此,本文采用能源价格指数作为绿色工艺创新的工具变量。此外,以往文献表明,绿色产品创新强调基于绿色理念的产品设计与改进,以此降低产品在生命周期内的生态负效应(Chan et al.,2016);而绿色专利是以绿色技术为主题的发明和实用型专利,是企业对绿色产品或产品构造进行设计或改进的技术方案(陈思等,2017),并且绿色专利授权数量的异质性能够保证其具有较好的外生性。由此,绿色专利满足工具变量的“相关性”与“排他性”假设。因此,本文采用绿色专利作为绿色产品创新的工具变量。由表8可知,在考虑内生性问题后,绿色创新对二元合法性依然具有显著的正向作用;此外,结果表明,Cragg-Donald Wald F 统计值均大于10%的误差容忍临界值,且第一阶段工具变量系数均显著,因此,以能源价格指数和绿色专利作为绿色创新两个维度的工具变量是有效的。

此外,绿色创新能力高的企业往往具有浓厚的“仁义礼智”等伦理文化,这也会影响企业高管的伦理特质;而高管的决策又会影响绿色创新战略的实施,从而影响模型估计的一致性与无偏性。鉴于此,本文参考王超发等(2020)研究,采用内生变量替换法以缓解调节变量(伦理型领导)的内生性偏倚问题。参照Brown等(2005)以及曹洪军和陈泽文(2017)的研究,本文采用“高管是否重视对下属违反道德标准的行为予以规范与处罚”作为人文关怀导向伦理型领导的代理测度;采用“高管制定的预算计划是否包括环境投资与绿色采购等经营活动”作为环境可持续发展导向伦理型领导的代理变量。表9结果表明,基于代理变量的回归结果与上述研究结论无显著差异。此外,遗漏变量也有可能导致内生性问题,尽管本文已将许多控制变量纳入研究模型,但为进一步解决模型中潜在的内生性问题,本文采用财务绩效和环境社会责任绩效滞后一期进行动态模型估计,并且以内生变量的一阶和二阶滞后项为工具变量,采用系统广义矩估计法(Two-step SYS-GMM)来缓解模型的内生性问题。表10和表11的回归结果与上述研究结论依然一致,此外,Hansen检验结果证明系统GMM估计是有效的。因此,估计结果具是稳健性,即本研究结论得到进一步支持。

(二)稳健性检验

为进一步验证研究结果的可靠性,本研究采用替换变量进行稳健性检验。首先,采用双元绩效作为企业

表8 内生性检验:绿色创新与二元合法性两阶段工具变量模型估计结果(2SLS)

Variable	OLS model				2SLS model			
	Conforming legitimacy		*Strategic legitimacy*		*Conforming legitimacy*		*Strategic legitimacy*	
Explanatory variables	模型1	模型2	模型3	模型4	模型5	模型6	模型7	模型8
Proc	0.044^{***} (0.012)		0.071^{***} (0.015)		0.040^{**} (0.017)		0.069^{***} (0.023)	
Prod		0.073^{***} (0.013)		0.140^{***} (0.017)		0.066^{***} (0.021)		0.132^{***} (0.027)
Controls								
Size	0.188^{***} (0.060)	0.157^{***} (0.060)	0.306^{***} (0.080)	0.246^{***} (0.079)	0.188^{***} (0.060)	0.160^{***} (0.060)	0.307^{***} (0.079)	0.249^{***} (0.079)
Numb	−0.055 (0.060)	−0.042 (0.059)	-0.154^{*} (0.079)	-0.131^{*} (0.077)	−0.054 (0.059)	−0.043 (0.059)	-0.154^{**} (0.078)	-0.132^{*} (0.077)
Age	-0.407^{***} (0.124)	-0.340^{***} (0.124)	-0.801^{***} (0.165)	-0.681^{***} (0.161)	-0.404^{***} (0.124)	-0.343^{***} (0.123)	-0.799^{***} (0.164)	-0.685^{***} (0.161)
Growth	0.042 (0.065)	0.051 (0.065)	0.004 (0.086)	0.024 (0.084)	0.041 (0.065)	0.050 (0.064)	0.004 (0.085)	0.022 (0.084)
ROS	−0.171 (0.132)	−0.149 (0.132)	−0.028 (0.175)	0.017 (0.172)	−0.172 (0.131)	−0.152 (0.131)	−0.029 (0.174)	0.013 (0.171)
Lev	0.102 (0.179)	0.143 (0.178)	0.046 (0.237)	0.126 (0.233)	0.102 (0.178)	0.139 (0.177)	0.046 (0.235)	0.121 (0.231)
R&D	−0.073 (1.732)	0.008 (1.720)	0.786 (2.289)	1.010 (2.246)	−0.091 (1.720)	−0.018 (1.709)	0.775 (2.273)	0.977 (2.231)
ISO	−0.030 (0.081)	−0.054 (0.080)	−0.080 (0.107)	−0.119 (0.105)	−0.032 (0.081)	−0.054 (0.080)	−0.081 (0.107)	−0.119 (0.104)
Inst	-0.091^{*} (0.047)	−0.074 (0.047)	-0.137^{**} (0.062)	-0.106^{*} (0.061)	-0.091^{*} (0.047)	−0.075 (0.046)	-0.137^{**} (0.062)	-0.107^{*} (0.060)
Dire	0.211 (0.227)	0.257 (0.225)	0.643^{**} (0.299)	0.730^{**} (0.294)	0.212 (0.225)	0.254 (0.224)	0.643^{**} (0.297)	0.725^{**} (0.292)
Supe	0.250^{***} (0.063)	0.237^{***} (0.062)	0.461^{***} (0.083)	0.433^{***} (0.081)	0.250^{***} (0.062)	0.239^{***} (0.062)	0.461^{***} (0.082)	0.436^{***} (0.081)
Year dummies	Included	Included	Included	Included	Included	Included	Included	Included
Industry dummies	Included	Included	Included	Included	Included	Included	Included	Included
Province dummies	Included	Included	Included	Included	Included	Included	Included	Included
_Cons	4.410^{*} (2.532)	3.813 (2.510)	8.671^{***} (3.346)	7.702^{**} (3.278)	–	–	–	–
R^2	0.069	0.082	0.115	0.147	0.069	0.082	0.115	0.147
First stage IV								
Energy price index	–	–	–	–	4.387^{***}		4.387^{***}	
Green patent	–	–	–	–		8.567^{***}		8.567^{***}
Cragg-Donald Wald F	–	–	–	–	896.722	779.386	896.722	779.386
10% maximal IV size	–	–	–	–	16.380	16.380	16.380	16.380

注:***p<0.01,**p<0.05,*p<0.1;括号里为标准误;模型包含年份、行业和省份虚拟变量;样本量:1362。

(C)1994-2021 China Academic Journal Electronic Publishing House. All rights reserved. http://www.cnki.net

可持续发展绩效的替代变量进行稳健性分析。根据组织双元理论（Organization ambidexterity），本文的双元绩效代表了企业在资源有限的条件下，同时实现财务绩效和环境社会责任绩效的有效平衡和联合价值。具体测量步骤如下：将企业财务绩效和环境社会责任绩效分别进行0-1标准化，标准化公式为：$y^*=(y-min)/(max-min)$；借鉴Zang和Li（2017）的双元公式，本文将标准化后的企业财务绩效与环境社会责任绩效转化为双元绩效（Ambidextrous Performance，简记：Ambi），双元绩效公式为：$Ambi=[(1-|Fina-Envi|)\times\sqrt{Fina\times Envi}]/1$ 。表12的检

表9 调节变量的内生性检验：伦理型领导的代理变量

Variable	*Conforming legitimacy*			*Strategic legitimacy*		
Explanatory variables	模型1	模型2（*Subsidies* = 1）	模型3（*Subsidies* = 0）	模型4	模型5（*Subsidies* = 1）	模型6（*Subsidies* = 0）
Proc	0.025* (0.015)			0.034* (0.018)		
Prod	0.061*** (0.015)	0.061*** (0.017)	0.091*** (0.026)	0.126*** (0.022)	0.126*** (0.027)	0.168*** (0.033)
Moderator						
Ethi	0.039 (0.037)	0.015 (0.061)	0.059 (0.043)	0.057 (0.047)	0.004 (0.081)	0.099 (0.066)
Interactions						
Proc × *Ethi*	0.069** (0.034)			0.106** (0.051)		
Prod × *Ethi*	0.078 (0.049)	0.171** (0.072)	0.043 (0.052)	−0.001 (0.068)	0.142** (0.070)	−0.038 (0.084)
Controls						
Size	0.169*** (0.064)	0.114 (0.097)	0.160 (0.097)	0.264** (0.109)	0.178 (0.148)	0.234 (0.183)
Numb	−0.051 (0.063)	−0.077 (0.103)	0.006 (0.086)	−0.142 (0.093)	−0.152 (0.158)	−0.102 (0.106)
Age	−0.371*** (0.087)	−0.232** (0.090)	−0.401*** (0.139)	−0.729*** (0.113)	−0.636*** (0.134)	−0.765*** (0.170)
Growth	0.057 (0.068)	0.155 (0.112)	−0.016 (0.078)	0.027 (0.116)	0.187 (0.205)	−0.102 (0.103)
ROS	−0.136 (0.133)	0.067 (0.145)	−0.342 (0.206)	0.028 (0.235)	−0.139 (0.265)	0.204 (0.375)
Lev	0.147 (0.212)	0.198 (0.322)	0.068 (0.269)	0.130 (0.357)	−0.104 (0.533)	0.265 (0.447)
R&D	0.040 (1.934)	−0.784 (2.330)	1.222 (2.892)	0.993 (2.760)	−2.875 (4.242)	5.174 (3.568)
ISO	−0.041 (0.080)	−0.123 (0.110)	−0.032 (0.085)	−0.100 (0.111)	−0.203 (0.142)	−0.113 (0.159)
Inst	−0.088 (0.055)	−0.003 (0.085)	−0.123 (0.081)	−0.123 (0.076)	−0.107 (0.123)	−0.082 (0.106)
Dire	0.247 (0.267)	0.465 (0.382)	0.0585 (0.369)	0.722** (0.350)	0.984* (0.508)	0.411 (0.492)
Supe	0.245*** (0.080)	0.329** (0.127)	0.147* (0.086)	0.439*** (0.088)	0.456*** (0.120)	0.472*** (0.130)
_Cons	2.334 (1.873)	1.683 (2.731)	2.592 (2.829)	4.713 (2.865)	6.010 (3.880)	4.775 (4.771)
Year dummies	Included	Included	Included	Included	Included	Included
Industry dummies	Included	Included	Included	Included	Included	Included
Province dummies	Included	Included	Included	Included	Included	Included
R^2	0.099	0.090	0.143	0.160	0.136	0.210
F-value	9.030***	8.760***	4.800***	18.520***	23.330***	10.570***
Observations	1362	735	627	1362	735	627

注：***p<0.01，**p<0.05，*p<0.1；括号为聚类稳健性标准误（Cluster-robust standard error）；模型包含年份、行业和省份虚拟变量。

表10 内生性检验：绿色创新对可持续发展绩效系统广义矩估计结果（Two-step SYS-GMM）

Variable	*Financial performance*			*Environmental performance*		
Explanatory variables	模型1	模型2	模型3	模型4	模型5	模型6
L.Fina	0.199*** (0.073)	0.179*** (0.060)	0.141*** (0.051)			
L.Envi				0.579*** (0.063)	0.443*** (0.059)	0.288*** (0.051)
Proc	0.097* (0.057)	0.038 (0.056)	0.034 (0.056)	1.933*** (0.692)	1.738*** (0.658)	1.471** (0.654)
Prod	0.218*** (0.076)	0.209** (0.085)	0.128** (0.061)	1.898** (0.834)	1.200 (0.874)	0.374 (0.740)
Mediators						
Conf		0.917*** (0.151)			14.580*** (1.655)	
Stra			0.891*** (0.116)			16.975*** (2.029)
Controls						
Size	0.290* (0.163)	0.274** (0.139)	0.233* (0.122)	4.252*** (1.540)	4.023*** (1.543)	4.152** (2.019)
Numb	0.293* (0.163)	0.162 (0.169)	0.281** (0.139)	2.041 (1.862)	2.053 (1.839)	2.759 (1.783)
Age	−0.032 (0.024)	−0.017 (0.021)	−0.002 (0.020)	−0.261 (0.186)	0.092 (0.279)	0.230 (0.268)
Growth	0.838*** (0.266)	0.780*** (0.232)	0.727*** (0.237)	3.380 (3.012)	2.145 (3.033)	3.081 (3.324)
ROS	4.240*** (1.354)	4.316*** (1.307)	4.304*** (1.215)	6.252 (8.483)	7.003 (8.377)	5.137 (6.107)
Lev	−0.466 (0.823)	−0.394 (0.710)	−0.832 (0.687)	−3.575 (7.652)	0.202 (7.965)	−2.045 (8.229)
R&D	4.975 (3.891)	1.213 (3.922)	0.238 (4.265)	41.650 (42.230)	48.720 (52.540)	22.063 (65.49)
ISO	0.073 (0.345)	−0.206 (0.312)	−0.139 (0.272)	−3.066 (2.673)	−6.343* (3.613)	−10.754** (4.270)
Inst	−0.088** (0.038)	−0.070 (0.044)	−0.051 (0.042)	−0.600 (0.502)	−0.491 (0.614)	−0.254 (0.799)
Dire	2.725** (1.151)	2.164** (1.091)	2.248** (1.032)	21.06* (11.59)	10.220 (10.720)	2.236 (12.22)
Supe	0.404 (0.334)	0.457 (0.370)	0.246 (0.301)	3.198 (4.153)	5.067 (4.202)	0.884 (4.555)
_Cons	−5.566* (3.327)	−5.217 (3.727)	−5.224* (3.006)	−104.300*** (38.550)	−114.000*** (38.370)	−115.039** (45.543)
Year dummies	Included	Included	Included	Included	Included	Included
Industry dummies	Included	Included	Included	Included	Included	Included
Province dummies	Included	Included	Included	Included	Included	Included
Wald chi2	319.240***	353.370***	387.940***	392.060***	396.420***	250.850***
AR(1)	−4.360 (0.000)	−4.680 (0.000)	−4.700 (0.000)	−6.070 (0.000)	−5.900 (0.000)	−5.510 (0.000)
AR(2)	1.060 (0.291)	1.140 (0.255)	0.870 (0.384)	2.140 (0.032)	2.330 (0.020)	2.120 (0.034)
Hansen test	173.550 (0.134)	186.570 (0.120)	177.670 (0.237)	159.330 (0.368)	180.500 (0.194)	174.550 (0.290)

注：***p<0.01，**p<0.05，*p<0.1；AR(1)、AR(2)和Hansen test括号为prob>z的值；其他指标括号为聚类稳健性准误（Cluster-robust standard error）；模型包含年份、行业和省份虚拟变量；样本量：1135。

(C)1994-2021 China Academic Journal Electronic Publishing House. All rights reserved. http://www.cnki.net

验结果表明,绿色创新的两个维度($β_1$=0.046,p_1<0.01;$β_2$=0.051,p_2<0.01)、二元合法性两个维度($β_1$=0.312,p_1<0.01;$β_2$=0.264,p_2<0.01)对双元绩效均具有显著的正向影响。

其次,本研究采用战略合法性的替代变量进行稳健性分析。基于已有研究(Tornikoski and Newbert,2007),创造一个可信赖的组织印象是企业获取战略合法性的重要措施,因此,本文采用“公司是否获得“国家环境友好型企业”称号”衡量战略合法性,并进行Logistic回归。表12结果表明,绿色创新的两个维度($β_1$=0.185,p_1<0.05;$β_2$=0.430,p_2<0.01)均能显著提升企业战略合法性水平。此外,由于中介变量是二分类变量,传统的中介分析技术会产生偏差,为了验证中介效应的稳健性,我们根据Iacobucci(2012)的建议,使用蒙特卡罗法(MC)进行中介效应的点估计和区间估计。表13列出了中介效应的点估计和不对称置信区间的估计结果,结果表明二元合法性的中介作用依然成立。同时,表14给出了条件过程分析的稳健性检验结果,结果表明,采用双元绩效作为可持续发展绩效的替换变量以及替换战略合法性测度后并未改变本文的研究结论。综上,本文的研究结论具有较好的可靠性。

六、结论与启示

(一)理论启示

在制度与经济转型背景下,我国制造业企业的绿色创新实践面临“双重悖论”:一方面,企业面临日趋严格的环境规制;另一方面,很多重污染企业仍然尚未积极履行环境社会责任,而悖论的根源是忽略了绿色创新的有效实施需要嵌入本土的“和谐共生”理念。据此,本研究将“德法并重”观念引入“绿色创新—二元合法性—可持续发展绩效”的逻辑框架,构建了一个条件过程模型,深入探究在伦理型领导边界条件下,二元合法性在绿色创新与可持续发展绩效关系中间接效应的边际变化情况,以期深层次揭示绿色创新获益的内在机理;并

表11 内生性检验:绿色创新对合法性边际效应的系统广义矩估计结果(Two-step SYS-GMM)

Variable	*Conforming legitimacy*	*Strategic legitimacy*	*Conforming legitimacy*		*Strategic legitimacy*	
			Subsidies = 1	*Subsidies* = 0	*Subsidies* = 1	*Subsidies* = 0
Explanatory variables	模型1	模型2	模型3	模型4	模型5	模型6
L.Conf	0.337*** (0.052)		0.118* (0.070)	0.152* (0.079)		
L.Stra		0.416*** (0.052)			0.128* (0.075)	0.148 (0.101)
Proc	0.044*** (0.017)	0.060*** (0.019)				
Prod			0.074** (0.033)	0.108*** (0.038)	0.138*** (0.036)	0.107** (0.045)
Moderator						
Ethi	0.008 (0.028)	0.067** (0.029)	−0.012 (0.056)	0.031 (0.035)	0.038 (0.050)	0.0831** (0.041)
Interactions						
Proc × Ethi	0.050* (0.030)	0.053** (0.027)				
Prod × Ethi			0.101* (0.059)	0.018 (0.043)	0.145* (0.077)	0.028 (0.053)
Controls						
Size	0.061 (0.039)	0.098** (0.043)	0.063 (0.082)	0.116 (0.084)	−0.061 (0.107)	−0.025 (0.062)
Numb	−0.012 (0.037)	−0.025 (0.037)	0.031 (0.099)	−0.099 (0.076)	−0.121 (0.114)	−0.069 (0.088)
Age	−0.018*** (0.007)	−0.021*** (0.007)	−0.020 (0.018)	−0.046*** (0.016)	−0.013 (0.015)	−0.026* (0.014)
Growth	0.037 (0.089)	−0.020 (0.102)	0.301** (0.151)	−0.123 (0.120)	0.178 (0.152)	−0.160 (0.115)
ROS	0.111 (0.163)	0.446 (0.283)	−0.037 (0.151)	−0.461 (0.410)	0.240 (0.478)	0.591 (0.451)
Lev	−0.141 (0.146)	0.067 (0.209)	−0.501 (0.333)	−0.189 (0.411)	0.393 (0.520)	0.572** (0.286)
R&D	0.662 (1.970)	−0.711 (1.847)	0.330 (4.243)	1.557 (2.824)	2.100 (4.018)	5.294* (2.984)
ISO	0.147* (0.078)	0.139* (0.077)	0.242 (0.167)	0.201 (0.197)	0.0412 (0.147)	0.092 (0.221)
Inst	−0.010 (0.012)	−0.015 (0.014)	−0.086 (0.064)	−0.013 (0.053)	−0.119* (0.067)	−0.048 (0.057)
Dire	0.501 (0.310)	0.522 (0.400)	0.416 (0.575)	0.771 (0.521)	0.840 (0.552)	0.490 (0.529)
Supe	−0.042 (0.126)	0.252* (0.135)	0.275 (0.251)	0.136 (0.274)	0.899*** (0.309)	0.472 (0.311)
_Cons	−0.284 (0.891)	−1.179 (0.961)	0.297 (2.416)	−0.378 (1.860)	3.880 (2.668)	2.161 (1.696)
Year dummies	Included	Included	Included	Included	Included	Included
Industry dummies	Included	Included	Included	Included	Included	Included
Province dummies	Included	Included	Included	Included	Included	Included
Wald chi2	248.720***	226.050***	32.440***	81.210***	79.550***	110.48***
N	1135	1135	612	523	612	523
AR(1)	−7.390 (0.000)	−6.830 (0.000)	−4.680 (0.000)	−4.720 (0.000)	−4.810 (0.000)	−3.170 (0.002)
AR(2)	2.720 (0.007)	1.810 (0.071)	0.980 (0.329)	2.090 (0.036)	1.400 (0.161)	1.200 (0.231)
Hansen test	209.900 (0.301)	190.670 (0.706)	92.980 (0.152)	88.070 (0.251)	69.550 (0.742)	69.510 (0.743)

注:***p<0.01,**p<0.05,*p<0.1;AR(1)、AR(2)和Hansen test括号为prob>z的值;其他指标括号为聚类稳健性准误(Cluster-robust standard error);模型包含年份、行业和省份虚拟变量;样本量:1135。

(C)1994-2021 China Academic Journal Electronic Publishing House. All rights reserved. http://www.cnki.net

表12 稳健性检验:替换核心变量(绿色创新对可持续发展绩效直接效应检验结果)

Variable	*Ambidextrous performance*(*Robust test* 1)				*Strategic legitimacy*(*Robust test* 2)		
	Static FE model				*Logit FE model*		
	模型1	模型2	模型3	模型4	模型5	模型6	模型7
Explanatory variables							
Proc		0.046^{***} (0.009)	0.038^{***} (0.008)	0.037^{***} (0.008)		0.185^{**} (0.073)	0.181^{**} (0.0760)
Prod		0.051^{***} (0.011)	0.032^{***} (0.010)	0.018^{*} (0.009)		0.430^{***} (0.092)	0.421^{***} (0.095)
Moderator							
Ethi							0.104 (0.111)
Interactions							
Proc×Ethi							0.409^{***} (0.130)
Prod×Ethi							0.147 (0.158)
Mediators							
Conf			0.312^{***} (0.021)				
Stra				0.264^{***} (0.015)			
Controls							
Size	0.120^{***} (0.044)	0.093^{**} (0.041)	0.043 (0.034)	0.027 (0.033)	0.583^{*} (0.324)	0.431 (0.330)	0.560^{*} (0.336)
Numb	0.030 (0.042)	0.031 (0.039)	0.045 (0.038)	0.067^{*} (0.034)	0.513 (0.350)	0.663^{*} (0.368)	0.688^{*} (0.392)
Age	-0.163^{***} (0.051)	-0.168^{***} (0.052)	−0.055 (0.039)	0.020 (0.044)	−0.736 (0.768)	−0.838 (0.818)	−1.199 (0.922)
Growth	0.110^{**} (0.053)	0.125^{**} (0.053)	0.109^{**} (0.043)	0.118^{***} (0.038)	-0.741^{**} (0.352)	-0.645^{*} (0.361)	-0.683^{*} (0.369)
ROS	0.549^{***} (0.154)	0.585^{***} (0.160)	0.631^{***} (0.162)	0.580^{***} (0.135)	−0.246 (0.621)	−0.003 (0.630)	−0.007 (0.635)
Lev	−0.153 (0.149)	−0.114 (0.153)	−0.157 (0.136)	−0.146 (0.105)	0.452 (0.918)	0.761 (0.944)	0.936 (0.962)
R&D	1.025 (1.182)	1.467 (1.164)	1.436 (1.083)	1.167 (0.937)	−5.399 (9.497)	−4.885 (9.704)	−5.537 (9.744)
ISO	−0.061 (0.072)	−0.034 (0.070)	−0.022 (0.066)	−0.008 (0.058)	−0.579 (0.467)	−0.359 (0.468)	−0.406 (0.472)
Inst	-0.071^{*} (0.038)	-0.072^{**} (0.035)	−0.047 (0.030)	−0.042 (0.030)	−0.128 (0.253)	−0.070 (0.261)	−0.160 (0.269)
Dire	0.295^{*} (0.177)	0.317^{*} (0.171)	0.240^{*} (0.138)	0.128 (0.138)	2.931^{**} (1.299)	3.529^{***} (1.337)	3.769^{***} (1.346)
Supe	0.211^{***} (0.047)	0.187^{***} (0.046)	0.114^{***} (0.039)	0.074^{*} (0.041)	0.576^{*} (0.346)	0.423 (0.357)	0.448 (0.363)
_Cons	−0.459 (1.245)	0.129 (1.160)	−0.602 (0.939)	−1.119 (0.949)	–	–	–
Year dummies	Included	Included	Included	Included	Included	Included	Included
Industry dummies	Included	Included	Included	Included	Included	Included	Included
Province dummies	Included	Included	Included	Included	Included	Included	Included
R^2	0.122	0.186	0.352	0.389	–	–	–
F-value	10.110^{***}	12.200^{***}	40.240^{***}	46.390^{***}	–	–	–
Log likelihood	–	–	–	–	−383.420	−362.042	−353.319
LR chi2	–	–	–	–	35.550^{***}	78.310^{***}	95.760^{***}

注:***p<0.01,**p<0.05,*p<0.1;Robust test 1括号为聚类稳健性准误(Cluster-robust standard error);Robust test 2括号为标准误;模型包含年份、行业和省份虚拟变量;样本量:1362。

表13 稳健性检验:替换核心变量(基于蒙特卡罗法的中介效应点估计和区间估计结果)

Conditions	Mediator (M)	Dependent Variables (Y)	Independent Variables (X)	First Stage	Second Stage	The indirect effect of X on Y(Monte Carlo method)		
				Coeff($\hat{a}$) ($X \rightarrow M$)	Coeff($\hat{b}$) ($M \rightarrow Y$)	Mediation	90% CI	
							LLCI	ULCI
Robust test 1 (*Mediation* = $\hat{a}\times\hat{b}$)	*Conforming legitimacy*	*Ambidextrous performance*	*Proc*	0.026^{*} (0.015)	0.312^{***} (0.021)	0.008 (0.004)	0.001	0.016
			Prod	0.063^{***} (0.016)		0.020 (0.005)	0.011	0.028
	Strategic legitimacy		*Proc*	0.035^{**} (0.018)	0.264^{***} (0.015)	0.009 (0.005)	0.001	0.017
			Prod	0.127^{***} (0.022)		0.034 (0.006)	0.024	0.044
Robust test 2 (*Mediation* = $Z_a\times Z_b$)	*Strategic legitimacy*	*Financial performance*	*Proc*	0.185^{**} (0.073)	0.467^{***} (0.096)	12.328 (2.211)	0.028	0.155
			Prod	0.430^{***} (0.092)		22.737 (3.334)	0.110	0.307
		Environmental performance	*Proc*	0.185^{**} (0.073)	9.678^{***} (1.058)	23.182 (2.429)	0.615	3.035
			Prod	0.430^{***} (0.092)		42.754 (4.142)	2.580	5.878

注:***p<0.01,**p<0.05,*p<0.1;CI代表置信区间(Confidence Interval);LL代表下限(Lower Limit);UL代表上限(Upper Limit);$Z_a=\hat{a}/\sigma(\hat{a})$;$Z_b=\hat{b}/\sigma(\hat{b})$;$Z=\frac{Z_a\times Z_b}{\sqrt{Z_a^2+Z_b^2+1}}$;Robust test 2的Mediation括号里为Z,其他括号为标准误。

表14 稳健性检验:替换核心变量(条件过程分析的稳健性检验结果)

Conditions	Mediators (M)	Dependent Variables (Y)	Interactions (Int)	First Stage	Second Stage	Index of moderated mediation(Monte Carlo method)		
				Coeff($\hat{a}$) ($Int \rightarrow M$)	Coeff($\hat{b}$) ($M \rightarrow Y$)	Index	90% CI	
							LLCI	ULCI
Robust test 1 (*Index*=$\hat{a}\times\hat{b}$)	*Conforming legitimacy*	*Ambidextrous performance*	*Proc×Ethi*	0.053^{**} (0.022)	0.311^{**} (0.021)	0.016 (0.007)	0.005	0.028
			Prod×Ethi	0.038 (0.029)		0.012 (0.009)	−0.003	0.027
	Strategic legitimacy		*Proc×Ethi*	0.071^{**} (0.034)	0.264^{***} (0.015)	0.019 (0.009)	0.004	0.034
			Prod×Ethi	0.006 (0.042)		0.002 (0.011)	−0.017	0.020
Robust test 2 (*Index*=$Z_a\times Z_b$)	*Strategic legitimacy*	*Financial performance*	*Proc × Ethi*	0.409^{***} (0.130)	0.469^{***} (0.097)	15.212 (2.598)	0.081	0.322
			Prod × Ethi	0.147 (0.158)		4.498 (0.895)	−0.052	0.022
		Environmental performance	*Proc × Ethi*	0.409^{***} (0.130)	9.512^{***} (1.067)	28.047 (2.950)	1.802	6.133
			Prod × Ethi	0.147 (0.158)		8.294 (0.920)	−1.068	3.926

注:***p<0.01,**p<0.05,*p<0.1;CI代表置信区间(Confidence Interval);LL代表下限(Lower Limit);UL代表上限(Upper Limit);Robust test 2的index括号里为Z,其他括号为标准误。

(C)1994-2021 China Academic Journal Electronic Publishing House. All rights reserved. http://www.cnki.net

依据企业是否享有绿色补贴进行Post-hoc分析，采用多种内生性与稳健性检验策略提高模型估计的无偏性与一致性，主要得到如下结论与理论启示。

第一，本研究从可持续发展绩效视角拓展了绿色创新对企业绩效的作用机制研究。以往研究主要聚焦单一企业绩效，例如：财务绩效（Xie et al.，2016）和环境绩效（Seman et al.，2019），本研究系统诠释了绿色创新对企业财务绩效与环境社会责任绩效的综合影响，进一步拓展了绿色创新与企业绩效关系研究的范畴。其次，以往研究多聚焦绿色创新的整体效应，较少比较不同类型的绿色创新对企业绩效的影响，本研究将绿色创新划分为绿色工艺创新和绿色产品创新两个维度，从更加微观的视角深层次比较不同维度的绿色创新对企业财务绩效和环境社会责任绩效作用机制的差异。研究发现：与绿色工艺创新相比，绿色产品创新对财务绩效的作用更显著；与绿色产品创新相比，绿色工艺创新对环境社会责任绩效的作用更显著。由此，该结论延伸了Huang和Li（2017）关于不同类型绿色创新对企业绩效影响的研究，拓展了绿色创新的理论外延。

第二，本研究基于“制度逻辑”与“效率逻辑”的辩证视角，提出“绿色创新—二元合法性—企业绩效”的研究框架，通过将二元合法性理论、绿色创新与可持续发展绩效进行有机耦合，弥补了当前关于绿色创新与可持续发展绩效关系之间“理论黑箱”研究不足的局限。以往绿色创新研究主要从“适应合法性”视角来解释企业被动实施绿色创新的驱动机制（El-Kassar and Singh，2019；Li et al.，2018），尚未揭示绿色创新对企业“战略合法性”获取的作用机制。本研究根植于本土“和谐理论”中的“和谐机制”（行为趋同与优化设计），从适应合法性和战略合法性理论融合的全新视角，深入诠释了二元合法性在绿色创新与可持续发展绩效关系之间的传导作用，为理解绿色创新与可持续发展之间的内部过程提供了独特的理论视角，进一步揭示了在制度和经济转型背景下合法性在企业绿色创新实施过程中的复杂作用，由此，拓展了二元合法性理论在绿色创新领域的应用研究。

第三，本研究进一步探究了伦理型领导在绿色创新经由合法性影响企业可持续发展绩效过程中的边界机制。现有关于伦理型领导的研究主要围绕管理者诚实、利他、可信赖等特质如何影响员工个体和团队的创造力（Mo et al.，2019；Tu and Lu，2013）以及如何改善企业文化（Pasricha et al.，2018）等方面展开，较少关注伦理型领导在绿色创新领域的研究。本研究立足本土“和谐共生”思想中的“德法并重”准则，将伦理型领导纳入“绿色创新—二元合法性—可持续发展绩效”的逻辑框架。研究发现，伦理型领导在绿色产品创新与战略合法性之间的调节效应不显著，为此，本文以企业是否享有绿色补贴展开Post-hoc分析，结果揭示，二元合法性在绿色产品创新和可持续发展绩效之间的中介效应在企业获得绿色补贴的情境下因领导的伦理特质而变化；但绿色产品创新的积极作用并不存在显著的组别差异。该结论揭示了在“宽猛相济”的儒家文化背景下，当环境规制难以规范时，伦理道德可以发挥有效的补充作用，不仅响应了Fu等（2020）提出“在可持续发展战略中，领导个体差异能够影响企业做出有益活动的程度”的观点，而且突破了以往绿色创新研究主要关注知识基础观（Xie et al.，2019）、资源基础观（El-Kassar and Singh，2019）、动态能力理论（Yu et al.，2017）等理论视角，由此，提高了合法性理论与领导行为理论的融合研究在绿色创新研究中的适应性。

（二）实践启示

本研究的实践启示如下：第一，企业应高度重视绿色创新战略对可持续发展的重要性，积极将绿色发展理念融入工艺改进和产品设计过程，不断改善绿色生产流程和提高绿色产品质量。此外，鉴于绿色创新能力提升的过程也是企业绿色创新实践从“浅绿色”过渡到“深绿色”的过程，企业应根据自身战略导向合理权衡资源投入，并根据绿色工艺创新和绿色产品创新的技术内涵差异，优化配置绿色创新的战略组合，采取科学合理的绿色创新战略决策，从而保证绿色创新实践的有效开展。第二，企业应充分认识到将绿色创新、二元合法性、伦理型领导以及可持续发展绩效同时纳入到企业绩效管理框架的科学性，积极树立良好的企业形象以构建值得信赖的关系网络，建立健全与政府、顾客等利益相关者的沟通渠道，努力识别社会参与者对企业可持续发展的认知要求与期待。此外，企业应采用伦理道德与环境规制高度融合的绿色创新战略，努力将“和谐共生”文化融入到企业绿色文化，培养并任命具有人文主义和环保主义的伦理型领导者，并且鼓励他们将这种观点传播给下属员工（Wang et al.，2017），促使员工自觉维护和关注生态环境，实现绿色组织认同，从而为开展绿色

(C)1994-2021 China Academic Journal Electronic Publishing House. All rights reserved. http://www.cnki.net

创新创造良好的伦理文化氛围。第三，对政策制定者而言，在提倡“德法并重”的中国绿色生态治理观念下，政府需要意识到绿色补贴等环境激励政策的有效落实离不开企业领导者环境伦理道德的培养与提高。因此，政府应将企业领导者的伦理道德评估纳入绿色补贴等激励政策的分配过程。此外，环境友好型企业并不是与生俱来的，它需要一段从观念意识转变（浅绿色创新）过渡到行为惯性（深绿色创新）的长期过程。因此，除了环境法规制约途径，政府还应通过政企合作等方式引导企业家积极参与各类环保协会与绿色管理培训，帮助企业确立并强化“和谐共生”的社会责任意识；同时，政府还应制定绿色采购、绿色园区以及绿色债券等多阶段、多类型的激励政策，不断激发企业投入更多资源长期从事绿色创新，从而促使企业形成积极履行环境社会责任的主动行为。

（三）局限与展望

本研究仍存在一些局限需在未来研究中深入探讨。首先，本研究仅探究了绿色工艺创新与绿色产品创新对企业可持续发展绩效的差异化影响，后续研究可以在此基础上引入绿色管理创新、绿色营销创新等维度或“浅绿色”、“中绿色”和“深绿色”等不同的绿色战略定位，以完善本文的研究范畴。其次，限于篇幅和复杂性，本研究仅从伦理型领导和绿色补贴视角挖掘绿色创新的边界条件，而在实践中还包含其他一些重要权变因素会影响绿色创新和可持续发展绩效之间的关系。由此，后续研究可以探究影响绿色创新与可持续发展绩效关系的其他内外权变因素，例如，外部因素——绿色认证（Li et al.，2018）、绿色用户参与（Sopjani et al.，2019）；内部因素——绿色形象（Xie et al.，2019）、绿色培训（Pinzone et al.，2019）等，以进一步探索企业通过绿色创新实现合法性和可持续发展的边界条件。最后，本研究仅以制造商为核心企业来考察绿色创新实践情况，在企业日益嵌入创新生态系统的背景下，后续研究可以尝试引入不同成员的绿色创新实践数据（如供销商、客户等），从而使研究结果更加符合企业的绿色创新实践。总之，希望本研究能够激发更多学者和管理者对绿色创新如何影响合法性和可持续发展绩效问题进行深入探讨，以期进一步推进我们的研究结论。

（作者单位：解学梅，同济大学经济与管理学院；朱琪玮，上海大学管理学院）

注释

①参考马连福等（2015）和俞红海等（2010）研究，以历年各地区市场化总指数的平均增长幅度作为2016~2017年和2017~2018年的增长幅度来获取2017和2018年各地区市场化总指数的数据。

②依据国民经济行业分类和代码（GB/T 4754-2017）将本文样本划分为17个制造业子行业。

③标准化回归系数为：未标准化回归系数×该自变量的标准差/因变量的标准差。

④即：0.074=0.071×2.027/1.955；0.301=0.370×1.589/1.955。

⑤即：0.187=2.395×2.027/25.989；0.181=2.969×1.589/25.989。

⑥能源价格指数说明与数据来源：http://index.sci99.com/channel/product/hy/%E8%83%BD%E6%BA%90/1.html。

参考文献

（1）曹洪军、陈泽文：《内外环境对企业绿色创新战略的驱动效应——高管环保意识的调节作用》，《南开管理评论》，2017年第6期。

（2）陈思、何文龙、张然：《风险投资与企业创新：影响和潜在机制》，《管理世界》，2017年第1期。

（3）董静、汪江平、翟海燕、汪立：《服务还是监控：风险投资机构对创业企业的管理——行业专长与不确定性的视角》，《管理世界》，2017年第6期。

（4）冯天丽、井润田：《制度环境与私营企业家政治联系意愿的实证研究》，《管理世界》，2009年第8期。

（5）郭海、沈睿、王栋晗、陈叙同：《组织合法性对企业成长的“双刃剑”效应研究》，《南开管理评论》，2018年第5期。

（6）贾兴平、刘益：《外部环境、内部资源与企业社会责任》，《南开管理评论》，2014年第6期。

（7）李锐、田晓明：《主管威权领导与下属前瞻行为：一个被中介的调节模型构建与检验》，《心理学报》，2014年第11期。

（8）李维安、张耀伟、郑敏娜、李晓琳、崔光耀、李惠：《中国上市公司绿色治理及其评价研究》，《管理世界》，2019年第5期。

（9）马连福、王丽丽、张琦：《混合所有制的优序选择：市场的逻辑》，《中国工业经济》，2015年第7期。

（10）任起顺：《和谐共生：中华思想原典笺评》，上海百家出版社，2009年。

（11）邵帅、吕长江：《实际控制人直接持股可以提升公司价值吗？——来自中国民营上市公司的证据》，《管理世界》，2015年第5期。

（12）王彩明、李健：《中国区域绿色创新绩效评价及其时空差异分析——基于2005~2015年的省际工业企业面板数据》，《科研管理》，2019年第6期。

（13）王超发、史思雨、杨德林：《沉淀资源、股权结构与企业研发产出效果》，《科学学研究》，2020年第6期。

（14）魏江、王丁、刘洋：《来源国劣势与合法化战略——新兴经济企业跨国并购的案例研究》，《管理世界》，2020年第3期。

（15）席酉民、熊畅、刘鹏：《和谐管理理论及其应用述评》，《管理世界》，2020年第2期。

（16）徐建中、贯君、林艳：《制度压力、高管环保意识与企业绿色创新实践——基于新制度主义理论和高阶理论视角》，《管理评

(C)1994-2021 China Academic Journal Electronic Publishing House. All rights reserved. http://www.cnki.net

论》,2017年第9期。
(17)杨兴全、吴昊旻、曾义:《公司治理与现金持有竞争效应——基于资本投资中介效应的实证研究》,《中国工业经济》,2015年第1期。
(18)杨洋、魏江、罗来军:《谁在利用政府补贴进行创新?——所有制和要素市场扭曲的联合调节效应》,《管理世界》,2015年第1期。
(19)叶陈刚、王孜、武剑锋、李惠:《外部治理、环境信息披露与股权融资成本》,《南开管理评论》,2015年第5期。
(20)俞红海、徐龙炳、陈百助:《终极控股股东控制权与自由现金流过度投资》,《经济研究》,2010年第8期。
(21)赵云辉、张哲、冯泰文、陶克涛:《大数据发展、制度环境与政府治理效率》,《管理世界》,2019年第11期。
(22)仲理峰、孟杰、高蕾:《道德领导对员工创新绩效的影响:社会交换的中介作用和权力距离取向的调节作用》,《管理世界》,2019年第5期。
(23)周方召、戴亦捷:《环境责任、技术创新与公司绩效——来自中国上市公司的证据》,《环境经济研究》,2020年第1期。
(24)宗计川、吕源、唐方方:《环境态度、支付意愿与产品环境溢价——实验室研究证据》,《南开管理评论》,2014年第2期。
(25)Albertini, E., 2014, "A Descriptive Analysis of Environmental Disclosure: A Longitudinal Study of French Companies", *Journal of Business Ethics*, Vol.121(2), pp.233~254.
(26)Arfi, W. B., Hikkerova, L. and Sahut, J., 2017, "External Knowledge Sources, Green Innovation and Performance", *Technological Forecasting and Social Change*, Vol.129, pp.210~220.
(27)Ashforth, B. E. and Gibbs, B. W., 1990, "The Double-edge of Organizational Legitimation", *Organization Science*, Vol.1(2), pp.177~194.
(28)Brown, M. E., Treviño, L. K. and Harrison, D. A., 2005, "Ethical Leadership: A Social Learning Perspective for Construct Development and Testing", *Organizational Behavior and Human Decision Processes*, Vol.97(2), pp.117~134.
(29)Chan, H. K., Yee, R. W. Y., Dai, J. and Lim, M. K., 2016, "The Moderating Effect of Environmental Dynamism on Green Product Innovation And Performance", *International Journal of Production Economics*, Vol.181, pp.384~391.
(30)Chen, Y. S., Lai, S. B. and Wen, C. T., 2006, "The Influence of Green Innovation Performance on Corporate Advantage in Taiwan", *Journal of Business Ethics*, Vol.67(4), pp.331~339.
(31)Chiou, T. Y., Chan, H. K., Lettice, F. and Chung, S. H., 2011, "The Influence of Greening the Suppliers and Green Innovation on Environmental Performance and Competitive Advantage in Taiwan", *Transportation Research Part E: Logistics and Transportation Review*, Vol.47(6), pp.822~836.
(32)Czinkota, M., Kaufmann, H. R. and Basile, G., 2014, "The Relationship between Legitimacy, Reputation, Sustainability and Branding for Companies and Their Supply Chains", *Industrial Marketing Management*, Vol.43(1), pp.91~101.
(33)DiMaggio, P. J., and Powell, W. W., 1983, "The Iron Cage Revisited: Institutional Isomorphism and Collective Rationality in Organizational Fields", *American Sociological Review*, Vol.48(2), pp.147~160.
(34)Driessen, P. H., Hillebrand, B., Kok, R. A. W. and Verhallen, T. M. M., 2013, "Green New Product Development: The Pivotal Role of Product Greenness", *IEEE Transactions on Engineering Management*, Vol.60(2), pp.315~326.
(35)El-Kassar, A. N. and Singh, S. K., 2019, "Green Innovation and Organizational Performance: The Influence of Big Data and the Moderating Role of Management Commitment and HR Practices", *Technological Forecasting and Social Change*, Vol.144, pp.483~498.
(36)Enderle, G., 1987, "Some Perspectives of Managerial Ethical Leadership", *Journal of Business Ethics*, Vol.6(8), pp.657~663.
(37)Fernando, Y., Jabbour, C. J. C. and Wah, W. X., 2019, "Pursuing Green Growth in Technology Firms Through the Connections Between Environmental Innovation and Sustainable Business Performance: Does Service Capability Matter?", *Resources, Conservation and Recycling*, Vol.141, pp.8~20.
(38)Fu, R., Tang, Y. and Chen, G., 2020, "Chief Sustainability Officers and Corporate Social (Ir) Responsibility", *Strategic Management Journal*, Vol.41(4), pp.656~680.
(39)Hojnik, J. and Ruzzier, M., 2016, "The Driving Forces of Process Eco-innovation and Its Impact on Performance: Insights from Slovenia", *Journal of Cleaner Production*, Vol.133, pp.812~825.
(40)Huang, J. W. and Li, Y. H., 2017, "Green Innovation and Performance: The View of Organizational Capability and Social Reciprocity", *Journal of Business Ethics*, Vol.145(2), pp.309~324.
(41)Iacobucci, D., 2012, "Mediation Analysis and Categorical Variables: The Final Frontier", *Journal of Consumer Psychology*, Vol.22(4), pp.582~594.
(42)Ilias, A., Kostas, K. and Dimitris, T., 2018, "Environmental and Financial Performance. Is There a Win-win or a Win-loss Situation? Evidence From the Greek Manufacturing", *Journal of Cleaner Production*, Vol.197, pp.1275~1283.
(43)Jones, S. A., Michelfelder, D. and Nair, I., 2017, "Engineering Managers and Sustainable Systems: The Need for and Challenges of Using an Ethical Framework for Transformative Leadership", *Journal of Cleaner Production*, Vol.140, pp.205~212.
(44)Krippendorff, K., 2018, *Content Analysis: An Introduction to Its Methodology*, Los Angeles: Sage Publications.
(45)Li, D., Huang, M., Ren, S., Chen, X. and Ning, L., 2018, "Environmental Legitimacy, Green Innovation, and Corporate Carbon Disclosure: Evidence from CDP China 100", *Journal of Business Ethics*, Vol.150(4), pp.1089~1104.
(46)Li, G., Wang, X., Su, S., and Su, Y., 2019, "How Green Technological Innovation Ability Influences Enterprise Competitiveness", *Technology in Society*, Vol.59, pp.1~11.
(47)Lin, R. J., Tan, K. H. and Geng, Y., 2013, "Market Demand, Green Product Innovation, and Firm Performance: Evidence from Vietnam Motorcycle Industry", *Journal of Cleaner Production*, Vol.40, pp.101~107.

(C)1994-2021 China Academic Journal Electronic Publishing House. All rights reserved. http://www.cnki.net

(48)Mallin, C., Michelon, G. and Raggi, D., 2013, "Monitoring Intensity and Stakeholders' Orientation: How Does Governance Affect Social and Environmental Disclosure?", *Journal of Business Ethics*, Vol.114(1), pp.29~43.

(49)Mayer, D. M., Aquino, K., Greenbaum, R. L. and Kuenzi, M., 2012, "Who Displays Ethical Leadership, and Why Does It Matter? An Examination of Antecedents and Consequences of Ethical Leadership", *Academy of Management Journal*, Vol.55(1), pp.151~171.

(50)Meyer, J., and Rowan, B., 1977, "Institutionalized Organizations: Formal Structure as Myth and Ceremony", *American Journal of Sociology*, Vol.83(2), pp.340~363.

(51)Mo, S., Ling, C. D. and Xie, X. Y., 2019, "The Curvilinear Relationship between Ethical Leadership and Team Creativity: The Moderating Role of Team Faultlines", *Journal of Business Ethics*, Vol.154(1), pp.229~242.

(52)Pasricha, P., Singh, B. and Verma, P., 2018, "Ethical Leadership, Organic Organizational Cultures and Corporate Social Responsibility: An Empirical Study in Social Enterprises", *Journal of Business Ethics*, Vol.151(4), pp.941~958.

(53)Pinzone, M., Guerci, M., Lettieri, E., and Huisingh, D, 2019, "Effects of 'Green' Training on Pro-environmental Behaviors and Job Satisfaction: Evidence from the Italian Healthcare Sector", *Journal of Cleaner Production*, Vol.226(2), pp.221~232.

(54)Porter, M. E, 1991, "Towards a Dynamic Theory of Strategy", *Strategic Management Journal*, 1991, Vol.12(2), pp.95~117.

(55)Poussing, N., 2019, "Does Corporate Social Responsibility Encourage Sustainable Innovation Adoption? Empirical Evidence from Luxembourg", *Corporate Social Responsibility & Environmental Management*, Vol.26(3), pp.681~689.

(56)Schaubroeck, J. M., Hannah, S. T., Avolio, B. J., Kozlowski, S. W., Lord, R. G., Treviño, L. K., Dimotakis, N. and Peng, A. C., 2012, "Embedding Ethical Leadership Within and Across Organization Levels", *Academy of Management Journal*, Vol.55(5), pp.1053~1078.

(57)Seman, N. A. A., Govindan, K., Mardani, A., Zakuan, N., Saman, M. Z. M., Hooker, R. E. and Ozkul, S., 2019, "The Mediating Effect of Green Innovation on the Relationship between Green Supply Chain Management and Environmental Performance", *Journal of Cleaner Production*, Vol.229, pp.115~127.

(58)Sheng, S., Zhou, K. Z. and Li, J. J., 2011, "The Effects of Business and Political Ties on Firm Performance: Evidence from China", *Journal of Marketing*, Vol.75(1), pp.1~15.

(59)Shu, C., Zhou, K. Z., Xiao, Y. and Xiao, Y., 2016, "How Green Management Influences Product Innovation in China: The Role of Institutional Benefits", *Journal of Business Ethics*, Vol.133(3), pp.471~485.

(60)Sopjani, L., Stier, J. J., Ritzen, S., Hesselgren, M., and Georen, P., 2019, "Involving Users and User Roles in the Transition to Sustainable Mobility Systems: The Case of Light Electric Vehicle Sharing in Sweden", *Transportation Research Part D-transport and Environment*, Vol.71, pp.207~221.

(61)Suchman, M. C., 1995, "Managing Legitimacy: Strategic and Institutional Approaches", *Academy of Management Review*, Vol.20(3), pp.571~610.

(62)Tolbert, P. S., and Zucker, L. G., 1983, "Institutional Sources of Change in the Formal Structure of Organizations: The Diffusion of Civil Service Reform", *Administrative Science Quarterly*, Vol.28(1), pp.22~39.

(63)Tornikoski, E. T. and Newbert, S. L., 2007, "Exploring the Determinants of Organizational Emergence: A Legitimacy Perspective", *Journal of Business Venturing*, Vol.22(2), pp.311~335.

(64)Tu, Y. and Lu, X., 2013, "How Ethical Leadership Influence Employees' Innovative Work Behavior: A Perspective of Intrinsic Motivation", *Journal of Business Ethics*, Vol.116(2), pp.441~455.

(65)Van den Bergh, J. C. J. M., 2013, "Environmental and Climate Innovation: Limitations, Policies and Prices", *Technological Forecasting and Social Change*, Vol.80(1), pp.11~23.

(66)Wang, D., Feng, T. and Lawton, A., 2017, "Linking Ethical Leadership with Firm Performance: A Multi-dimensional Perspective", *Journal of Business Ethics*, Vol.145(1), pp.95~109.

(67)Wei, Z., Shen, H., Zhou, K. Z. and Li, J. J., 2017, "How Does Environmental Corporate Social Responsibility Matter in a Dysfunctional Institutional Environment? Evidence from China", *Journal of Business Ethics*, Vol.140(2), pp.209~223.

(68)Xie, X., Huo, J., and Zou, H., 2019, "Green Process Innovation, Green Product Innovation, and Corporate Financial Performance: A Content Analysis Method", *Journal of Business Research*, Vol.101, pp.697~706.

(69)Xie, X., Huo, J., Qi, G. and Zhu, K. X., 2016, "Green Process Innovation and Financial Performance in Emerging Economies: Moderating Effects of Absorptive Capacity and Green Subsidies", *IEEE Transactions on Engineering Management*, Vol.63(1), pp.101~112.

(70)Xing, Y., and Starik, M., 2017, "Taoist Leadership and Employee Green Behaviour: A Cultural and Philosophical Microfoundation of Sustainability", *Journal of Organizational Behavior*, Vol.38(9), pp.1302~1319.

(71)Yu, W., Ramanathan, R. and Nath, P., 2017, "Environmental Pressures and Performance: An Analysis of the Roles of Environmental Innovation Strategy and Marketing Capability", *Technological Forecasting and Social Change*, Vol.117, pp.160~169.

(72)Zang, J. and Li, Y., 2017, "Technology Capabilities, Marketing Capabilities and Innovation Ambidexterity", *Technology Analysis & Strategic Management*, Vol.29(1), pp.23~37.

(73)Zhang, H., Young, M. N., Tan, J. and Sun, W., 2018, "How Chinese Companies Deal with a Legitimacy Imbalance when Acquiring Firms from Developed Economies", *Journal of World Business*, Vol.53(5), pp.752~767.

(74)Zhu, Q., Sarkis, J. and Lai, K., 2012, "Green Supply Chain Management Innovation Diffusion and Its Relationship to Organizational Improvement: An Ecological Modernization Perspective", *Journal of Engineering and Technology*, Vol.29(1), pp.168~185.

(75)Zimmerman, M. A. and Zeitz, G. J., 2002, "Beyond Survival: Achieving New Venture Growth by Building Legitimacy", *Academy of Management Review*, Vol.27(3), pp.414~431.

(C)1994-2021 China Academic Journal Electronic Publishing House. All rights reserved. http://www.cnki.net

How Can Green Innovation Solve the Dilemmas of "Harmonious Coexistence"?

Xie Xuemei[a] *and Zhu Qiwei*[b]
(a. School of Economics and Management, Tongji University, Shanghai;
b. School of Management, Shanghai University, Shanghai)

Summary: With the increasing intensification of resource constraints and environmental pollution, green innovation has become a focal topic of corporate sustainability. However, implementing green innovation in emerging markets may be more challenging because of the dominant role of government and the imperfect market institutions. Accordingly, examining how Chinese firms can effectively adopt green innovation to achieve sustainable development is extremely significant. Based on a sample of Chinese heavy pollution manufacturing firms, we collect the data of Corporate Responsibility Reports of 227 listed companies (2013~2018) using the content analysis method and the secondary data from the Wind, CSMAR, and Hexun database. The research ideas are as follows: First, we explore the different effects of various green innovation on sustainability performance. Second, rooted in Chinese traditional culture of "Harmonious Coexistence", we draw on the insights of legitimacy theory to investigate the internal mechanisms of conforming legitimacy and strategic legitimacy linking green innovation to sustainability performance. Third, we further explore the contextual mechanism of ethical leadership by constructing a moderated mediation model. Finally, in the robustness tests, we take 2SLS, variable substitution, Logistic, and Monte Carlo methods to make empirical analysis robust.

We first find that green innovation can improve firms' sustainability performance. But green process innovation can improve corporate environmental performance more significantly than green product innovation, and green product innovation can promote corporate financial performance more significantly than green process innovation. Second, we find that dual legitimacy mediates the relationship between green innovation and sustainability performance. Third, we find that ethical leadership positively moderates the indirect effect of green process innovation on sustainability performance via dual legitimacy; however, it fails to significantly moderate the indirect effect of green product innovation on sustainability performance via dual legitimacy. Hence, this study designs a post-hoc test and finds that the interaction between ethical leadership and green subsidies can strengthen the indirect effect of green product innovation on sustainability performance via dual legitimacy. According to the above findings, we provide the following policy recommendations. Firstly, given that the ethical leadership is essential to effectively implement environmental policies (e.g., subsidies) for green innovation, the government should conduct the ethical evaluation of leaders during allocating green subsidies. Secondly, given that the environmentally-friendly enterprises usually require a long-term shift from conceptual awareness (i.e., light green innovation) to behavioral inertia (i.e., dark green innovation), the government should formulate multi-stage and multi-type green policies to encourage firms to actively conduct green innovation practices.

This research makes the following contributions: First, we enrich the theoretical research of the relationship between green innovation and corporate performance by comparing the effects of different types of green innovation. Second, we identify the internal "black box" between green innovation and sustainability performance from the perspective of legitimacy. Third, we further expand the theoretical boundary of green innovation research by investigating the moderating role of ethical leadership. Future research needs to examine more types and contingency factors of green innovation, or further explore the green innovation behaviors of different members within the supply chain, so as to further advance our research results.

Keywords: green process innovation; green product innovation; conforming legitimacy; strategic legitimacy; ethical leadership

JEL Classification: F270.70

(C)1994-2021 China Academic Journal Electronic Publishing House. All rights reserved. http://www.cnki.net
