# Supplementary material for: How does equity restriction affect innovation quality? Evidence from listed manufacturing companies in China
Source: PLoS One. 2023 Dec 7;18(12):e0295553. doi: 10.1371/journal.pone.0295553 (PMC10703261; doi:10.1371/journal.pone.0295553)
Supplement: S1 Dataset — (ZIP) [file pone.0295553.s001.zip › Supporting information/S1 Dataset/╩╨│í╗»╓╕╩2/1997-2022─Ω╩╨│í╗»╓╕╩2║1⁄4╖╓╧ε╓╕╩2ú¿╫ε╓╒╩2╛▌▓╬┐╝╬─╧╫│⌡╩╝╩2╛▌╧Ω╧╕┤a└φ┤·┬δú⌐/1997-2022─Ω╩╨│í╗»╓╕╩2║1⁄4╖╓╧ε╓╕╩2ú¿╫ε╓╒╩2╛▌+▓╬┐╝╬─╧╫+│⌡╩╝╩2╛▌+╧Ω╧╕┤a└φ┤·┬δú⌐/╓╒╝1⁄2┐╪╣╔╣╔╢1⁄2┐╪╓╞╚¿╙δ╫╘╙╔╧╓╜≡┴≈╣2╢╚═╢╫╩_╙ß║∞║ú.pdf]

终极控股股东控制权与自由现金流过度投资

俞红海　徐龙炳　陈百助

内容提要:本文从投资行为视角,研究控股股东侵占与公司治理问题。理论上首次通过动态模型方法,研究发现股权集中、控股股东的存在会导致公司过度投资,控股股东控制权与现金流权分离进一步加剧了这一行为,同时自由现金流水平也对过度投资有正向影响;现金流权水平的提高、公司治理机制的改善,则可以有效抑制过度投资。实证上基于[illegible](2006)预期投资模型,采用面板数据方法,研究表明控制权与现金流权分离度对过度投资有显著为正的影响,相对于私人控股,政府控股公司过度投资更严重,而外部治理环境的改善一定程度上抑制了过度投资。这一研究为中国改革开放以来大规模低效率投资现象提供了一定的解释,同时也为控股股东侵占提供了新的证据。

关键词:终极控制权　自由现金流　过度投资　公司治理　面板数据

一、引　言

自[illegible](1999)以来,一系列的研究([illegible], 2000;[illegible], 2002)表明,除英、美之外,股权集中现象在世界范围内普遍存在。在这一背景下,公司治理研究的重点,开始从管理层与投资者之间的代理问题,转移到控制性大股东与外部中小投资者之间的代理问题。现有文献分别从关联交易([illegible], 2002;[illegible], 2002;[illegible], 2006)、股利政策([illegible], 2000;[illegible], 2001)、债务融资([illegible], 2003;[illegible], 2008)等角度研究了控股股东侵占行为。与此同时,尽管有部分学者([illegible], 2006;杨华军等, 2007)从公司治理角度探讨了公司投资行为,但并未针对股权集中市场,从终极控制权、尤其是控制权与现金流权分离角度进行研究。股权集中、控股股东的存在会对公司投资决策产生怎样的影响?控股股东是否会利用公司投资行为进行侵占?股权集中及金字塔式结构能否解释中国改革开放以来大规模低效率投资现象([illegible], 2005)?本文将就这些问题展开系统研究。

理论上本文基于[illegible](2002)、[illegible](2002)等的工作,通过动态模型研究股权集中下的公司投资决策。实证上本文基于[illegible](2006)预期投资模型,利用2004—2007年中国上市公司样本数据,采用面板数据方法对理论分析进行检验。相对于已有文献,本文的主要贡献在于:第一,在理论上首次通过动态模型对过度投资进行研究,揭示了股权集中市场上,控股股东利用控制权、尤其是通过金字塔结构影响公司投资决策进行侵占的内在机制,也为杨华军等(2007)、胡建平等(2007)的实证发现提供了理论解释;第二,在实证上首次发现控股股东控制权与

俞红海、徐龙炳,上海财经大学金融学院,邮政编码:200433,电子信箱:[illegible]@163.com,[illegible];陈百助,美国南加州大学马歇尔商学院,电子信箱:[illegible]。本文得到国家自然科学基金项目(70673056, 70803027, 70873080)、教育部新世纪优秀人才支持计划项目(NCET-07 0533)、上海市哲学社会科学规划项目(2008[illegible]003)、上海市重点学科建设项目([illegible]802)、上海财经大学"211工程"三期重点学科建设项目资助。作者感谢匿名审稿人,美国南加州大学[illegible]和[illegible]教授,波士顿学院钱军教授,上海财经大学金融学院李曜、刘莉亚、韩其恒教授,以及"2009年中国金融国际年会"上许年行博士等提供的宝贵修改意见。当然文责自负。

(C)1994-2021 China Academic Journal Electronic Publishing House. All rights reserved. http://w

现金流权分离加剧了过度投资,为控股股东侵占提供了新证据,也进一步支持了[illegible](1986)、[illegible](1990)的自由现金流代理理论。同时,本文的研究也为中国改革开放30多年来大规模低效率投资现象提供了一定的解释。此外,相对于[illegible](2006)、杨华军等(2007)的研究,本文在实证研究过程中采用面板数据方法,克服了混合数据方法存在的不足,使得研究结论更加可靠。

二、文献回顾

本文的研究涉及股权集中下控股股东侵占及公司投资行为两方面,本部分分别就这两方面的文献进行系统述评。

(一)股权集中与控股股东侵占

自[illegible](1999)以来,一系列的研究([illegible],1999;[illegible],2000;[illegible],2002)表明,在世界范围内,除英、美之外,股权集中是一种更为普遍的所有权结构,且控股股东往往是国家或家族。相应地,公司治理研究的重点,开始从管理层与投资者之间的代理问题,转移到控制性大股东与外部中小投资者之间的代理问题([illegible],1997)。大量研究([illegible],2002;[illegible],2002;[illegible],2003)表明,控股股东持股具有侵占和激励效应,一方面控股股东控制权水平越高,公司价值越低,另一方面控股股东现金流权水平越高,公司价值越大。[illegible](2000)将控股股东利用控制权进行侵占的行为称为"掏空([illegible])",并指出了具体的掏空方式。理论上,[illegible](2000)、[illegible](2002)用静态模型方法,[illegible](2002)、[illegible](2006)用动态模型方法,分别对控股股东利用控制权侵占问题进行了研究,在一定程度上揭示了股权集中下控股股东侵占与公司治理的内在机制。

在控股股东具体侵占方式上,[illegible](2002),[illegible](2002)和[illegible](2006)利用关联交易数据,分别对印度、韩国和香港市场上的控股股东侵占行为进行直接研究,进一步佐证了上述理论研究结论。[illegible](2000)、[illegible](2001)从股利政策角度研究了控股股东侵占,发现法制环境越差,股利发放越少。[illegible](2003)、[illegible](2008)则从债务融资角度研究了控股股东侵占,发现在股权集中市场上,债务融资并非如[illegible](1986)所言,可以抑制公司内部代理问题,反而便于控股股东侵占。

在国内市场上,部分学者基于[illegible](1999)及[illegible](2002),研究了股权集中对公司绩效或价值的影响(刘芍佳等,2003;徐莉萍等,2006;王鹏和周黎安,2006;王鹏,2008),发现控股股东持股对公司价值具有侵占效应和激励效应,其中王鹏(2008)进一步表明控股股东控制权侵占效应随着投资者法律保护的增强而减弱。与此同时,在具体侵占方式上,李增泉等(2005)、高雷等(2006)从控股股东资金占用角度,肖珉(2005)、雷光勇等(2007)、王化成等(2007)从股利政策角度,分别探讨了控股股东侵占问题。

上述针对股权集中市场下控股股东侵占研究,主要集中在控制权与公司价值,以及关联交易、股利政策及债务融资政策等,据笔者所知,目前尚未有文献从公司投资行为角度系统探讨控股股东侵占问题。而投资决策作为公司最重要经营决策之一,影响甚至决定着公司价值。因此分析控股股东对投资决策的影响,对揭示控股股东侵占与公司价值内在关系显得尤为重要。

(二)投资行为与公司治理

自[illegible](1988)以来,国内外学者主要围绕投资现金流的敏感性对公司投资行为进行了广泛研究。其中[illegible](1988),[illegible](1999)从信息不对称角度研究了投资现金流敏感性,认为是公司内外部的信息不对称性导致了这一问题;而[illegible](1998),[illegible](2005)等认为是管

(C)1994-2021 China Academic Journal Electronic Publishing House. All rights reserved. http://w

理层与投资者之间的代理问题造成了投资现金流的敏感性。[illegible](2006) 采用投资预期模型,在控制成长机会和融资约束即信息不对称的前提下,采用大样本数据,研究表明自由现金流过度投资问题来自管理层与投资者之间的代理问题。

国内市场上,杨华军等 (2007) 基于 [illegible](2002),结合中国的制度环境研究了自由现金流的过度投资问题,发现地方政府控股和地方政府干预显著提高了自由现金流的过度投资,而金融发展可以降低这一现象。胡建平等 (2007) 基于 [illegible](2006) 方法,在控制成长机会和融资约束的基础上,发现自由现金流过度投资问题,支持了代理理论。何源等 (2007) 通过建立负债融资对大股东过度投资行为的相机治理模型,研究表明控股股东持股比例越高,因谋取私利而导致的过度投资趋势越弱,同时负债融资能够抑制控股股东过度投资行为。

上述针对公司投资行为的研究中,[illegible](2006) 研究了股权分散的美国市场上,公司治理对过度投资的影响。胡建平等 (2007) 研究了股权集中的中国市场上,自由现金流对过度投资的影响,但并未考虑终极控股股东因素的影响;杨华军等 (2007) 虽然考虑了终极控股股东性质,但未考虑控股股东控制权水平和现金流权水平等因素,并且该文的实证分析中,在计算过度投资和自由现金流时,仅考虑了预期投资,而忽略了维持性投资,因此其结论有一定的偏差。

三、理论模型与研究假设

本部分基于 [illegible] (2002)、[illegible] (2002) 等理论研究,结合内外部公司治理机制,建立两期动态决策模型,分析不同性质终极控股股东持股与自由现金流过度投资关系。和上述研究不同的是,我们进一步考虑了控股股东性质对控制权私利的影响,以及控制权与现金流权分离对侵占成本的影响,并且首次专门对公司投资决策进行理论研究。基本思路是:第一期出现一个投资机会,控股股东对是否投资进行决策,第二期投资产生回报,控股股东在控制权私利与股利分红之间进行权衡,实现利益最大化。具体采用逆向求解法进行求解。

(一) 基本假设

(1) 公司在满足维持性投资及 [illegible]>0 项目投资之后,还有自由现金流 [illegible];

(2) 市场上出现一个新的投资机会,该投资机会的回报分两种情况,一种是高回报,发生概率为 [illegible],相应投资回报为 [illegible],另一种为低回报,发生概率为 (1 − [illegible]),相应投资回报为 [illegible],其中 [illegible] > [illegible] > [illegible];

(3) 终极控股股东现金流权水平为 α,相应控制权水平为 [illegible],其中 [illegible] 为控制权与现金流权分离度,是控制权与现金流权之比,[illegible] ≥ 1;

(4) 控股股东侵占比例为 [illegible],则剩余用于股利分配的部分为 (1 − [illegible]) [illegible],其中 [illegible] = [illegible] 或 [illegible];

(5) 侵占成本函数为 [illegible] = [illegible]([illegible]),其中 [illegible] 表示侵占比例,[illegible] 表示内外部公司治理机制,[illegible] 越大代表治理机制越好,[illegible] 则表示控制权水平。侵占成本函数与各变量之间有如下关系:[illegible] > 0,[illegible] > 0,即侵占成本随着侵占比例的增加,以递增方式增加;[illegible] > 0,即公司治理机制的改善,将增加侵占成本;[illegible] < 0,即随着控制权水平提高,相应的侵占成本降低。

(6) 不同控股股东性质对控制权私利有影响,控制权私利为 [illegible],其中 [illegible] 是控制权私利放大系数,代表控股股东不同性质。

(二) 第 2 期:控股股东最优侵占决策

第 2 期项目投资产生回报,控股股东选择最优侵占比例,来实现其目标利益最大化,其中目标利益包括股利分红所得及侵占净收益两部分,用 [illegible] 表示控股股东目标利益,控股股东在第二期面临的决策如方程 (1):

(C)1994-2021 China Academic Journal Electronic Publishing House. All rights reserved. http://w

$$牗觼甉犴淳 = 牗觼甉[\alpha(1 - 昊)犁 + 犽昊犁 - 犆(昊,犽,狏犮)犁] \quad (1)$$

和犑狅犺狀狊狅狀等(2000犪)及犑狅犺狀狊狅狀等(2002)不同的是,我们考虑了控股股东性质 犳 对控制权私利的影响,即同样的侵占比例,由于不同性质控股股东使用方式不同,导致其产生的收益是有差异的。与此同时,在犑狅犺狀狊狅狀等(2000犪)、犑狅犺狀狊狅狀等(2002)中,侵占成本由侵占比例和公司治理水平对投资者的保护 犽决定,且和侵占比例呈二次关系,如方程 $犆(昊,犽) = \frac{犽昊^2}{2}$,这一侵占成本函数仅考虑了侵占比例和法制环境的影响,忽略了控制权水平的影响,而事实上随着控制权水平的增加,控股股东的侵占成本将下降,例如控股股东拥有 20%的控制权和 60%的控制权对应的侵占成本是不同的。此外,本文中 犽表示公司内外部治理机制,不仅包括外部法制环境,还包括内部独立董事比例、外部机构投资者持股等。为简化起见,本文设定侵占成本函数如方程(2):

$$犆(昊,犽,狏犮) = \frac{犽昊^2}{2狏犮} \quad (2)$$

相应的控股股东在第二期的最优侵占比例为

$$昊 = \frac{狏犮(犳 - \alpha)}{犽} \quad (3)$$

方程(3)表示终极控股股东最优侵占比例,其中 狏犮 表示控制权水平。从方程(3)我们可以看到,给定控制权水平,现金流权水平与最优侵占比例负相关;给定现金流权水平,控制权水平与最优侵占比例正相关;给定控制权或现金流权,两者分离度越大,侵占比例越高。此外,公司治理水平越高,则控股股东侵占比例越低。

(三)第 1 期:投资决策

作为上市公司,以全体股东利益最大化为目标,若未来平均投资回报高于投资额度,则公司进行投资,即

$$犘犚_{犺} + (1 - 犘)犚_{犾} > 犐 \quad (4)$$

从方程(4)中,我们可以得到以全体股东利益为出发点进行投资时,所要求的高回报的临界概率,如方程(5):

$$犘_1 = \frac{犐 - 犚_{犾}}{犚_{犺} - 犚_{犾}} \quad (5)$$

$犘_1$ 表示当上市公司管理层代表全体投资者利益进行投资时所要求的高回报概率临界值,即只有当外部投资项目产生高回报的概率 $犘 > 犘_1$ 时,公司将进行投资。

而作为控股股东,在投资过程中,除了获得相应份额的股利分红之外,还有控制权私利,其对应的投资决策为

$$犘[\alpha(1 - 昊) + 犽昊 - 犆(昊,犽,狏犮)]犚_{犺} + (1 - 犘)[\alpha(1 - 昊) + 犽昊 - 犆(昊,犽,狏犮)]犚_{犾} \geq \alpha犐 \quad (6)$$

则相应所要求的高回报的临界概率为

$$犘_2 = \frac{犐 - 犚_{犾}}{犚_{犺} - 犚_{犾}} - \frac{(犳 - \alpha)昊 - 犆}{[\alpha(1 - 昊) + 犽昊 - 犆](犚_{犺} - 犚_{犾})} \quad (7)$$

即当外部投资项目产生高回报的概率 $犘 > 犘_2$ 时,控股股东作出投资决策。

以控股股东利益为出发点所要求的高回报临界概率,和以上市公司全体投资者利益为出发点所要求的高回报临界概率的差值,即代表了过度投资出现的可能性,如方程(8):

$$\Delta犘 = 犘_1 - 犘_2 = \frac{(犳 - \alpha)昊 - 犆}{[\alpha(1 - 昊) + 犽昊 - 犆](犚_{犺} - 犚_{犾})} \quad (8)$$

(C)1994-2021 China Academic Journal Electronic Publishing House. All rights reserved. http://www.

Δ摩表示从公司角度出发不应该进行投资的项目而从控股股东角度出发应该进行投资的项目,由于控股股东在上市公司决策中的地位,导致了上市公司过度投资。

为了分析内在关系,我们基于终极控股股东第2期最优侵占决策,选择具体函数形式(2)和(3),则

$$卷 = Δ摩料 = (摩_1 - 摩_2)料 = \left[1 - \frac{2犸}{2犸 + 犾(犷 - α)^2}\right]\frac{料^2}{犚_{抗} - 犚_{扶}} > 0 \qquad (9)$$

Δ摩料即为过度投资部分,由此得到命题1:

命题1:股权集中、控股股东的存在,导致公司出现过度投资现象。

命题1表示控股股东代理成本导致了自由现金流过度投资。这一思想和犑犲狀狊犲狀(1986)、犚犻犮犺犪狉犱狊狅狀(2006)关于过度投资的内涵及产生根源的分析相一致,即控制权私利的存在导致了公司过度投资现象。①

进一步从方程(9)我们可以看到,公司自由现金流水平越高、控股股东控制权与现金流权分离度越大,公司过度投资现象越严重;公司治理水平越高,公司过度投资现象越轻。此外,考虑控制权私利及现金流权水平对过度投资的影响,在 犷 - α > 0 的条件② 下,控制权私利越大,过度投资越严重;现金流权水平越高,过度投资现象越轻。

基于上述分析,提出本文命题2:

命题2:公司自由现金流水平越高、控股股东控制权与现金流权分离度越大、控制权私利越大,公司过度投资现象越严重;控股股东现金流权水平越高、公司治理水平越高,过度投资现象越轻。

(四)研究假设

在中国经济转轨过程中,国有企业承担了大量政策性负担(犔犻狀犲狋犪犾,1998;林毅夫等,2004),包括促进经济增长、提高就业率以及增加税收等。政府作为国有企业控股股东,其控制权私利体现在政策性目标的实现,而这些政策性目标的实现,具有较大的溢出效应,往往会带动其他企业发展、推动经济增长,例如大规模基础设施建设对经济发展的推动。因此,相对于侵占所获取的货币性收益,这些政策性目标的实现具有更大的效应,能够产生更大的价值,即相对于私人控股,政府控股下的控制权私利更大。结合命题2,提出本文需要检验的假设:

犎1:自由现金流水平越高,公司过度投资现象越严重;

犎2:控股股东控制权与现金流权分离度越大,公司过度投资现象越严重;

犎3:相对于私人控股而言,政府控股的上市公司过度投资现象更严重;

犎4:控股股东现金流权水平越高,公司过度投资现象越轻;

犎5:公司治理机制越完善,则公司过度投资现象越轻,其中治理机制包括外部治理环境、独立董事比例,以及外部机构投资者持股等。

四、研究设计

(一)样本选择与数据来源

本文数据主要包括两部分,第一部分是2004—2007年终极控股股东数据,包括终极控股股东性质、现金流权和控制权水平,该数据是通过从上海证券交易所和巨潮网站收集上市公司年报,再从公司年报"实际控制人"一栏中的控制权链图进行手工整理而得,其中控股股东的定义是按照犔犪犘狅狉狋犪

---

① 所不同的是,犑犲狀狊犲狀(1986)、犛狋狌犾狕(1990)以及犚犻犮犺犪狉犱狊狅狀(2006)研究的是股权分散的美国市场,是由于管理层控制权私利的存在导致了过度投资,而中国市场则是由于控股股东控制权私利的存在导致了过度投资。

② 一般来说独占获得的全部收益高于分红所得部分收益,因此 犷 - α > 0 满足。

(C)1994-2021 China Academic Journal Electronic Publishing House. All rights reserved. http://www

[illegible](2002),指拥有上市公司超过10%投票权的终极控制人,当存在两个或两个以上超过10%投票权的大股东时,取投票权最大者为公司终极控股股东。现金流权和控制权水平的定义和计算基于[illegible](1999),控股股东的性质分为政府、私人及其他,其中政府包括中央政府和地方政府,私人包括终极控制人为个人、夫妻、家族、村委会、职工委员会等形式。本文剔除控股股东性质为其他,以及终极控制权发生变动的上市公司。第二部分是2004—2007年相关财务数据①,包括投资、经营性现金流量、财务杠杆、总资产回报率等,均来自[illegible]数据库,剔除金融行业和[illegible]股票;经过数据匹配之后,总共获得2004—2007年1086家上市公司2918条记录。

此外,外部治理环境的度量,本文采用樊纲等(2007)中相关指数,分别选取"减少政府干预"指标并取相反数,"市场中介组织发育和法律制度环境"指标,以及各地区市场化指数来作为外部治理环境的度量指标,这三个指标的值越大,代表外部治理环境越好。由于该报告仅提供2001—2005年数据,本文直接选取其中2004—2005年数据,同时考虑到治理环境的稳定性和延续性,采用2001—2005年各地区指标的年平均增长幅度作为2005—2006年,以及2006—2007年增长幅度,来获取2006—2007年各地区外部治理环境数据。

(二)研究模型与变量定义

本文实证上基于[illegible](2006)预期投资模型,在考虑投资机会和融资约束的基础上,用上一年数据估计本年度投资,如方程(10),其中投资分为三部分,包括维持性投资(用固定资产折旧和无形资产摊销来衡量)、预期投资和过度投资,该方程的残差表示真实投资和预期投资的差值,若残差为正,表示过度投资;若为负,表示投资不足。本文中仅考虑投资为正,即过度投资问题。需要指出的是,本文用过去两年平均销售增长率来代表投资机会,而不是和市场价格有关的指标如市盈率、市净率等,其中的原因在于:一方面中国股票市场远未达到有效,股票价格受投资者行为和宏观政策影响严重,另一方面本文研究样本区间恰好涉及股权分置改革,股改前后公司股本往往会发生变化,因此选择了销售增长率这一不易受股票市场影响的指标来代表投资机会。这一度量方法被国内外学者广泛采用([illegible],2002;沈艺峰等,2008)。方程(10)中[illegible]代表公司个体效应,若[illegible]和其他解释变量相关,则采用固定效应([illegible])模型;若[illegible]和其他解释变量无关,则采用随机效应([illegible])模型。各变量的定义参见表1。

$$\begin{aligned}\text{[illegible]}_{\text{[illegible]}} = {} & \alpha_0 + \alpha_1 \text{[illegible]}_{\text{[illegible]}-1} + \alpha_2 \text{[illegible]}_{\text{[illegible]}-1} + \alpha_3 \text{[illegible]}_{\text{[illegible]}-1} + \alpha_4 \text{[illegible]}_{\text{[illegible]}-1} \\ & + \alpha_5 \text{[illegible]}_{\text{[illegible]}-1} + \alpha_6 \text{[illegible]}_{\text{[illegible]}-1} + \alpha_7 \text{[illegible]}_{\text{[illegible]}-1} + \alpha_8 \text{[illegible]}_{\text{[illegible]}-1} \\ & + \alpha_9 \text{[illegible]} + \sum \text{[illegible]} + \sum \text{[illegible]} + \text{[illegible]}_{\text{[illegible]}} + \varepsilon_{\text{[illegible]}} \end{aligned} \quad (10)$$

在通过投资预期模型获得预期投资,过度投资及自由现金流的基础上,进一步研究自由现金流及控股股东持股和公司治理机制对过度投资的影响,解释变量包括控制权与现金流权分离度,自由现金流、控股股东性质、现金流权水平,以及内外部公司治理机制等,此外为了更深入地研究控制权与现金流权分离度对过度投资的影响,还包括了分离度的平方项,具体如方程(11)②,相关变量定义见表1。

$$\begin{aligned}\text{[illegible]}_{\text{[illegible]}} = {} & \beta_0 + \beta_1 \text{[illegible]}_{\text{[illegible]}} + \beta_2 \text{[illegible]}_{\text{[illegible]}} + \beta_3 \text{[illegible]}^2_{\text{[illegible]}} + \beta_4 \text{[illegible]}_{\text{[illegible]}} + \beta_5 \text{[illegible]}_{\text{[illegible]}} \\ & + \beta_6 \text{[illegible]}_{\text{[illegible]}} + \beta_7 \text{[illegible]}_{\text{[illegible]}} + \beta_8 \text{[illegible]}_{\text{[illegible]}} + \text{[illegible]}_{\text{[illegible]}} + \varepsilon_{\text{[illegible]}} \end{aligned} \quad (11)$$

---

① 文中投资机会用前两年销售增长率的算术平均值来表示,因此销售增长率数据选择从2002年到2006年。

② 本文实证分析过程中也考虑了自由现金流与控制权及现金流权分离度,自由现金流与政府控股哑变量的交叉项影响,在分析中发现交叉项均不显著,并且导致了较为严重的多重共线性,因此在结果报告中并未包含交叉项。

(C)1994-2021 China Academic Journal Electronic Publishing House. All rights reserved. http://w

表 1　　变量名称、符号及定义

变量名称	符号	变量描述
总投资	[illegible]	取现金流量表中投资活动净现金流量的相反数,并除以本年度总资产。
维持性投资	[illegible]	取现金流量表附注中固定资产折旧和无形资产摊销之和,并除以本年度总资产。
新投资	[illegible]	[illegible] = [illegible] − [illegible]
预期新投资	[illegible]	投资预期模型(10)的预测值。
过度投资	[illegible]	投资预期模型(10)中正的残差值。
经营现金流量	[illegible]	取现金流量表中的经营性活动现金净流量,除以年末总资产。
自由现金流量	[illegible]	[illegible] = [illegible] − [illegible] − [illegible]
现金流权	[illegible]	[illegible] = $\sum \prod$ [illegible],[illegible]…[illegible] 为第[illegible]条控制链的链间控股比例。
控制权	[illegible]	终极控股股东直接和间接持有的投票权总和,具体参见 [illegible](1999)。
控制权与现金流权分离度	[illegible]	控制权与现金流权的比值。
金字塔层数	[illegible]	从终极控股股东到上市公司的控制链层数。
独立董事比例	[illegible]	独立董事占董事会的比例。
外部机构持股	[illegible]	上市公司十大流通股股东持股占总股本的比例。
政府干预指数	[illegible]	取自樊纲等(2007)报告"减少政府干预"指数,并取相反数。
法制水平	[illegible]	取自樊纲等(2007)报告"市场中介组织发育和法律制度环境"指数。
市场指数	[illegible]	取自樊纲等(2007)报告市场化指数。
投资机会	[illegible]	取前两年销售增长率的算术平均值。
会计收益	[illegible]	总资产收益率。
股票回报	[illegible]	股票年回报率。
资产负债率	[illegible]	年末总负债/年末总资产。
现金持有水平	[illegible]	资产负债表中的货币资金除以年末总资产。
公司上市时间	[illegible]	以公司公告上市年度到相应年度的差值。
公司规模	[illegible]	年末总资产取自然对数。
股权分置改革	[illegible]	若已发生股权分置改革,设为 1,否则为 0。
年度控制变量	[illegible]	控制年度固定效应,2004—2007 共四年,设置三个控制性哑变量。
行业控制变量	[illegible]	行业控制变量,制造业按二级分类,其他行业按一级分类,剔除制造业后共 21 个行业,并以综合业为基础,共设 20 个控制哑变量。

五、实证结果与分析

为了对本文的理论分析进行检验,本部分基于 [illegible](2006) 预期投资理论模型,在计算预期投资、过度投资和自由现金流的基础上,研究公司治理与自由现金流过度投资关系问题,其中核心是研究控制权与现金流权分离对过度投资的影响。

(C)1994-2021 China Academic Journal Electronic Publishing House. All rights reserved. http://w

(一) 描述性统计分析

表 2 提供了主要变量的描述性统计结果。从表 2 可以看到,平均来说,终极控股股东拥有 35 37%的上市公司现金流权水平,以及 41 51%的控制权水平,平均的控制权与现金流权分离度为 1 48,说明中国市场两权分离现象较为严重;同时分离度最小为 1,最大则达到 27 04,说明不同公司间分离度具有较大的差异性。与此同时,金字塔结构在中国市场普遍存在,平均来说每个控股股东通过 2 42 层控制链来控制上市公司,最高则达到 8 层。此外,公司规模、总资产收益率、股票年回报率等指标在不同公司间均具有较大的变异性。

表 2 变量描述性统计

	均值	中位数	最大值	最小值	方差
[illegible]	35 37	33 05	88 06	0 53	17 65
[illegible]	41 51	40 60	88 06	8 94	15 82
[illegible]	1 48	1	27 04	1	1 41
[illegible]	2 42	2	8	1	0 83
[illegible]	21 56	21 45	27 30	18 50	1 05
[illegible]	8 06	－8 65	533 33	－90 93	56 15
[illegible]	5 62	5 44	138 29	－84 20	7 38
[illegible]	6 35	6	14	1	3 20
[illegible]	50 58	51 34	368 25	4 30	18 77

(二) 预期投资与过度投资

基于方程 (10) 对公司预期投资进行分析,经 [illegible] 检验,拒绝原假设,因此采用固定效应模型进行估计,同时考虑到存在的异方差问题,本文对实证结果进行 [illegible] (1980) 调整,得到的结论如表 3。从表 3 可以看到,考虑融资约束及其他影响因素,投资机会的影响系数为正但不显著,其中的原因在于,用过去两年销售平均增长率无法反映公司面临的全部投资机会。股票年增长率也在一定程度上体现了未来投资机会,对公司投资有显著为正的影响。同时,公司总资产回报率对公司投资有显著的正向影响,而财务杠杆对企业投资具有较大的约束作用,且在 1% 水平下显著。公司规模及上一期投资对本期投资活动的影响显著为负,这与 [illegible] (2006) 相反,其中原因很有可能是因为不同制度背景及不同市场发展阶段造成的。

总体而言,模型 1 的 [illegible] 达 0 109,具有较强解释力。下面进一步用模型 1 生成的预期投资及过度投资进行分析。

(三) 公司治理与自由现金流的过度投资

在获得自由现金流和过度投资的基础上,进一步基于方程 (11) ,分析公司治理因素对自由现金流过度投资的影响。经 [illegible] 检验,无法拒绝原假设,因此采用随机效应进行估计,结果如表 4。在表 4 中我们分别用法制水平、政府干预指数以及市场化指数作为外部治理环境的代理变量。从表 4 可以看到,自由现金流对过度投资影响为正,尽管不显著,一定程度上验证了本文的假设 [illegible]1,即自由现金流水平越高,过度投资越严重。与此同时,从模型 2、3、4 可以看到,无论是用法制水平、政府干预指数还是各地区市场化指数来度量外部治理环境,控制权与现金流权分离度对过度投资影响始终为正且在 5% 或 10% 水平下显著,从而很好地验证了本文的假设 [illegible]2,其原因是随着控制权与现金流权分离度的增加,控股股东侵占动机越强烈,导致的上市公司过度投资越严重。同时也可以看到,随着分离度本身的增加,其对过度投资的边际影响逐步减小,即控制权与现金流权分离度对过度投资的影响呈倒 [illegible] 型,当控制权与现金流权分离度较小时,分离度的增加所导致的上市公司过度投资更加严重。

(C)1994-2021 China Academic Journal Electronic Publishing House. All rights reserved. http://w

表3 投资预期模型估计结果

变量	模型1
[illegible]	0 0001<br>(0 79)
[illegible]	－0 0193<br>(－1 93)
[illegible]	0 0008<br>(2 16)
[illegible]	0 0001<br>(3 65)
[illegible]	－0 0012<br>(－4 40)
[illegible]	－0 0012<br>(－1 73)
[illegible]	0 0988<br>(3 41)
[illegible]$_{-1}$	－0 0547<br>(－1 74)
[illegible]	－0 0004<br>(－0 07)
[illegible]检验值	160 65
[illegible]值	0 00
[illegible]	0 109

注： 、 和 分别表示1%、5%和10%的显著性水平，括号内为[illegible]值，经过[illegible]异方差调整。

表4 公司治理与自由现金流过度投资

变量	预期符号	模型2	模型3	模型4
[illegible]	＋	0 0651<br>(1 04)	0 0642<br>(1 03)	0 0647<br>(1 03)
[illegible]	－	0 0002<br>(1 37)	0 0002<br>(1 27)	0 0002<br>(1 29)
[illegible]	＋	0 0099<br>(1 94)	0 0094<br>(1 82)	0 0095<br>(1 85)
[illegible]	?	－0 0005<br>(－1 73)	－0 0005<br>(－1 63)	－0 0005<br>(－1 68)
[illegible]	＋	0 0129<br>(1 72)	0 0126<br>(1 68)	0 0127<br>(1 69)
[illegible]	－	－0 0178<br>(－0 45)	－0 0193<br>(－0 49)	－0 0183<br>(－0 47)
[illegible]	－	0 0007<br>(2 51)	0 0007<br>(2 51)	0 0007<br>(2 53)
[illegible]	－	－0 0012<br>(－1 50)		
[illegible]	－		－0 0008<br>(－0 91)	
[illegible]	－			－0 0015<br>(－1 05)
[illegible]检验值		0 14	0 43	0 79
[illegible]值		1 00	0 99	0 99
[illegible]		0 055	0 050	0 052

注： 、 和 分别表示1%、5%和10%的显著性水平，括号内为[illegible]值，经过[illegible]异方差调整。

与此同时，从表4可以看到，相对于私人控股，政府控股对过度投资有显著为正的影响，从而验证了本文的假设[illegible]3，说明政府作为控股股东时，为了实现其控制权私利，更有可能使上市公司进行过度投资，这与我们经常看到的国有企业大规模低效率投资现象相符。此外，现金流权水平的影响和预期相反但显著水平很低，即控股股东现金流权水平对上市公司过度投资并未起到有效的抑制作用，无法验证本文的假设[illegible]4。而对公司治理机制的分析发现，外部治理环境及独立董事比例对过度投资的影响如预期，尽管不显著但在一定程度上抑制了过度投资，从而部分验证本文的假设[illegible]5，然而外部机构持股却和预期相反，非但没有抑制过度投资反而加剧了过度投资，体现出外部机构投资者与内部控股股东的共谋行为。

六、稳健性分析

在上述分析中，类似于[illegible](2002)，我们采用平均销售增长率来度量投资机会。作为影响预期投资及过度投资的重要变量，投资机会的度量会对结论的可靠性产生一定的影响。为此，我们进一步借鉴[illegible](2006)，使用[illegible](价格与盈余比)、[illegible](市场价值与账面价值比)，以及[illegible](2006)构建的新指标[illegible]来度量投资机会，其中[illegible]、[illegible]越大，[illegible]越小，代表投资机会越好。

(C)1994-2021 China Academic Journal Electronic Publishing House. All rights reserved. http://w

从表5可以看到，用[illegible]来度量投资机会时，对投资的影响和预期一致且显著，[illegible]对投资影响为正

但不显著,摩棬对投资的影响很小且和预期相反,其中的原因,很有可能是因为使用和价格相关变量度量投资机会时受市场影响较大。

表6是分别用市净率摩犫、市盈率摩棬和犜犙来度量投资机会进行投资预期的基础上,进一步分析过度投资与公司治理关系。从表6可以看到,控制权与现金流权分离度对过度投资影响显著为正,并且其边际影响随着分离度的增加而减小,相对于私人控股,政府控股下的过度投资更严重。此外,外部法制水平[①]一定程度上抑制了过度投资,尤其是当用犜犙衡量投资机会时,法制水平对过度投资的影响显著为负。因此,总体来说,表6进一步验证了本文的相关假设,并和表4所得的基本结论保持一致。

此外,不同于上述用预期投资模型残差表示过度投资,我们用行业均值作为最优投资水平,超过行业均值部分投资作为过度投资进行稳健性分析,基本结论和表4一致,进一步证明本文结论是可靠的。限于篇幅,本文不再具体报道。

表5　投资预期模型估计结果

	模型5<br>摩犫	模型6<br>摩棬	模型7<br>犜犙
[illegible]	0 0002<br>(1 03)	－0 0000<br>(－0 22)	－0 0167<br>(－2 37)
[illegible]	－0 0194<br>(－1 94)	－0 0189<br>(－1 90)	－0 0155<br>(－1 10)
[illegible]	0 0007<br>(2 09)	0 0008<br>(2 15)	0 0012<br>(3 18)
[illegible]	0 0001<br>(3 52)	0 0001<br>(3 63)	0 0001<br>(2 05)
[illegible]	－0 0012<br>(－4 40)	－0 0012<br>(－4 38)	－0 0014<br>(－4 80)
[illegible]	－0 0042<br>(－1 60)	－0 0045<br>(－1 73)	－0 0035<br>(－1 32)
[illegible]	0 0996<br>(3 44)	0 0989<br>(3 41)	0 1005<br>(3 49)
[illegible], －1	－0 0535<br>(－1 71)	－0 0539<br>(－1 72)	－0 0544<br>(－1 74)
[illegible]	－0 0006<br>(－0 11)	－0 0004<br>(－0 08)	－0 00004<br>(－0 01)
[illegible]检验值	160 15	209 41	162 55
摩值	0 00	0 00	0 00
[illegible]	0 109	0 109	0 112

注: 、和 分别表示1%、5%和10%的显著性水平,括号内为犻值,经过[illegible]异方差调整。

七、主要结论与政策建议

在股权集中、投资者法律保护不足的市场上,公司治理面对的主要问题是控制性大股东与外部中小投资者之间的代理问题([illegible], 1997)。本文从公司投资决策与投资行为视角,对这一代理问题进行研究。理论上,本文首次通过两阶段动态模型,系统探讨了终极控股股东控制权对公司投资决策的影响,研究结果表明,股权集中、控股股东的存在导致了公司过度投资,控股股东控制权与现金流权的分离进一步加剧了过度投资,同时也可以看到,随着自由现金流水平的提高、控制权私利的增加,公司过度投资变得更严重;而控股股东现金流权水平的提高,公司治理机制的改善,则可以有效抑制过度投资。这一研究为中国改革开放30多年来大规模低效率的投资现象提供了一定的理论解释。

(C)1994-2021 China Academic Journal Electronic Publishing House. All rights reserved. http://w

① 我们也使用政府干预指数及市场化指数来度量外部治理环境,所得结论类似。

实证上，本文基于[illegible]（2006）方法，利用2004—2007年中国上市公司样本数据，采用面板数据方法对理论分析进行检验。研究结果表明，控制权与现金流权分离度对过度投资有显著为正的影响，并且随着分离程度的增加，其边际影响减少。同时，自由现金流水平对过度投资影响为正，尽管不显著；相对于私人控股，政府控股情况下过度投资现象更严重。此外，外部治理环境在一定程度上有效抑制了上市公司过度投资。这一系列实证结果基本验证了本文的理论分析，同时也从公司投资行为角度为控股股东侵占提供了新的证据。

表6　公司治理与自由现金流过度投资

	模型8 [illegible]	模型9 [illegible]	模型10 [illegible]
[illegible]	0 0691<br>(1 09)	0 0627<br>(1 00)	0 0679<br>(1 41)
[illegible]	0 0002<br>(1 37)	0 0003<br>(1 48)	0 0002<br>(1 04)
[illegible]	0 0114<br>(2 22)	0 0105<br>(2 04)	0 0115<br>(2 30)
[illegible]	－0 0006<br>(－1 96)	－0 0006<br>(－1 83)	－0 0007<br>(－2 04)
[illegible]	0 0151<br>(2 04)	0 0143<br>(1 94)	0 0104<br>(1 30)
[illegible]	－0 0178<br>(－0 45)	－0 0132<br>(－0 34)	－0 0217<br>(－0 60)
[illegible]	0 0007<br>(2 64)	0 0007<br>(2 60)	0 0005<br>(1 90)
[illegible]	－0 0012<br>(－1 43)	－0 0012<br>(－1 44)	－0 0015<br>(－1 70)
[illegible]检验值	0 62	0 80	11 90
[illegible]值	0 99	0 99	0 156
[illegible]	0 059	0 055	0 027

注：　，　和　分别表示1%，5%和10%的显著性水平，括号内为[illegible]值，经过[illegible]异方差调整。

基于本文的理论和实证研究，我们认为，改变中国改革开放以来大规模低效率的投资现象，需要从两方面着手：一方面需要结合当前股权分置改革，改变股权集中、一股独大的所有权结构，尤其是控股股东通过金字塔结构实现控制权与现金流权分离的现象，在当前投资者法律保护相对不足的情况下，实现内部股权制衡（[illegible]，2000）是一种有效改革措施；另一方面改善法制环境，在进一步完善相关法律条款的同时，着重提高执法效率，从而有效抑制控股股东侵占行为。

参考文献

樊纲、王小鲁、朱恒鹏，2007：《中国市场化指数——各地区市场化相对进程2006年度报告》，经济科学出版社。

胡建平、干胜道，2007：《钱多办"坏事"：自由现金流量与过度投资》，《当代财经》第11期。

雷光勇、刘慧龙，2007：《市场化进程、最终控制人性质与现金股利行为——来自中国犄股公司的经验证据》，《管理世界》第7期。

林毅夫、刘明兴、章奇，2004：《政策性负担、政府干预与企业债务期限结构——来自中国上市公司的经验证据》，《管理世界》第8期。

刘芍佳、孙霈、刘乃全，2003：《终极产权论、股权结构及公司绩效》，《经济研究》第3期。

沈艺峰、况学文、聂亚娟，2008：《终极控股股东超额控制与现金持有量价值的实证研究》，《南开管理评论》第11期。

王化成、李春、卢闯，2007：《控股股东对上市公司现金股利影响的实证研究》，《管理世界》第1期。

王鹏、周黎安，2006：《控股股东的控制权、所有权与公司绩效：基于中国上市公司的证据》，《金融研究》第2期。

王鹏，2008：《投资者保护、代理成本与公司绩效》，《经济研究》第2期。

肖珉，2005：《自由现金流量、利益输送与现金股利》，《经济科学》第2期。

杨华军、胡奕明，2007：《制度环境与自由现金流的过度投资》，《管理世界》第9期。

(C)1994-2021 China Academic Journal Electronic Publishing House. All rights reserved. http://w

[illegible], 2002, "[illegible]", [illegible] 57, 2695—2740.

[illegible], 2002, "[illegible]", [illegible] 117, 121—148.

[illegible], 2006, "[illegible]", [illegible] 82, 343—386.

[illegible], 2000, "[illegible]", [illegible] 58, 81—112.

[illegible], 2002, "[illegible]", [illegible] 57, 2741—2771.

[illegible], 2001, "[illegible]", [illegible] 91, 1—25.

[illegible], 2003, "[illegible]", [illegible]

[illegible], 1998, "[illegible]", [illegible] 36, 193—225.

[illegible], 2000, "[illegible]", [illegible] 90, 22—27.

[illegible], 2000, "[illegible]", [illegible] 58, 141—186.

[illegible], 2005, "[illegible]", [illegible] 3633.

[illegible], 1999, "[illegible]", [illegible] 54, 471—517.

[illegible], 2002, "[illegible]", [illegible] 57, 1147—1170.

[illegible], 2003, "[illegible]", [illegible] 58, 1445—1468.

[illegible], 1998, "[illegible]", [illegible] 88, 422—427.

[illegible], 2006, "[illegible]", [illegible] 11, 159—189.

[illegible], 2002, "[illegible]", [illegible] 66, 3—27.

[illegible], 1990, "[illegible]", [illegible] 26, 3—27.

[illegible]

[illegible]

(1 [illegible];

2 [illegible])

[illegible] (2006), [illegible] 2004—2007 [illegible]

[illegible]

[illegible]

[illegible] 31, [illegible] 34, [illegible] 38

(责任编辑:唐寿宁)(校对:昱 莹)

(C)1994-2021 China Academic Journal Electronic Publishing House. All rights reserved. http://w
