# Supplementary material for: How does equity restriction affect innovation quality? Evidence from listed manufacturing companies in China
Source: PLoS One. 2023 Dec 7;18(12):e0295553. doi: 10.1371/journal.pone.0295553 (PMC10703261; doi:10.1371/journal.pone.0295553)
Supplement: S1 Dataset — (ZIP) [file pone.0295553.s001.zip › Supporting information/S1 Dataset/╩╨│í╗»╓╕╩2/╩2╛▌╦╡├≈ú¿ ╩╨│í╗»╓╕╩21997-2022ú⌐.docx]

**市场化指数/市场化指数分享/含计算原始代码！**

1、数据来源：见文件说明

2、时间跨度：1997-2022年

3、区域范围：全国

4、指标说明：

中国分省份市场化指数（简称“市场化指数”）是一个用指数形式衡量全国各省、自治区和直辖市市场化相对进程的指数体系，目前涵盖了全国31个省（23）、自治区（5）、直辖市（4）（以下简称“省份”）在1997—2022年的市场化相对进程总体评分及排序、各方面指数和分项指数评分及排序（西藏个别年份数据暂缺）。更新的数据正在形成中。此次分享的市场化指数包含总指标以及各个分项：政府与市场关系、非国有经济发展、产品市场的发育程度、要素市场的发育程度、市场中介组织的发育和法律制度环境。此外，此次分享还包含计算过程的stata do文件，以及相关计算数据，方便大家使用。

部分数据如下：


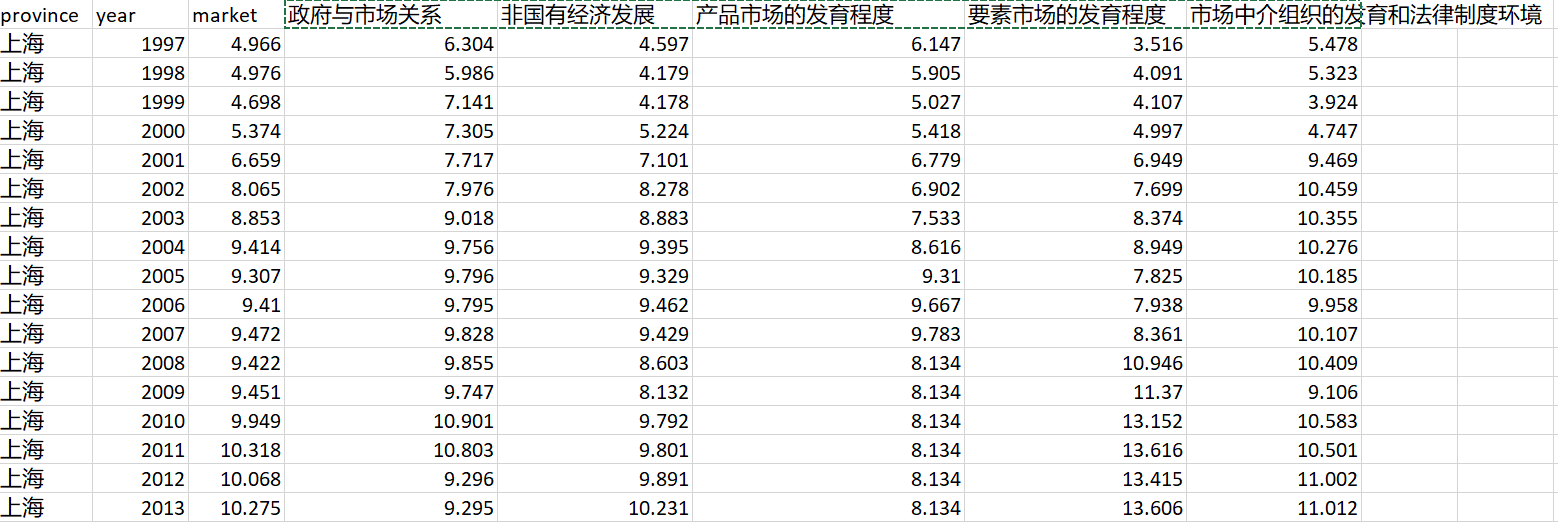


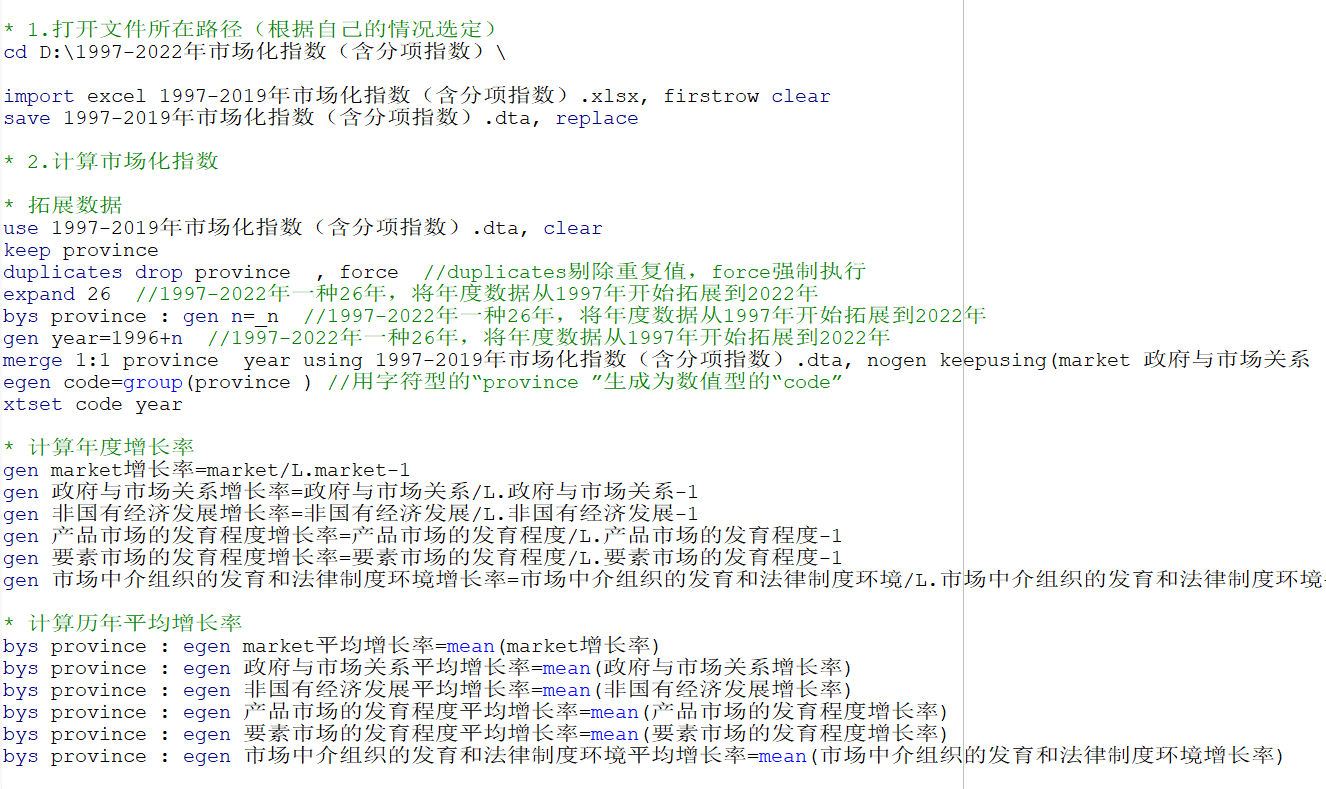


**计算参考文献：**

[1]解学梅, 朱琪玮. 企业绿色创新实践如何破解"和谐共生"难题?[J]. 管理世界, 2021, 37(1):23.

[2]俞红海, 徐龙炳, 陈百助. 终极控股股东控制权与自由现金流过度投资[J]. 经济研究, 2010(8):12.

**相关研究：**

[1]赵茂, 杨洋, 刘大鹏. 中国金融市场化指数的度量研究[J]. 统计与决策, 2019, v.35;No.526(10):151-154.

[2]中国各地区市场化相对进程报告[J]. 经济研究, 2003(3):9-18.

[3]曾繁华, 吴阳芬. 财政分权,市场化与经济增长的实证研究[J]. 统计与决策, 2020, v.36;No.549(09):96-101.

[4]范海峰, 胡玉明. 机构投资者持股与公司研发支出——基于中国证券市场的理论与实证研究[J]. 南方经济, 2012, 000(009):60-69.
